# Low‐Background His‐Tag‐Targeting Probes for Turn‐On Fluorescence Detection of Cell Surface Proteins and Their Binding Interactions

**DOI:** 10.1002/smll.202411730

**Published:** 2025-07-04

**Authors:** Pragati Kishore Prasad, Suraj Toraskar, Suman Khan, Tom Granot, Yael Fridmann Sirkis, Eliane Hadas Yardeni, Shira Albeck, Tamar Unger, Ekaterina Petrovich‐Kopitman, Yoseph Addadi, Rakesh Raigawali, Saurabh Anand, Sharath S. Vishweshwara, Chethan D. Shanthamurthy, Noa Oppenheimer‐Low, Raghavendra Kikkeri, Ori Avinoam, Leila Motiei, David Margulies

**Affiliations:** ^1^ Department of Chemical and Structural Biology Weizman Institute of Science Rehovot 7610001 Israel; ^2^ Department of Biomolecular Sciences Weizman Institute of Science Rehovot 7610001 Israel; ^3^ Life Sciences Core Facilities Weizmann Institute of Science Rehovot 7610001 Israel; ^4^ Indian Institute of Science Education and Research Pune 411008 India; ^5^ Present address: School of Nano Science and Technology Indian Institute of Technology Kharagpur West Bengal 721302 India

**Keywords:** His‐tag binding probe, protein surface recognition, receptor‐ligand interaction, thiazole orange, turn‐on fluorescent probes

## Abstract

Turn‐on fluorescent probes consisting of dye‐ligand conjugates serve as a powerful tool for detecting cell surface proteins (CSPs) and their interactions with binding partners. However, generating such probes from protein‐based ligands remains challenging. This challenge became particularly evident during the COVID‐19 pandemic, which highlighted the need for assays to evaluate inhibitors of the interaction between the SARS‐CoV‐2 virus receptor‐binding domain (RBD) and the angiotensin‐converting enzyme 2 (ACE2) receptor. To sense this interaction in a cellular environment using turn‐on probes, a tri‐nitrilotriacetic acid (tri‐NTA) unit was conjugated to quinoline‐based cyanine (QBC) dyes. This design leverages the high affinity of tri‐NTA for His‐tag, along with the low‐background and confinement‐sensitive optical responses of QBC dyes, to create probes that fluoresce upon binding to His‐tagged proteins on cell surfaces. Herein, it is shown that this approach enables the development of an exceptionally simple cell‐based assay with which inhibitors of the RBD‐ACE2 interaction can be readily sensed by combining a turn‐on probe, His‐tagged RBD, ACE2‐expressing cells, and recording changes in the probe's emission spectra. The potential of this method is further demonstrated by using such probes to detect lectin binding to cell surface glycans and to image a bacterial CSP under wash‐free conditions.

## Graphical abstract

Conjugating a tri‐nitrilotriacetic acid unit to quinoline‐based cyanine dyes generated probes that fluoresce upon binding to His‐tagged proteins on cell surfaces. Using the thiazole orange‐bearing probe, the binding of the receptor binding domain (RBD) of the SARS‐CoV‐2 virus to host cells is readily detected, enabling the straightforward identification of a heparan sulfate analog that disrupts this interaction.

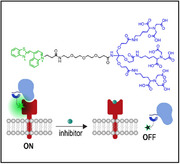

## 1 Introduction

**Figure 1:**
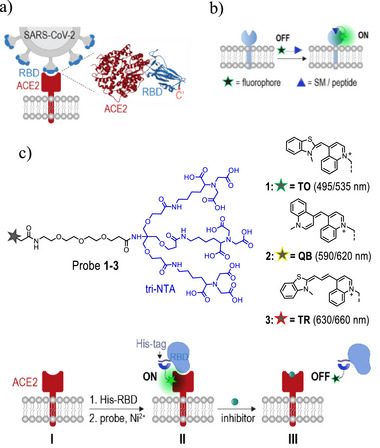
a) Crystal structure of the ACE2‐RBD complex. PDB: 6m0j. b) Schematic illustration of CSP detection using turn‐on fluorescent probes comprising dye‐SM or dye‐peptide conjugates. c) Chemical structures (top) and expected operating principles (bottom) of low‐background His‐tag binding probes (probes **1**–**3**). These probes are designed to show increased emission following interaction with ACE2‐bound His‐RBD in the cells (I→II) and decreased emission following the subsequent addition of an inhibitor (II→III).

The eruption of the COVID‐19 pandemic has sparked interest in developing methods for disrupting the entry of the SARS‐CoV‐2 virus into host cells. Accordingly, much attention has been directed toward developing synthetic agents that inhibit the interaction between the receptor‐binding domain (RBD) of SARS‐CoV‐2, a key component of the spike (S) glycoproteins, and the angiotensin‐converting enzyme 2 (ACE2) receptor on host cells^[^ ^1^ ^]^ (**Figure** **1**a). These advancements have also led to the implementation of various assays for tracking the RBD‐ACE2 protein‐protein interaction (PPI) and assessing the efficiency of such inhibitors.^[^ ^2^ ^]^

Although such assays have been successfully employed in COVID‐19 drug research, they also highlighted general limitations associated with current methods for detecting inhibitors of the interactions between cell surface proteins (CSPs) and their protein ligands. For example, in vitro assays, such as ones based on fluorescence anisotropy or surface plasmon resonance (SPR),^[^ ^3^ ^]^ generally rely on a recombinant extracellular domain of the CSP rather than a full‐length protein. This dependence on non‐native structures, combined with the absence of off‐target proteins in the medium, often leads to the identification of inhibitors that less effectively disrupt the PPIs in a physiologically relevant environment. Conventional molecular biology tools, in which the cell‐bound protein‐ligand is labeled with a fluorescent dye or a fluorescent antibody,^[^ ^4^ ^]^ can be used to detect CSP‐ligand interactions in cells. However, these methods typically require overnight adherence to solid support, cell fixation, and extensive washing to remove excess of the labeling moiety. To simplify the detection of these interactions, CSPs can be fused with reporting modules that trigger cell luminescence upon ligand binding.^[^ ^5^ ^]^ A drawback of such systems, however, is that they require expertise in genetic cell engineering and involve fusing CSPs to large fluorescent proteins or enzymes, which may interfere with their normal function.

In recent years, a simple and efficient method for detecting CSPs and their binding interactions in a native cellular environment has emerged. Instead of labeling the ligand with a fluorophore or genetically modifying the cells, this approach utilizes “turn‐on” fluorescent probes consisting of fluorogenic dye‐ligand conjugates.^[^ ^6^ ^]^ Binding such a probe to its CSP target alters the molecular environment of the dye, resulting in enhanced fluorescence (Figure 1b). Consequently, displacement of the CSP‐bound probe by a natural ligand or a synthetic competitor can be readily detected by observing a reduction in the emission signal.^[^ ^6a,b^ ^]^ Although this approach has proven effective in detecting CSP‐ligand or CSP‐inhibitor interactions, these probes typically comprise small‐molecule (SM) or peptide‐based ligands rather than large proteins or protein domains such as the SARS‐CoV‐2 RBD (Figure 1a).

The difficulty in detecting the binding of CSPs to protein‐based ligands using dye‐ligand conjugates results from two main challenges: The first challenge involves the intricacy of modifying the ligand with a fluorogenic dye in a domain that does not participate in the interaction but undergoes significant structural or environmental changes upon binding to the CSP. A second challenge lies in preventing the dye from fluorescing following its conjugation to the protein‐based ligand. Fluorogenic dyes used to create turn‐on probes for protein detection are intended to fluoresce once brought near the target protein.^[^ ^6^, ^7^ ^]^ Therefore, one would expect that linking such dyes to a protein‐based ligand would render the ligand‐dye conjugate inherently fluorescent.

To address these challenges, we designed fluorescent molecular probes that integrate a tri‐nitrilotriacetic acid (tri‐NTA) unit and quinoline‐based cyanine (QBC) dyes (Figure 1c, probes **1**–**3**). We anticipated that the high affinity of the tri‐NTA group for the hexahistidine tag (His‐tag),^[^ ^8^ ^]^ along with the low‐background and confinement‐sensitive optical responses of QBC dyes,^[^ ^9^ ^]^ would enable these probes to fluoresce only when the His‐tagged proteins are located on the cell surfaces. Herein, we describe how these design principles have led to the development of three turn‐on, His‐tag binding probes (probes **1**–**3**) whose ability to respond to His‐tags on cell surfaces was initially demonstrated by their enhanced emission upon binding to a His‐tagged bacterial CSP. Most importantly, we show that with the thiazole orange (TO)‐bearing probe (probe **1**), the binding of a His‐tagged RBD (His‐RBD) to ACE2 in living cells can be straightforwardly detected (Figure 1c). This capability enabled the creation of a live‐cell assay that is exceptionally simple to prepare and operate, eliminating the need for covalent modification of RBD, genetic modification of ACE2, overnight cell adherence, fixation, washing, and the use of imaging tools. Instead, with probe **1**, the RBD‐ACE2 interaction can be readily sensed using His‐RBD, ACE2‐expressing cells, and a fluorescence plate reader. The effectiveness of this method was further demonstrated by identifying an RBD‐targeting inhibitor based on a heparan sulfate analog (HSA), which was shown to disrupt SARS‐CoV‐2 pseudovirus entry into host cells, as well as by using probe **1** to detect the binding of a His‐tagged lectin to cell‐surface glycans.

## 2 Results and Discussion

Inspecting the crystal structure of the ACE2‐RBD complex reveals that the C′‐terminus of the RBD does not participate in the interaction (Figure 1a), making its position suitable for dye modification. A simple and efficient means to modify a protein with a fluorescent dye in a well‐defined position is by fusing it to a His‐tag and linking the dye to a tri‐NTA functionality to create a His‐tag‐binding probe.^[^ ^8^ ^]^ In the presence of nickel ions, this probe binds to the His‐tag of the protein with high affinity and selectivity through the coordination of Ni^2^⁺ ions with the NTA units and the imidazole groups of the histidine residues. Accordingly, we expected that by fusing the C′‐terminus of the RBD to a His‐tag and linking tri‐NTA to a suitable dye, it should be possible to achieve a probe capable of sensing the RBD‐ACE2 interaction in living cells (Figure 1c). According to our design, the binding of the probe to a His‐RBD should not trigger the emission of the dye. However, once the His‐RBD binds to the ACE2 receptor in the host cell, the molecular environment of the dye changes, leading to a turn‐on fluorescence response (Figure 1c and I→II). In this way, inhibitors of this interaction could be straightforwardly detected by observing a reduction in the emission signal (Figure 1c and II→III).

A critical aspect in the design of such probes is the selection of an appropriate fluorogenic dye. In our previous work, we have linked tri‐NTA to solvatochromic dyes to generate probes that optically respond to changes in the properties of His‐tagged proteins, for example, alterations in the conformation,^[^ ^8f^ ^]^ glycosylation,^[^ ^8g^ ^]^ or expression levels.^[^ ^8h^ ^]^ These studies,^[^ ^8f–h^ ^]^ along with related work from our group on solvatochromic dye‐based protein detection,^[^ ^10^ ^]^ also highlighted the potential limitations of using such dyes to detect the binding of His‐tagged protein ligands to their CSP partners in cells. First, these dyes often exhibit substantial fluorescence in their initial state, resulting in a significant background signal from unbound probes. Additionally, they tend to increase their emission when brought into proximity with proteins, which could lead to even stronger background fluorescence upon binding to His‐RBD.

To address these drawbacks, we selected QBC dyes^[^ ^9a^ ^]^—thiazole orange (TO), quinoline blue (QB), and thiazole red (TR)—as fluorescent reporters for creating turn‐on His‐tag binding probes (Figure 1c, probes **1**–**3**). One reason for this selection is that QBC dyes are hardly fluorescent in an unbound state and exhibit remarkable turn‐on fluorescence once their torsional motion is restricted, typically through DNA intercalation.^[^ ^9a^ ^]^ This property, which is essential for reducing background fluorescence from excess probes in solution, has been employed by Seitz and colleagues in developing low‐noise force intercalation probes (FIT‐probes)^[^ ^9^, ^11^ ^]^ for detecting nucleic acids in cells under wash‐free conditions. Similarly, we have shown that linking TO to SM‐based ligands affords turn‐on probes that can sense proteins with a high signal‐to‐noise (S/N) ratio.^[^ ^12^ ^]^ Another reason for choosing these dyes is that they require a highly restrictive environment to become fluorescent.^[^ ^13^ ^]^ This feature makes QBC‐based probes highly sensitive to subtle structural differences in bioanalyte structures or environments. For example, FIT‐probes have demonstrated the ability to detect single mismatched sequences.^[^ ^11^, ^14^ ^]^ Likewise, we have demonstrated that certain turn‐on probes based on TO‐SM conjugates, which bind to different members of an isoform family, optically respond to only a single protein isoform.^[^ ^12^ ^]^ This suggests that QBC dyes do not readily fluoresce when brought near protein surfaces and could therefore remain dark when attached to a His‐tagged protein‐ligand.

We hypothesized that these characteristics would enable probes **1**–**3** to distinguish between free and ACE2‐bound His‐RBD (Figure 1c). Specifically, we reasoned that due to the small size of His‐RBD, its interaction with the probes is less likely to restrict the torsional motion of the dye, making the His‐RBD‐bound probes weakly fluorescent. However, once the His‐RBD is transferred to a crowded cell surface environment, it should provide more surface area for the dye to interact with, for example, by binding to ACE2 or glycans. This should substantially increase the likelihood that the movement of the dye would be restricted, triggering a turn‐on fluorescence response. A recent report on the enhanced TO emission when integrated into cell surface glycans strongly supports this hypothesis.^[^ ^15^ ^]^

By preparing three tri‐NTA‐QBC dye conjugates (Figure 1c, probes **1**–**3**), we aimed to enhance the color variability of the probes and, more importantly, increase the likelihood that one of them would fluoresce upon binding of the tri‐NTA unit to the His‐tag of RBD on the cell surface. In addition to its high affinity and selectivity for His‐tags, an advantage of using tri‐NTA as a recognition element lies in its cell impermeability, which should prevent the probes from generating background signals due to non‐specific interactions with intracellular proteins. A flexible tri‐ethylene glycol spacer between the tri‐NTA unit and the dyes was inserted to facilitate their interaction with cell surface components.

**Figure 2:**
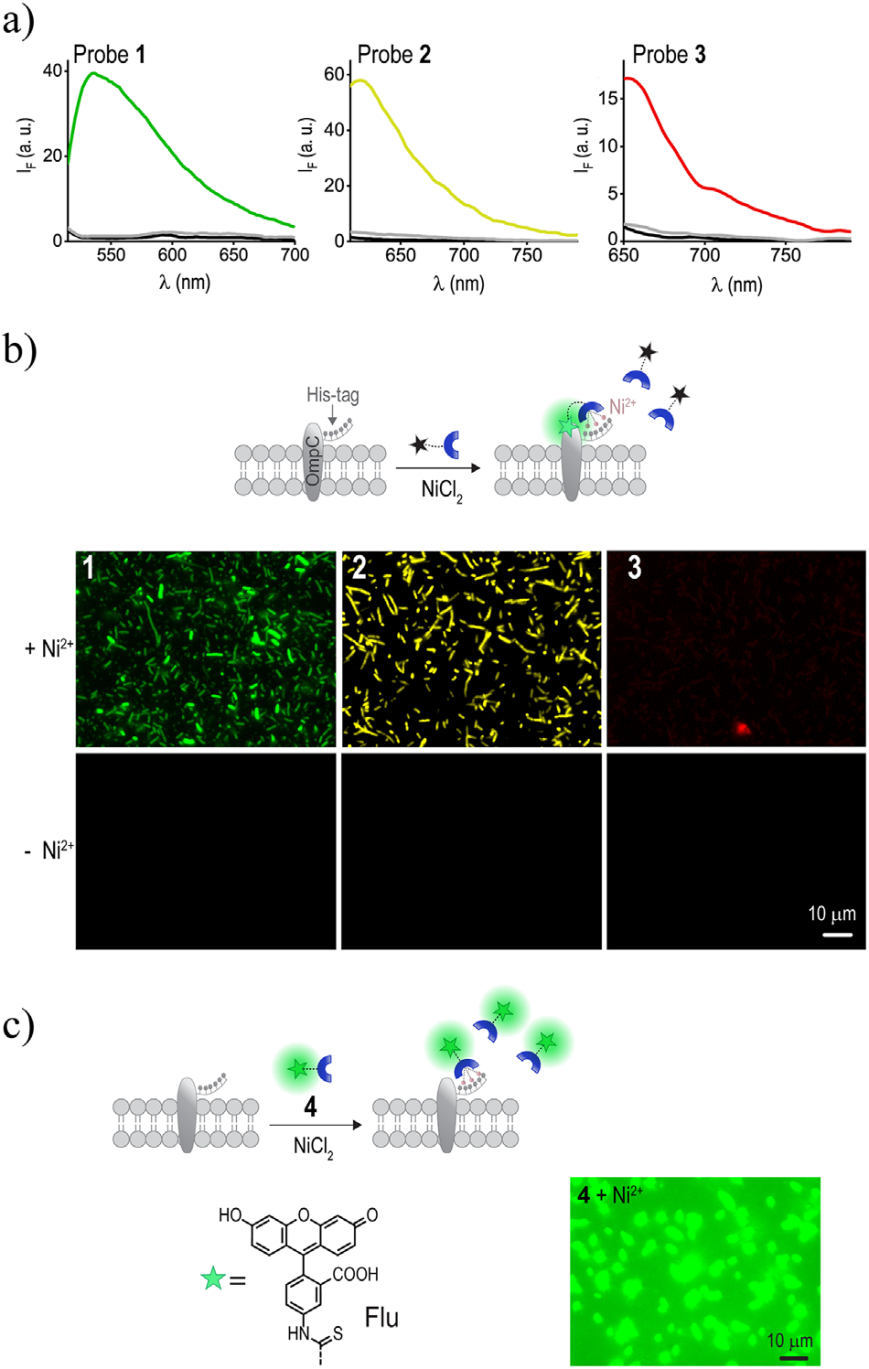
a) Fluorescence spectra of probes **1**–**3** (100 nM) in the absence (black line) and presence of the His‐bacteria (gray line) or the His‐bacteria and Ni^2+^ (colored lines). Bacterial autofluorescence was subtracted from the spectra obtained at 490 nm excitation for clarity. b) Top: Schematic illustration of the wash‐free labeling of His‐OmpC‐expressing bacteria (His‐bacteria) using turn‐on probes **1**–**3**. Bottom: Fluorescence images of His‐bacteria treated with probes **1**–**3** (500 nM) in the presence (upper panel) and absence (lower panel) of Ni^2^⁺ under wash‐free conditions. c) Illustration of wash‐free labeling of the His‐bacteria using “always‐on” probe **4** (left) and the corresponding fluorescence image (right).

To determine whether the emission of the probes increases in a conformationally restricting environment, as expected on cell surfaces, we first measured their fluorescence response to increasing viscosity (Figure S1, Supporting Information). The low emission in aqueous solution and the gradual increase in fluorescence upon the addition of up to 90% glycerol confirmed the probes’ sensitivity to motion‐restricting conditions. Next, we determined whether these photophysical properties—specifically, a minimal background signal and a strong emission under conformational constraints—would enable the probes to fluoresce upon binding to His‐tag fusion peptides on cell surfaces. To this end, we used a fluorescence plate reader to measure their emission spectra before and after incubation with *E. coli* expressing His‐tagged outer membrane protein C (His‐OmpC) (**Figure** **2**a). Additionally, we used a fluorescence microscope to image the His‐OmpC expressing bacteria (His‐bacteria) (Figure 2b,c). His‐OmpC was chosen as the target CSP based on our previous work, in which we labeled it with tri‐NTA‐dye conjugates containing either an “always on” fluorescein (Flu) (Figure 2c, probe **4**), Cy5 (Figure S2, probe **5**, Supporting Information) or a “turn‐on” solvatochromic Nile red (NR) dye (Figure S3, Supporting Information).^[^ ^8h^ ^]^ We reasoned that using the His‐bacteria would allow us to compare the fluorescence responses of probes **1**–**3** with those of the previously developed His‐OmpC targeting probes,^[^ ^8h^ ^]^ and subsequently, evaluate whether they exhibit an improved S/N ratio.

The emission spectra (Figure 2a) revealed that probes **1**–**3** are hardly fluorescent in their initial state and that binding to the His‐ bacteria induces 47‐, 55‐, and 12‐fold enhancements in their fluorescence, respectively. Almost no change in fluorescence was observed in the absence of nickel ions, confirming that the turn‐on response results from a Ni^2+^‐mediated interaction. These results indicate that probes **1**–**3** are more efficient than our previously developed turn‐on, NR‐appended probe (tri‐NTA‐NR conjugate),^[^ ^8h^ ^]^ which generated higher background fluorescence and exhibited only a 4‐fold fluorescence enhancement (at 655 nm) upon incubation with the His‐bacteria (Figure S3, Supporting Information). The effectiveness of probes **1**–**3** was further evaluated by imaging the His‐bacteria under wash‐free conditions (Figure 2b, upper panel). The minimal background emission from unbound probes, clear visualization with probes **1** and **2** (signal to background, SBR = 6), and the inability to image the bacteria without Ni^2^⁺ (Figure 2b, lower panel) provide additional evidence that these probes optically respond to the binding to His‐tags on cell surfaces. For comparison, we repeated this experiment with the “always‐on” probe **4** (Figure 2c), which exhibits high emission in its initial state, a signal that was hardly affected by the addition of His‐tagged bacteria (Figure S3, Supporting Information). Unlike probes **1** and **2**, probe **4** produced blurry images without washing (SBR = 2), and its resolution could only be improved after removing the excess probe from the solution (Figure S4, Supporting Information, SBR = 6).

**Figure 3:**
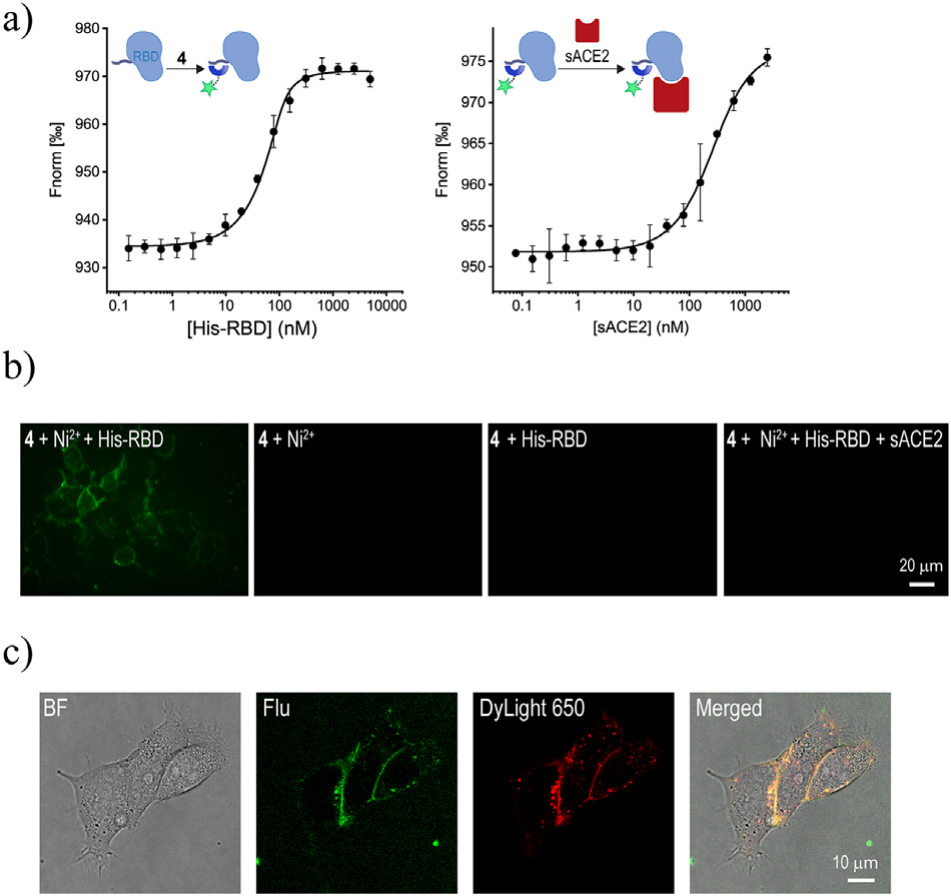
a) MST binding curves generated by incubating probe **4** (100 nM) with increasing concentrations of His‐RBD in the presence of Ni^2+^ (left) and by subjecting the **4**‐His‐RBD complex to increasing concentrations of sACE2 (right). b) Fluorescence images of ACE2‐expressing cells subjected to His‐RBD, **4**, and Ni^2+^(left), **4** and Ni^2+^ (middle left), His‐RBD and **4** (middle right), or His‐RBD, **4**, Ni^2+^, and sACE2 (right), followed by washing. c) Confocal images of HEK293T cells complexed with DyLight650‐modified His‐RBD, followed by the addition of probe **4** and Ni^2^⁺ and subsequent washing. Shown are the transmitted images (left), fluorescent emission confocal slices captured in the Flu or DyLight650 channels (middle), and their overlay (right). Manders' correlation coefficient: 0.62.

After verifying that all three probes exhibit a minimal background signal and can generate robust fluorescence when bound to His‐tags on cell surfaces, we next aimed to determine whether these properties would enable the probes to detect the ACE2‐His‐RBD interaction. An essential feature of our design, in addition to obtaining a high S/N ratio, is that the probes' tri‐NTA units would strongly bind to His‐RBD and that the probe‐His‐RBD complex could subsequently interact with ACE2. A challenge in studying these interactions with probes **1**–**3** is that they are non‐fluorescent in their unbound state and should remain dark upon binding to His‐RBD, making them incompatible with various fluorescence binding assays. Therefore, to study the tri‐NTA‐His‐RBD‐ACE2 interactions, we initially employed the “always‐on” probe **4** (Figure 2c). This probe enabled us to monitor these interactions in vitro using microscale thermophoresis (MST) (**Figure** **3**a). Additionally, it enabled us to assess the effectiveness of labeling the ACE2‐bound His‐RBD in cells via nickel‐coordination compared to conventional methods, where the RBD is covalently modified with a fluorescent dye (Figure 3b). MST binding curves confirm that probe **4** binds strongly to His‐RBD (*K*_d_ = 9 ± 2 nM) (Figure 3a, left) and that the resulting **4**‐labeled His‐RBD can then bind to a soluble ACE2 (sACE2) (*K*_d approx_ = 167 ± 62 nM) (Figure 3a, right). To assess whether this interaction also occurs in cells, ACE2‐expressing HEK297T cells were imaged (Supporting Information) following treatment with probe **4**, His‐RBD, and Ni^2+^ and washing (Figure 3b, left). As controls, we also imaged cells that were subjected to **4** and Ni^2+^ without His‐RBD (middle‐left), **4** and His‐RBD without Ni^2+^(middle‐right), or **4**, His‐RBD, Ni^2+^, and sACE2, which served as an inhibitor^[^ ^16^ ^]^ (right). The fluorescence cell labeling observed only in the first experiment (Figure 3b, left) indicates that the tri‐NTA unit binds to the ACE2‐bound His‐RBD on the host cells. To confirm that this nickel‐mediated cell labeling (Figure 3b, left) reflects the selective binding of **4** to the His‐RBD, we also analyzed confocal microscopy images of cells that were complexed with a DyLight650‐modified His‐RBD and subsequently treated with **4** (Figure 3c). Notably, in this system, the DyLight650 dye was covalently attached to His‐RBD (Supporting Information). The overlap of fluorescence signals in the Flu and DyLight650 channels indicates the association of the probe with the cell‐bound His‐RBD. These cell imaging experiments (Figure 3b,c) also highlighted the limitations of characterizing cell‐bound protein ligands using “always‐on” fluorophores (e.g., Flu or DyLight650), since these methods require overnight cell adherence and washing to remove the unbound probe.

**Figure 4:**
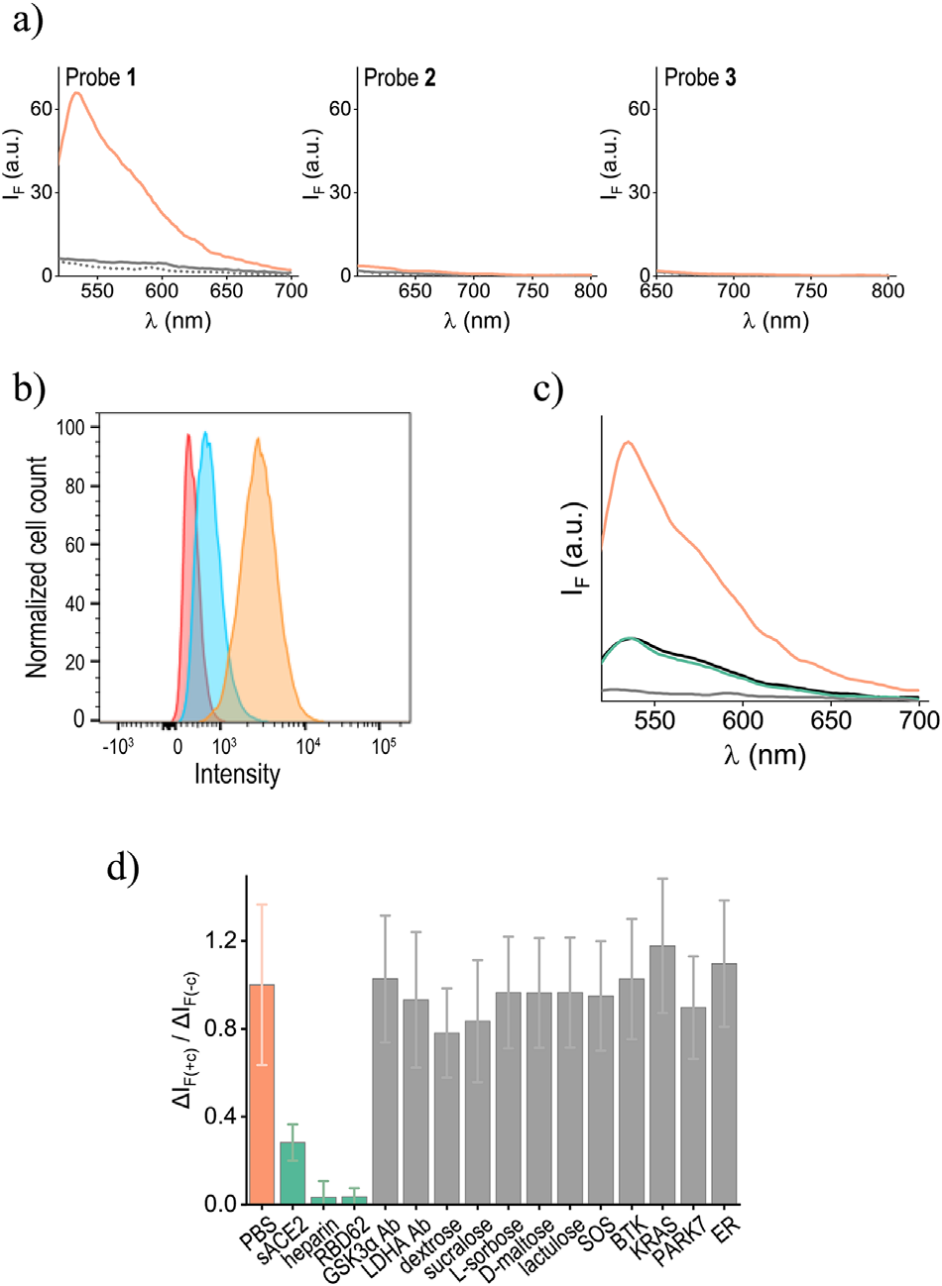
a) Emission spectra of probes **1**–**3** (300 nM) in the absence (dashed line) and presence of His‐RBD (300 nM) (solid gray line) or ACE2‐expressing HEK293T cells pretreated with His‐RBD (orange line). b) Representative flow cytometry histograms of cells before (red) and after incubation with **1** (blue) or with His‐RBD and then **1** (orange). c) Fluorescence spectra of **1** in the absence (grey line) and presence of His‐RBD‐complexed cells (orange line), followed by the addition of heparin (green line). Also shown is the spectrum of **1** in the presence of HEK293T cells without His‐RBD (black line). d) Relative fluorescence responses of probe **1** to the addition of His‐RBD‐complexed cells (orange) and subsequent incubation with known ACE2‐RBD interaction inhibitors: sACE2, RBD62, and heparin (green), or with randomly selected short saccharides and proteins (gray). Protein and carbohydrate concentrations are 300 nM and 100 µM, respectively. ΔI_F(+c)_ and ΔI_F(‐c)_ correspond to the fluorescence response with or without a compound, respectively. All measurements were performed in the presence of Ni^2^⁺.

To test whether probes **1**–**3** could overcome these challenges and enable straightforward detection of the ACE2‐His‐RBD interactions in cells, we used a fluorescence plate reader to measure their emission spectra before and after the addition of His‐RBD or His‐RBD bound to ACE2‐expressing cells in the presence of Ni^2+^ (**Figure** **4**a). The fluorescence spectra confirmed that adding His‐RBD to probes **1**–**3**, in the absence of cells, did not enhance their emission, consistent with our design. Most importantly, the spectra showed that upon incubation with the His‐RBD‐coated cells, the TO‐appended probe (**1**) exhibited the desired turn‐on response, making it a promising candidate for developing a cell‐based assay to detect inhibitors of the ACE2‐RBD interaction. Probes **2** and **3** lack of response suggests that their positioning on the cell surface did not induce proximity‐induced interactions that restrict the dye's intramolecular motion—an effect that could potentially be achieved by modifying the linker length.^[^ ^12^ ^]^ Fluorescence‐activated cell sorting (FACS) revealed effective labeling of the cells that were subjected to both **1** and His‐RBD (Figure 4b, orange histogram), with 91% of the cells being labeled. A weaker fluorescence signal was also observed when the cells were treated with **1** alone (blue histogram), indicating that a portion of the probe's emission arises from some non‐specific interactions, which are likely to occur under wash‐free conditions.

The ability of the **1**/His‐RBD system to readily identify compounds that disrupt RBD binding to ACE2 in cells using a fluorescence plate reader was first tested by measuring its response to known inhibitors (Figure 4c,d). Figure 4c shows the fluorescence spectra from a representative experiment where heparin, known to effectively disrupt the interaction,^[^ ^17^ ^]^ was added to cells treated with His‐RBD and **1**. The results indicate that heparin addition led to a decrease in the emission signal (Figure 4c, green line), as expected from the detachment of the His‐RBD‐**1** complex from the cells (Figure 1c and II→III). Control experiments in which heparin was added to **1** alone confirmed that it did not affect the probe's emission (Figure S5, Supporting Information). The incomplete return of emission to its initial level following heparin addition (Figure 4c, green line) suggests that part of the fluorescence response of **1** to His‐RBD‐coated cells may not involve ACE2 binding. Measuring the fluorescence of **1** after incubation with cells in the absence of His‐RBD (Figure 4c, black line) produced an emission spectrum similar to that observed with heparin treatment. This finding is consistent with the FACS data (Figure 4b), confirming the occurrence of some non‐specific binding and allowing us to establish a baseline for determining the assay's specific fluorescence response (ΔI_F_). The selectivity of the system and its ability to identify inhibitors were further demonstrated by its detection of two additional known inhibitors, RBD62^[^ ^18^ ^]^ and sACE2,^[^ ^16^ ^]^ as well as its lack of response to other randomly selected short saccharides and proteins (Figure 4d).

**Figure 5:**
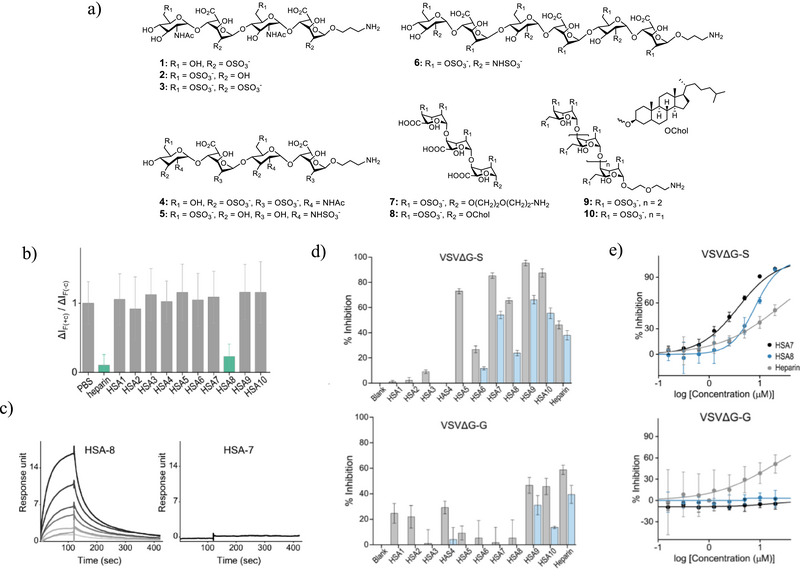
a) Chemical structure of the different HSAs. b) Relative fluorescence responses of the **1**/His‐RBD cell‐based assay to 10 µM of HSA 1–10 or heparin. c) SPR sensorgrams of HSA‐8 (two‐fold serial dilutions from 10 to 0.156 µM) or HSA‐7 (10 µM) binding to immobilized RBD. d) % inhibition of VSVΔG‐S (top) and VSVΔG‐G (bottom) entry into ACE2‐expressing cells by 10 (gray) or 1 µM (blue) of HSA 1–10. e) Dose‐response curves that follow the inhibition of VSVΔG‐S (top) or VSVΔG‐G (bottom) infection by HSA8, HSA7, and heparin.

The ability of heparin to block SARS‐CoV‐2 entry into host cells has generated interest in developing synthetic heparin analogs with enhanced activity and selectivity.^[^ ^17^, ^19^ ^]^ Such analogs could potentially address a major limitation of heparin in treating viral infections, namely, its broad binding to various targets, which reduces its effectiveness and can lead to side effects like anticoagulation. Although heparin can inhibit SARS‐CoV‐2 entry by binding at three sites on the spike (S) glycoprotein,^[^ ^17^ ^]^ the conservation of the heparan sulfate (HS) binding site within the RBD across SARS‐CoV‐2 variants has made RBD‐targeting inhibitors based on HS analogs (HSAs) particularly desirable.^[^ ^20^ ^]^ We reasoned that an assay based on probe **1** could facilitate the target‐based identification of HSAs that inhibit the RBD‐ACE2 interaction in cells. Accordingly, we measured the fluorescence response of our system to ten HSAs that differ in their chain length and sulfation pattern (**Figure** **5**a). One of these analogs, HSA‐8, features a steroidal side chain that may bind to hydrophobic residues of the RBD—a characteristic observed in pixatimod (PG545) which has been shown to inhibit the RBD‐ACE2 interaction.^[^ ^20b^ ^]^ The results show that a decrease in the emission of **1** was observed only for HSA‐8 (Figures 5b), with almost no change in emission for the other HS analogs. To assess whether HSA‐8 binds to RBD, SPR measurements (Supporting Information) were performed. The resulting sensorgrams (Figures 5c‐left; Figure S6, Supporting Information) revealed that HSA‐8 interacts with RBD in a dose‐dependent manner with a K_d_ value of 5.75 µM, thus confirming that the assay successfully identified an RBD‐binding inhibitor.

To determine whether HSA‐8 can inhibit virus penetration as well as provide improved selectivity in blocking SARS‐CoV‐2 entry relative to heparin, we tested it, along with the other analogs and heparin, against two infection‐activated pseudovirus systems.^[^ ^21^ ^]^ These systems, which are based on engineered vesicular stomatitis virus (VSV),^[^ ^22^ ^]^ were designed to express a fluorescent reporter upon infection, enabling analysis by fluorescence microscopy (Figure 5d,e).^[^ ^21^ ^]^ One pseudovirus (VSVΔG‐S) displays the SARS‐CoV‐2 spike (S) glycoprotein on its surface. Notably, this pseudovirus has been established as a robust tool for evaluating inhibitors of SARS‐CoV‐2 entry.^[^ ^21^ ^]^ The second pseudovirus (VSVΔG‐G), used to assess the selectivity of the inhibitors, shares the same backbone but features a G glycoprotein, structurally distinct from the S glycoprotein.^[^ ^23^ ^]^ In our initial experiments, we assessed the impact of HSAs and heparin on VSVΔG‐S infection at two different concentrations, following an established protocol^[^ ^21^ ^]^ (Figure 5d, top). The experimental findings revealed that HSA‐8, along with an additional compound, HSA‐7, substantially inhibited VSVΔG‐S and minimally inhibited VSVΔG‐G (Figure 5d, bottom). Consequently, we selected these compounds for dose‐response experiments to evaluate their potential as antiviral agents targeting the SARS‐CoV‐2 S glycoprotein (Figure 5e). Compounds HSA‐7 and HSA‐8 exhibited IC_50_ values of 3.8 and 8.0 µM, respectively, against VSVΔG‐S (Figure 5e, top), with no activity against VSVΔG‐G. When compared to these compounds, heparin exhibited lower inhibition against VSVΔG‐S (IC_50_ = 24.5 µM) and higher inhibition against VSVΔG‐G (IC_50_ = 17.16 µM), highlighting the improved selectivity and potency of the HSA inhibitors. To better understand the nature of the antiviral activity of HSA‐7, additional SPR measurements were performed (Figure 5c, right). The results indicate that, in contrast to HSA‐8 (Figure 5c, left) and heparin (Figure S7, Supporting Information), and consistent with the lack of fluorescence response observed in the **1**/His‐RBD assays (Figure 5b), HSA‐7 does not interact with RBD.

The observation that HSA‐8 selectively disrupts VSVΔG‐S entry highlights the effectiveness of the **1**/His‐RBD system in identifying potential inhibitors of SARS‐CoV‐2 infection. Conversely, the identification of HSA‐7 as an additional inhibitor demonstrates the advantage of using the VSVΔG‐S system, thus underscoring the complementarity between the two turn‐on, cell‐based assays employed in this study. The assay based on probe **1** (Figure 5b) offers a straightforward, targeted approach for inhibitor development, but it is limited to detecting RBD‐targeting inhibitors and cannot determine whether a detected compound can inhibit virus infection. In contrast, the VSVΔG‐S‐based assay is a phenotypic assay that can detect a broad range of inhibitors of the S glycoprotein function,^[^ ^21^ ^]^ but the specific targets of these inhibitors remain unknown. Moreover, this assay is more complex to perform, requiring a 24 h post‐infection incubation, image processing, and specialized expertise (Supporting Information). These last experiments thus indicate that by combining these two assays, one can readily conclude that inhibition of virus entry by HSA‐8 involves disruption of RBD binding to ACE2, whereas HSA‐7 interferes with infection through other mechanisms, possibly by binding to the S2 domain or the multifunctional S1/S2 site of the S glycoprotein.^[^ ^17^ ^]^

**Figure 6:**
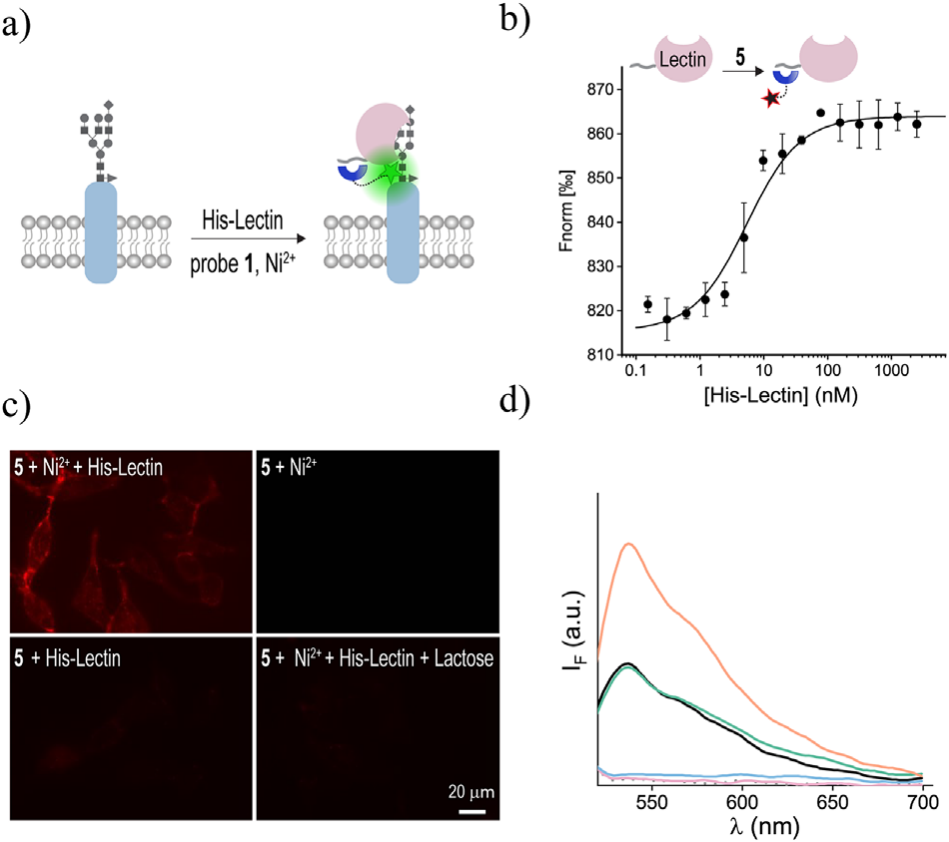
a) Schematic illustration of the turn‐on response of **1** following His‐lectin binding to the cell surface. b) MST binding curves generated by incubating probe **5** (1 nM) with increasing concentrations of the His‐lectin in the presence of Ni^2+^. c) Fluorescence images of HeLa cells subjected to His‐lectin, **5** and Ni^2+^(top left), **5** and Ni^2+^ (top right), His‐lectin and **5** (bottom left), or His‐lectin, **5**, Ni^2+^, and lactose (bottom right), followed by washing. d) Fluorescence spectra of probe **1** before (dashed line) and after incubation with His‐lectin (blue line), lactose (pink line), HeLa cells (black line), HeLa cells with His‐lectin (orange line) or HeLa cells with His‐lectin and lactose (green line).

Following the turn‐on sensing of His‐RBD binding to ACE2 and the subsequent identification of inhibitors, we next aimed to assess whether this approach could be used to detect the binding of other His‐tagged protein ligands to cells. Given that fluorescently labeled lectins are commonly used to label cell surface glycoproteins,^[^ ^24^ ^]^ and that TO's emission has been shown to increase when associated with cell‐surface glycans,^[^ ^15^ ^]^ we tested whether probe **1** could sense the binding of a His‐tagged galactose‐binding lectin (His‐lectin) to HeLa cells (**Figure** **6**a), which are known to exhibit high levels of glycosylation on their surface.^[^ ^25^ ^]^ As was done with His‐RBD, an “always‐on” probe (probe **5**, Figure S2, Supporting Information) was initially applied to confirm tri‐NTA binding to the His‐lectin by MST (Figure 6b), and to cell‐bound His‐lectin using conventional fluorescence microscopy (Figure 6c). The MST binding curve shows that the probe binds strongly to His‐lectin (*K*_d_ = 4.9 ± 0.6 nM) (Figure 6b). Moreover, as expected with lectin‐mediated labeling, the fluorescent cell images show that the HeLa cells were effectively labeled following treatment with the His‐lectin, **5**, and Ni^2+^ (Figure 6c, top left). In contrast, the cells were hardly labeled when incubated with **5** and Ni^2+^ without His‐lectin (top right), **5**, and His‐lectin without Ni^2+^(bottom left), or **5**, His‐lectin and Ni^2+^, in the presence of lactose (right), which competes with lectin binding^[^ ^26^ ^]^ (bottom right). In the next step, we determined whether these binding events could also be followed by recording the emission of turn‐on probe **1** using a simple plate reader (Figure 6d; Supporting Information). The results revealed that the binding of the His‐lectin to HeLa cells could be straightforwardly detected through enhanced emission (orange line), and that in the presence of lactose (green line), the signal remained at its initial baseline level (black line). Together with previous results obtained with His‐OmpC and His‐RBD, these findings demonstrate how turn‐on His‐tag binding probes, such as probe **1**, could expand the current fluorescent toolbox for investigating CSPs and their interactions with protein partners.

## 3 Conclusion

In summary, we have shown that conjugating a tri‐NTA unit to torsionally responsive QBC dyes could lead to the development of molecular probes that fluoresce upon binding to His‐tagged proteins on cell surfaces. The high affinity of tri‐NTA for His‐tags, along with the high S/N ratio and viscosity‐dependent optical responses of QBC dyes, overcomes key challenges in designing turn‐on probes from dye‐labeled protein ligands, enabling their use in the straightforward detection of CSP interactions with His‐tagged protein partners.

The low background and fluorescence activation of the probes upon binding to His‐tagged proteins on cell surfaces were initially demonstrated by their strong response to *E. coli* expressing His‐OmpC, allowing for the imaging of the bacteria under wash‐free conditions. To assess the effectiveness of the probe in detecting medicinally relevant CSP‐ligand interactions, we examined their ability to sense His‐RBD binding to ACE2 in living cells. Our results show that with probe **1**, His‐RBD binding to ACE2‐expressing cells can be readily detected using a fluorescence plate reader that records changes in the probe's fluorescence spectra. This capability led to the development of a cell‐based assay that was used to identify an inhibitor based on a heparan sulfate analog, which was subsequently shown to disrupt virus entry into host cells by interfering with RBD binding. We have also shown that **1** exhibits similar changes in its emission spectrum upon the binding of a His‐lectin to cell surface glycans, indicating the potential for using turn‐on His‐tag binding probes to monitor additional protein–cell interactions. In this regard, a limitation of the current system is that a portion of the emission arises from non‐specific interactions with cells, which reduces its sensitivity. However, given that His‐tag is the most prevalent fusion peptide used in cell biology and that detection of inhibitors with probe **1** was achieved without covalent modification of the protein ligand, genetic modification of the CSP, overnight cell adherence, or imaging tools, we believe that further development of such probes could enable the detection of additional medicinally relevant interactions, potentially facilitating the discovery of novel inhibitors.

## Conflict of Interest

The authors declare no conflict of interest.

## Supporting information



Supporting Information

## Data Availability

The data that support the findings of this study are available in the supplementary material of this article.
