# Supplementary material for: Low‐Background His‐Tag‐Targeting Probes for Turn‐On Fluorescence Detection of Cell Surface Proteins and Their Binding Interactions
Source: Small. 2025 Jul 4;21(33):2411730. doi: 10.1002/smll.202411730 (PMC12372432; doi:10.1002/smll.202411730)
Supplement: Supplementary file 1 — Supporting Information [file SMLL-21-2411730-s001.pdf]

NANO · MICRO
small

# Supporting Information

for *Small*, DOI 10.1002/smll.202411730

## Low-Background His-Tag-Targeting Probes for Turn-On Fluorescence Detection of Cell Surface Proteins and Their Binding Interactions

*Pragati Kishore Prasad, Suraj Toraskar, Suman Khan, Tom Granot, Yael Fridmann Sirkis, Eliane Hadas Yardeni, Shira Albeck, Tamar Unger, Ekaterina Petrovich-Kopitman, Yoseph Addadi, Rakesh Raigawali, Saurabh Anand, Sharath S. Vishweshwara, Chethan D. Shanthamurthy, Noa Oppenheimer-Low, Raghavendra Kikkeri, Ori Avinoam, Leila Motiei* and David Margulies**

# Supporting Information

## Low-Background His-Tag-Targeting Probes for Turn-On Fluorescence Detection of Cell Surface Proteins and their Binding Interactions

Pragati Kishore Prasad,[a] Suraj Toraskar,[a] Suman Khan,[b] Tom Granot,[a] Yael Fridmann Sirkis,[c] Eliane Hadas Yardeni,[c] Shira Albeck,[c] Tamar Unger,[c] Ekaterina Petrovich-Kopitman,[c] Yoseph Addadi,[c] Rakesh Raigawali,[d] Saurabh Anand,[d] Sharath S. Vishweshwara,[d] Chethan D. Shanthamurthy,[d] Raghavendra Kikkeri,[d] Ori Avinoam,[b] Leila Motiei,[a]* and David Margulies[a]*

a. Department of Chemical and Structural Biology, Weizmann Institute of Science, Rehovot 7610001, Israel.
b. Department of Biomolecular Sciences, Weizmann Institute of Science, Rehovot 7610001, Israel
c. Life Sciences Core Facilities, Weizmann Institute of Science, Rehovot 7610001, Israel.
d. Indian Institute of Science Education and Research, Pune 411008, India.

**Table of contents**


| | |
|---|---|
| 1. Materials and methods | 3 |
| 2. Abbreviations | 4 |
| 3. Bacterial strains and growth conditions | 5 |
| 4. Expression and purification of Spike His-RBD | 5 |
| 5. Labeling of RBD Protein with DyLight™ 650 Dye | 5 |
| 6. General procedure for fluorescence measurements with *E. coli* | 5 |
| 7. Fluorescence imaging experiments with *E. coli* | 6 |
| 8. General procedure for fluorescence imaging of ACE2-expressing HEK293T cells | 6 |
| 9. General procedure for testing selected compounds to disrupt ACE2-RBD interaction by measuring the fluorescence spectra | 7 |
| 10. Flow cytometry experiments | 7 |
| 11. Microscale Thermophoresis (MST) | 8 |
| 12. Surface plasmon resonance (SPR) measurements | 9 |
| 13. Pseudovirus-based infection assays | 10 |
| 14. Fluorescence imaging of HeLa cells | 11 |
| 15. Fluorescence assays for detecting lectin binding to HeLa cells | 11 |
| 16. Synthesis of probes **1-3**: Procedures and schemes | 13 |
| 17. Synthesis of HSA analogs: Procedures and schemes | 23 |
| 18. Supplementary figures | 38 |
| 19. NMR spectra of synthesized compounds | 43 |
| 20. References | 80 |

**1. Material and methods:** All solvents and reagents were obtained from commercial suppliers and used without further purification. Dry solvents were purchased from Sigma Aldrich. Deuterated solvents were purchased from Cambridge Isotope Laboratories, Inc. (Andover, MA). The $^{1}$H NMR and $^{13}$C NMR spectra were recorded on a Bruker Advance 300, 400, or 500 MHz spectrometer. The chemical shifts are represented in ppm on the $\delta$ scale down field from TMS as the internal standard. The following abbreviations were used to describe the peaks: br-broad, s-singlet, d-doublet, t-triplet, td-triplet of doublets, q-quartet, quin-quintet, and m-multiplet. The mass spectrum was recorded by a Waters SYNAPT-XS Q-TOF High Resolution mass spectrometer (Manchester, UK) with an electrospray ionization (ESI) interface in the negative ion mode within a mass range from 400 to 5000 m/z. The analytical reversed phase high-performance liquid chromatography (RP-HPLC) analysis was performed on an Agilent Technologies 1260 Infinity quaternary pump LC system, equipped with a diode-array detector using a $C_{18}$ column. Preparative HPLC was carried out using an Agilent 218 purification system, equipped with an autosampler, a UV-Vis dual wavelength detector, and a 440-LC fraction collector operating under OpenLab ChemStation software. Fluorescence was measured using a BioTek synergy H4 hybrid multiwall plate reader, in black flat-bottom polystyrene NBS 384-well microplates (Corning). Heparin was obtained from Hebei Changshan Biochemical Pharmaceutical Co., Ltd. (China). Its concentration was calculated based on an average molecular weight of 15,000 Da. Soluble ACE2 (sACE2), and the His-tagged receptor binding domain of spike protein (His-RBD) were expressed by the Proteomics Unit (Weizmann Institute of Science). RBD-62 and ACE2-Fc were generously provided by Gideon Schreiber and Ron Diskin, respectively (Weizmann Institute of Science). Recombinant prokaryotic lectin RPL was obtained from AMS Biotechnology Ltd (Abingdon, UK). ACE2-expressing HEK-293T cell lines were purchased from ATCC and screened negative for mycoplasma using a PCR-based assay (EZ-PCR mycoplasma detection kit, Biological Industries). The pCAGGS-G plasmid encodes the Vesicular Stomatitis Virus G glycoprotein from the Indiana serotype (VSV-G)[1]. pcDNA3.1-SARS-CoV-2-Spike-C9, encoding the Spike glycoprotein of Wuhan SARS-CoV-2 fused to a C-terminal C9 tag (S) was a kind gift from Fang Li (Addgene plasmid # 145032, http://n2t.net/addgene:145032; RRID:Addgene_145032)[2]. Baby Hamster Kidney cells (BHK-21; ATCC, USA) were maintained in Dulbecco's modified eagle medium (DMEM; Thermo Fisher Scientific, USA) supplemented with 10% fetal bovine serum (FBS; Thermo Fisher Scientific), 1% penicillin-streptomycin (PS, Biological Industries) and 25

mM HEPES (Biological Industries). Human Embryonic Kidney-293T cells overexpressing ACE2 and TMPRSS2 (HEK-293T-ACE2-TMPRSS2) were cultured in DMEM supplemented with 8.1% FBS, 1% PS, 25 mM HEPES and 1.5 μg/ml Blasticidin (InvivoGen, USA).

Bacterial cell images were acquired using an Olympus IX51 fluorescent microscope equipped with a U-MNIBA3 fluorescence filter cube (excitation and emission filters of 470-495 nm and 510-550 nm, respectively, for thiazole orange dye) a U-MNG2 fluorescence filter cube (excitation and emission filters of 530-550 nm and 590 nm, respectively for quinoline blue dye) and a U-MF2 fluorescence filter cube (excitation and emission filters of 620-660 nm and 700-775 nm, respectively for thiazole red dye).

**2. Abbreviations.** Acetonitrile (ACN), dichloromethane (DCM), N,N'-diisopropylethylamine (DIPEA), N,N'-dimethylformamide (DMF), dimethyl sulfoxide (DMSO), 1-Ethyl-3-(3-dimethylaminopropyl) carbodiimide **(**EDC), fluorescein isothiocyanate (FITC), 1-[bis(dimethylamino)methylene]-1H-1,2,3-triazolo[4,5-b]pyridinium3-oxid hexafluorophosphate (HATU), hydroxybenzotriazole (HOBt), methanol (MeOH), MicroScale Thermophoresis (MST), nitrilotriacetic acid (NTA), phosphate buffer saline (PBS), reverse phase high-performance liquid chromatography (RP-HPLC), sodium dodecyl suphate (SDS), trifluoroacetic acid (TFA).

**Full names of the abbreviated proteins and carbohydrates presented in Figure 4**: Bruton's tyrosine kinase (BTK), Estrogen receptors (ER), Glycogen synthase kinase-3 (GSK3), Kirsten rat sarcoma virus (KRAS), Lactate dehydrogenase A (LDHA), Parkinson's disease protein (PARK7), Sucrose octasulfate (SOS).

**3. Bacterial strains and growth conditions.** The bacterial strain used in this study was *E. coli* K-12 strain KRX. Details regarding the expression of the polyhistidine tag at the 7th loop of OmpC were described previously.[3] Transformed bacteria were cultured overnight in LB medium containing ampicillin (100 μg/mL) at 30 °C. The next day, the bacterial cells were diluted 100-fold in fresh LB medium supplemented with ampicillin and incubated until the $OD_{600}$ reached ~ 0.6. Protein expression was then induced by the addition of 0.1% Rhamnose and 20 μM isopropyl-b-D-1-thiogalactopyranoside (IPTG) and cultures were incubated overnight at 37 °C on a shaking plate (230 rpm). The bacterial cells were collected by centrifugation at 6000 × g for 4 min. Pellets

were washed twice with PBS × 1 buffer (100 µL) and then re-suspended in 100 µL in the same buffer to an $OD_{600}$ of 0.3.

**4. Expression and purification of Spike His-RBD.** The SARS-CoV-2 RBD DNA construct (residues 319–541) containing a C-terminal Hexa-histidine tag (a kind gift from Florian Krammer, Icahn School of Medicine at Mount Sinai, NY) was used for protein production in Expi293 cells. The medium containing the secreted protein was collected 72 hours post-transfection for purification. After the cells were removed, the medium was applied to a Ni column (HisTrap FF 5 mL) equilibrated with PBS. The bound RBD was washed with PBS containing 20 mM imidazole and eluted with PBS containing 250 mM imidazole. Fractions containing Spike_RBD-His were pooled and injected into a Superdex 200 HR 10/30 column equilibrated with Tris buffer (20 mM, 200 mM NaCl, pH = 8). The pure RBD-His was eluted at 16.8 mL as a single peak corresponding to the monomeric protein. The protein was characterized by SDS-PAGE, stained with Coomassie blue, and then Western blotted (WB) using an anti-His antibody (anti-polyHistidine peroxidase Ab, mouse monoclonal #A7058, Sigma Aldrich). Aliquots (100 µL each) were flash-frozen in liquid nitrogen and stored at -80°C.

**5. Labeling of RBD Protein with DyLight™ 650 Dye.** To label the RBD protein with DyLight™ 650, 100 µL of RBD (30 µM) was buffer-exchanged into PBS at pH 7.7 using a Zeba Spin desalting column (Thermo Fisher). Next, 14 µL of DyLight™ 650 dye (Thermo Fisher) at a concentration of 650 µM in DMSO was added to the protein and incubated in the dark for 1 hour. Following labeling, excess dye was removed by buffer-exchanging to PBST using a Sephadex G-25 Mini column, resulting in a 75% labeling efficiency. The efficiency was determined by measuring the absorbance of the labeled protein at both 280 nm and 650 nm, using extinction coefficients of 33,850 $cm^{-1}M^{-1}$ for the protein and 250,000 $cm^{-1}M^{-1}$ for the dye.

**6. General procedure for fluorescence measurements with *E. coli.*** A 10 µM sample of probes (**1-3**) was mixed with and without $NiCl_2 \cdot 6H_2O$ (60 µM) in Tris buffer (20 mM, pH = 7.5) and allowed to stand at room temperature for 30 minutes. Meanwhile, bacterial cells with an $OD_{600}$ of 3.0 were transferred to Eppendorf tubes and centrifuged at 6,000g for 4 minutes. After the LB medium was removed, the pellet fraction was washed twice with tris buffer (200 µL each) and finally re-suspended in 200 µL of Tris buffer. Subsequently, each probe (**1-3**), at a final concentration of 100 nM, was added to the bacterial suspension, and the emission spectra were

immediately recorded. The fluorescence responses of probes **1-3** were measured with excitation wavelengths of 495 nm, 590 nm, and 630 nm, respectively. These experiments were conducted in triplicate.

**7. Fluorescence imaging experiments with *E. coli*.** To a 200 μL sample of the bacterial suspension with an OD of 0.3, a preincubated sample of each probe (**1-3**) at a concentration of 500 nM, with or without $NiCl_2$ at 2.5 μM, was added. The cells were then incubated at room temperature for 1 hour. Afterward, each sample was placed onto a poly-l-lysine-coated glass-bottom dish and allowed to adhere for 30 minutes before imaging using an Olympus IX51 fluorescent microscope. The samples were imaged using 100× objective lenses.

**8. General procedure for fluorescence imaging of ACE2-expressing HEK293T cells.** ACE2-expressing HEK293T cells were maintained in DMEM supplemented with 4.5 g/L glucose, 2 mM L-glutamine, 10% (v/v) fetal bovine serum, and 1 μg/mL puromycin in a 5% $CO_2$ incubator at 37 ºC. Cells (30,000 cells/well) were seeded onto glass-bottom culture dishes (35 mm dish, 14 mm microwell, Cat. no: P35G-1.5-14C, MatTek Life Sciences) and allowed to adhere overnight. The following day, the medium was removed, and the cells were rinsed three times with PBS. They were then incubated with His-RBD protein or DyLigh650-labeled His-RBD at a concentration of 2 μM in a 37 ºC, 5% $CO_2$ incubator for 30 minutes. After incubation, excess His-RBD or DyLigh650-labeled His-RBD was gently washed off the cells twice with PBS (containing $Ca^{2+}$ and $Mg^{2+}$). Meanwhile, a mixture of probe **4** (100 µM in PBS) and $NiCl_2 \cdot 6H_2O$ (500 µM in MQ water) was prepared by incubating them together at room temperature for 30 minutes. Cells were then incubated with probe **4** (3 µM in PBS) at 37 ºC and under 5% $CO_2$ for 30 minutes. Next, the cells were washed twice with PBS (containing $Ca^{2+}$ and $Mg^{2+}$) and imaged using an Olympus IX51 fluorescent microscope or a Leica TCS SP8 STED microscope with a 60× objective lens. Control experiments (without His-RBD or $Ni^{2+}$) were performed under the same conditions. For the experiment in which sACE2 was used to inhibit His-RBD binding to cells, the cells were treated with 5 µM sACE2, followed by washing prior to fluorescent imaging. Fluorescence images were acquired with a fixed exposure time of 400 ms in all experiments. Confocal imaging was conducted using an inverted Leica SP8 STED3X microscope, equipped with internal Hybrid (HyD) detectors and an Acusto Optical Tunable Filter (Leica microsystems CMS GmbH, Germany) as well as a White light laser (WLL) excitation wavelength ranging from 470-670 nm. Imaging was performed

with the HC PL APO 86×/1.30 W STED White motCORR objective. Collection channels: Excitation at 488 nm (8% power) with emission collected at 500-560 nm using a HyD hybrid detector, and excitation at 660 nm with emission collected at 670-780 nm using a HyD hybrid detector. Brightfield imaging was captured using a transmitted PMT detector with the same laser excitation line. Images were acquired using a galvanometric scanner at a scan speed of 400 Hz, in a 992 × 992 pixel format (pixel size: 0.083 µm) with Z-steps of 0.356 µm.

**9. General procedure for testing selected compounds to disrupt ACE2-RBD interaction by measuring the fluorescence spectra.** After the cells were cultured overnight, they were treated with trypsin, followed by centrifugation (1000 rpm, 5 min) to pellet the cells. The supernatant containing trypsin was discarded, the cells were re-suspended in fresh media, and then transferred to Eppendorf tubes. Subsequently, the cells were incubated with His-RBD protein at a concentration of 300 nM per $1\times10^5$ cells in PBS in a shaker incubator at 37 ºC and under 5% $CO_2$ for 30 minutes. Unbound His-RBD was removed by washing the cells twice with 100 µL PBS. Then, the cells were incubated with a sample of probe **1** (300 nM) containing $NiCl_2$ (1.5 µM) per $1 \times 10^5$ cells in PBS at 37 ºC and under 5% $CO_2$ for 10 minutes. Next, the cells were transferred to a 386-well black Corning plate, and the fluorescence spectra were recorded. Subsequently, known inhibitors (sACE2, RBD62, and heparin), or randomly selected saccharides and proteins were added to the wells and allowed to incubate for 10 minutes, after which the fluorescence spectra were recorded again. Protein and carbohydrate concentrations were 300 nM and 100 µM, respectively. The emission spectra were measured using an excitation wavelength of 495 nm. The specific fluorescence response of the **1**/His-RBD assay ($\Delta I_{F\ (+c)}$) was calculated by measuring the emission intensity at 535 nm and subtracting the nonspecific background signal, specifically the emission recorded after incubating **1** with cells in the absence of His-RBD.

**10. Flow cytometry experiments.** ACE2-expressing HEK cells were harvested and counted using an electronic cell cytometer. Cells at passages 5 to 10 were used in the experiments. One million ($1 \times 10^6$) cells were transferred to each Eppendorf tube. The medium was removed from the cells and rinsed twice with PBS. Subsequently, the cells were incubated with His-RBD protein at a concentration of 3 µM per $1 \times 10^6$ cells in PBS in a shaker incubator at 37 ºC under 5% $CO_2$ for 30 minutes. Unbound His-RBD was washed away with PBS (2 × 200 µL) by centrifugation at 1,500 rpm for 2 minutes. Each sample of cells was then incubated with probe **1** (3 µM containing

15 μM $NiCl_2$) in PBS (1% BSA, pH = 7.2, 1 mL) at 0 ºC for 10 minutes. Finally, the cells were transferred to FACS tubes and analyzed by flow cytometry using a 5 laser BD LSR-II SORP. Control samples, incubated without His-RBD, were included to measure background fluorescence. Fluorescence intensity was measured using a 525/50 band-pass filter following excitation by a 488nm laser line. Data were analyzed using FlowJo software (BD Biosciences), and mean fluorescence intensities (MFIs) ± standard deviations from three experiments are shown (Figure S9).

**11. Microscale Thermophoresis (MST).**

Binding of probe **4** to His-tagged RBD. The affinity of probe **4** to His-RBD was measured using a micro-MST Monolith NT.115 instrument (NanoTemper Technologies, Munich, Germany). Probe **4** (100 nM) was incubated with $NiCl_2.6H_2O$ (500 nM) for 30 minutes in PBST buffer (137 mM NaCl, 2.5 mM KCl, 10 mM $Na_2HPO_4$, 2 mM $KH_2PO_4$, pH = 7.4, 0.05% Tween-20). To obtain a sufficient MST signal, a probe concentration above the $K_d$ was used. Accordingly, two-fold serial dilutions of His-RBD ranging from 5 µM to 152 pM were titrated against a constant concentration of probe **4** (100 nM). The resulting protein-probe samples were centrifuged at 21,000 g at 4 °C for 10 minutes to ensure that no precipitate formed. Subsequently, these samples were loaded into Monolith NT.115 MST premium-coated capillaries. MST measurements were conducted at 20% LED power and 20% MST power. The experimental data were analyzed using MO.Affinity Analysis 2.2.7 (NanoTemper Technologies GmbH), and were fitted according to the following equation (equation 1):

$$\frac{[LP]}{[P]} = \frac{([L]+[P]+K_d) - \sqrt{(([L]+[P]+K_d)^2 - 4[L][P])}}{2[P]}$$

where [L] is the probe concentration that is kept constant, [P] is the concentration of the protein, [LP] is the concentration of the bound complex of L and P, and $K_d$ is the dissociation constant. The experiments were performed in triplicate. A similar protocol was used to monitor the binding of the Cy5-appended probe (5 nM, probe **5**) to His-RBD . The resulting binding curve (Figure S10) revealed a dissociation constant ($K_d$) of 20 ± 1 nM.

Binding of His-tagged RBD to ACE2. His-RBD (160 nM) was incubated with probe **4** (100 nM) in the presence of $NiCl_2 \cdot 6H_2O$ (500 nM) for 30 minutes in PBST buffer (137 mM NaCl, 2.5 mM KCl, 10 mM $Na_2HPO_4$, 2 mM $KH_2PO_4$, pH = 7.4, and 0.05% Tween-20). This combination yielded Flu-labeled His-RBD, which was then added to a dilution series of ACE2-Fc that was serially diluted (2.5 µM - 76.3 pM) in PBS in twofold dilutions. The resulting protein-probe samples were centrifuged at 21,000g at 4 °C for 10 minutes. The MST measurements were performed in triplicate as described above and were fitted according to Equation 1.

Binding of probe **5** to His-tagged lectin. MST measurements to assess the binding between probe 5 and His-lectin were carried out in PBST buffer (137 mM NaCl, 2.5 mM KCl, 10 mM $Na_2HPO_4$, 2 mM $KH_2PO_4$, pH 7.4, 0.05% Tween-20), using 1 nM probe **5** and 5 nM $NiCl_2 \cdot 6H_2O$. Two-fold serial dilutions of His-lectin, ranging from 2.5 µM to 152 pM, were titrated against a constant concentration of probe **5** (1 nM). The protein–probe mixtures were centrifuged at 21,000 × g at 4 °C for 10 minutes to remove any precipitate. The samples were then loaded into MST capillaries, and measurements were conducted at 20% LED power and 60% MST power. The experimental data were analyzed according to Equation 1.

**12. Surface plasmon resonance (SPR) measurements**. SPR experiments were conducted using a Biacore S200 instrument (Cytiva). RBD was immobilized on a CM5 chip through amide coupling chemistry, with PBST as the running buffer. The carboxyl groups on the chip were activated with a freshly prepared solution of N-hydroxysuccinimide (50 mM) and 1-ethyl-3-(3-dimethylaminopropyl)carbodiimide (195 mM) in water for 7.5 minutes at a flow rate of 10 µL/min. Subsequently, RBD (10 µM in 150 mM sodium acetate buffer, pH 4.6) was injected for 1 minute (flow rate: 10 µL/min). Residual activated carboxyl groups were blocked by injecting 1 M ethanolamine hydrochloride (pH 8.0) for 5 minutes (flow rate: 10 µL/min). This procedure immobilized approximately 5,700 RU of RBD. To assess binding, an initial concentration of 10 µM of various analogues was used (Figure S8). Upon observing binding responses, the interactions were further analyzed by injecting two-fold serial dilutions (10 µM to 0.156) over a 2-minute period at a flow rate of 50 µL/min, with dissociation monitored for 15 minutes (Figures 5c-left, S7, and S8). The chip was regenerated using 1 mM NaOH. These measurements revealed that, except for HSA-8 (Figure S6) and heparin (Figure S7), the other analogues either did not bind to

RBD or had sensorgrams that could not be fitted (data not shown) to any binding model. The SPR analysis was conducted using using Biacore Insight Evaluation Software. Sensorgrams of HSA-8 were fitted to a 1:1 kinetic binding model, revealing a dissociation constant ($K_d$) of 5.75 μM, with association ($k_{on}$) and dissociation ($k_{off}$) rate constants of $2.73 \times 10^3$ $M^{-1}s^{-1}$ and $1.57 \times 10^{-2}$ $s^{-1}$, respectively. Heparin data were analyzed using a steady-state affinity model, yielding a $K_d$ of 0.43 μM.

**13. Pseudovirus-based infection assays.** Details of the production of pseudoviruses featuring the S glycoprotein of the SARS-CoV-2 Wuhan variant (VSVΔG-S) on a VSV backbone lacking VSV-G (VSVΔG), which expresses a fluorescent reporter (GFP) from the viral genome, were described previously.[5, 6] Briefly, to generate glycoprotein X-complemented pseudoviruses (VSVΔG-X), 1.2 $\times 10^6$ BHK-21 cells were seeded in a 100 mm dish (Greiner, Austria) one day prior to transfection. At 75-80% confluence, the cells were transfected using JetPrime transfection reagent (Polyplus, France) with 1 μg/ml of plasmid encoding the viral glycoprotein. After 24 hours, the cells were infected with VSVΔG-G pseudovirus at a multiplicity of infection (MOI) of 5, with a 1:1000 polybrene infection reagent (Sigma-Aldrich). The infection was carried out for 1 hour at 37°C in a 5% $CO_2$ incubator, with gentle shaking every 15 minutes. Post-infection, the cells were washed six times with Dulbecco's Phosphate-Buffered Saline (DPBS, Biological Industries) to remove residual VSVΔG-G, and the medium was replaced with 5 mL of fresh growth medium. After 30 hours, the cells and the supernatant containing pseudoviruses were harvested and centrifuged at 500g for 10 minutes at 4°C. The supernatant was then collected, supplemented with 30% FBS, aliquoted, and stored at -80°C for further experiments.

To screen for inhibitors, compounds were first pre-plated on a 384-well plate (Greiner) in 1 μM or 10 μM final concentration. However, for the dose-response experiments, the compounds were serially diluted to cover a concentration range from 40 to 0.16 μM. Pseudoviruses were then incubated with α-G neutralizing antibody (1:1000, Absolute antibody, Cat # Ab01402-2.0, RPID: AB_3099672) for 1 h at RT to avoid background from any residual VSVΔG-G activity. Subsequently, 10 μL of the pseudovirus suspension was dispensed into each well. Next, $10^3$ HEK-293T-ACE2-TMPRSS2 cells were added to each well and allowed to settle for 15 minutes at room temperature. The plate was then centrifuged at 1000g for 1 hour at room temperature to enhance the infection efficiency. Following centrifugation, the plate was incubated for 24 hours in a 5%

$CO_2$ incubator at 37 °C. Cells infected with VSVΔG pseudoviruses expressed a GFP fluorescent reporter encoded in the viral genome post-infection. The plates were imaged using the Cell Discoverer 7 (Carl Zeiss, Germany) in widefield mode, equipped with an sCMOS 702 camera (Carl Zeiss). Images were captured using a Plan-APOCHROMAT 5× / 0.35 Autocorr Objective (Carl Zeiss). ZEN blue software 3.1 (Carl Zeiss) was used for image acquisition, with 470 nm excitation to visualize the infected channel. Infected cells were segmented and counted using Cellpose.[7]

The inhibition percentage was calculated as follows:

$$Inhibition\,\% = \left(1 - \frac{I_{compound}}{I_{max}}\right) \times 100$$

where

$I_{Compound}$= the number of infected cells in the presence of compounds

$I_{max}$= the number of infected cells in the absence of compounds

**14. Fluorescence imaging of HeLa cells.** HeLa cells were maintained in DMEM supplemented with 4.5 g/L glucose, 2 mM L-glutamine, 10% (v/v) fetal bovine serum, and 1% penicillin-streptomycin in a 5% $CO_2$ incubator at 37 °C. Cells (30,000 cells/well) were seeded onto glass-bottom culture dishes (35 mm dish, 14 mm microwell, Cat. no: P35G-1.5-14C, MatTek Life Sciences) and allowed to adhere overnight. The following day, the medium was removed, and the cells were washed with PBS. The cells were then incubated with His-lectin (50 µg/mL) in a 37 °C, 5% $CO_2$ incubator for 30 minutes. After incubation, excess His-lectin was gently washed off, and the cells were washed with PBS (containing $Ca^{2+}$ and $Mg^{2+}$). Cells were then incubated with probe 5 (500 nM in PBS) at 37 °C under 5% $CO_2$ for 10 minutes. Afterward, the cells were washed with PBS (containing $Ca^{2+}$ and $Mg^{2+}$) and imaged using an Olympus IX51 fluorescent microscope. Control experiments (without His-lectin or $Ni^{2+}$) were performed under the same conditions. In experiments where lactose was used to inhibit lectin binding to cells, the cells were treated with 100 mM lactose, followed by washing before fluorescent imaging.

**15. Fluorescence assays for detecting lectin binding to HeLa cells.** HeLa cells were cultured in DMEM supplemented with 10% FBS. The next day, the cells were treated with trypsin and centrifuged (1000 rpm, 5 min) to pellet the cells. The supernatant containing trypsin was discarded,

and the cells were resuspended in fresh medium and transferred to Eppendorf tubes. Cells ($6 \times 10^4$) were treated with His-lectin (20 μg/mL), probe 1 (200 nM), and $NiCl_2$ (1 μM) in PBS at room temperature, in the presence or absence of lactose (80 mM) After incubation, the cells were transferred to a 386-well black Corning plate, and fluorescence spectra were recorded.

## 16. Synthesis of probes 1-3: Procedures and schemes

**Compound CP1.** The protected tri-NTA (compound **CP-1**) was synthesized according to the previously reported procedure from our group.[4]

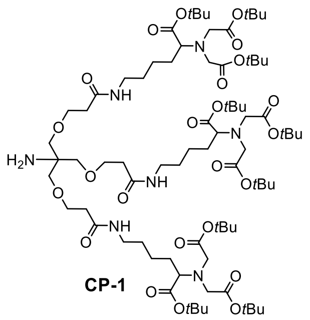

### Synthetic scheme of probe 1

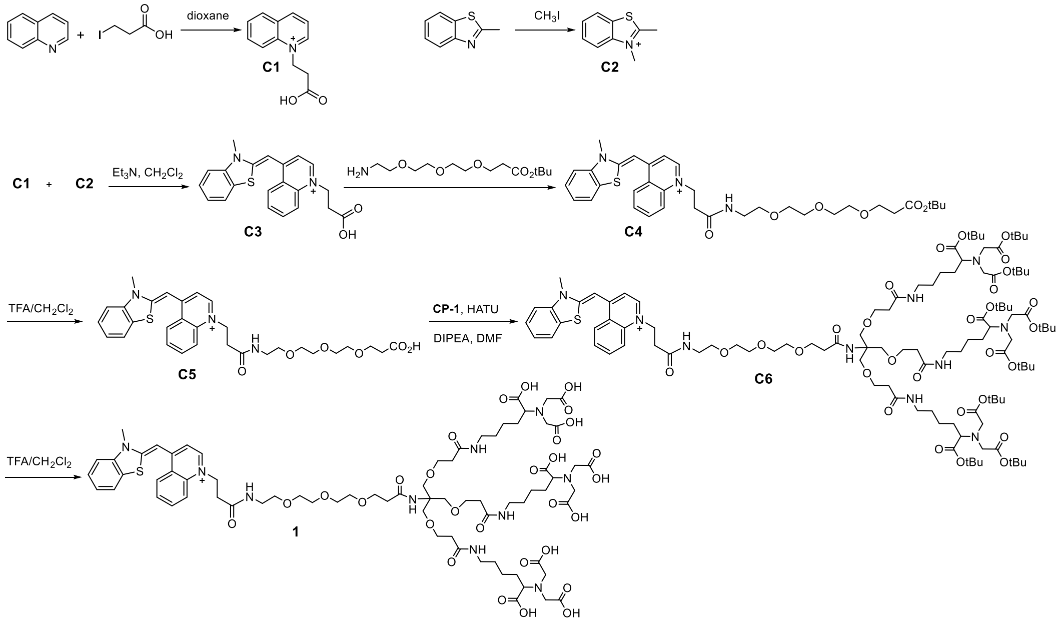

**Synthesis of C1.** 3-iodopropionic acid (5.56 g, 27.9 mmol) was added to a solution of quinoline (2.74 mL, 23.23 mmol) in 30 mL of dioxane. The solution was stirred under reflux for 23h (~100 °C). After cooling to room temperature, the solvent was removed and the precipitate was washed twice with hexane and six times with acetone, yielding a bright yellow solid (5.6 g, 73% yield). $^1$H NMR (500 MHz, DMSO-$d_6$): $\delta$: 9.57 (br. s., 1H), 9.30 (d, $J$ = 7.7 Hz, 1H), 8.63 (d, $J$ = 8.4 Hz, 1H), 8.50 (d, $J$ = 7.3 Hz, 1H), 8.28 (br. s., 1H), 8.19 (br. s., 1H), 8.06 (br. s., 1H), 5.26 (br. s., 2H), 3.09 (br. s., 2H). HRMS m/z calculated for $[M]^+$: 202.0863; found: 202.0847.

**Synthesis of C2.** A solution of 2-methylbenzothiazole (2.98 g, 20 mmol) and iodomethane (11.28 g, 80 mmol) in DMF (10 mL) was mixed and heated under reflux at 60 °C for 24 hours. After refluxing, the DMF was evaporated, and ethyl acetate (100 mL) was added slowly. The resulting precipitate was collected by filtration and washed with ethyl acetate. The solid was then resuspended in ethyl acetate (100 mL) and refluxed for an additional 20 minutes. After the suspension was cooled to room temperature, the product was collected by filtration and washed with ethyl acetate, yielding a bright yellow solid after drying in vacuo (yield: 80.5%). $^1$H NMR (500 MHz, DMSO-$d_6$): $\delta$: 8.44 (d, $J$ = 8 Hz, 1H), 8.29 (d, $J$ = 8 Hz, 1H), 7.90 (br. s., 1H), 7.85-7.72 (m, 1H), 4.20 (br. s., 3H), 3.17 (br. s., 3H). HRMS m/z calculated for $[M]^+$: 164.0528; found: 164.0539.

**Synthesis of C3.** Triethylamine (15.2 mmol, 2.12 mL) was added to a suspension of **C1** (500.3 mg, 1.52 mmol) and **C2** (442.5 mg, 1.52 mmol) in 6 mL dry DCM. A deep red color immediately appeared. The reaction mixture was stirred at room temperature overnight. Then the solvent was evaporated and the product was purified using Combiflash chromatography. $^1$H NMR (400 MHz, Methanol-$d_4$): $\delta$: 8.53-8.46 (m, 1H), 8.42 (d, $J$ = 7.3 Hz, 1H), 8.01-7.95 (m, 1H), 7.86 (ddd, $J$ = 8.4, 7.0, 1.1 Hz, 1H), 7.78-7.73 (m, 1H), 7.64 (ddd, $J$ = 8.4, 7.0, 1.1 Hz, 1H), 7.52-7.44 (m, 2H), 7.34- 7.24 (m, 2 H), 6.70 (s, 1H), 4.73 (t, $J$ = 6.5 Hz, 2H), 3.86 (s, 3H), 2.78 (t, $J$ = 6.6 Hz, 2H). HRMS m/z calculated for $[M]^+$: 363.1162; found: 363.1167.

**Synthesis of C4.** To a solution of **C3** (36.3 mg, 0.1 mmol) in DMF (2 mL) was added HATU (76 mg, 0.2 mmol) and DIPEA (35 µL, 0.2 mmol) and allowed to stir at room temperature for 10 min. Later, tert-butyl 3-(2-(2-(2-aminoethoxy)ethoxy)ethoxy)propanoate (0.12 mmol) was added to the reaction mixture under inert conditions and the reaction was allowed to run overnight. Upon completion of the reaction, DMF was evaporated under vacuum and the crude was purified by RP-HPLC. The purified product was lyophilized to afford an orange compound (15.5 mg, 25%). $^1$H NMR (500 MHz, DMSO-$d_6$): $\delta$: 8.80 (d, $J$ = 8.1 Hz, 1H), 8.53 (d, $J$ = 7.3 Hz, 1H), 8.16-8.08 (m, 2H), 8.06 (d, $J$ = 7.3 Hz, 1H), 8.00 (t, $J$ = 7.4 Hz, 1H), 7.80 (d, $J$ = 8.4 Hz, 1H), 7.76 (t, $J$ = 7.7 Hz, 1H), 7.65-7.59 (m, 1 H), 7.43 (t, $J$ = 7.6 Hz, 1H), 7.35 (d, $J$ = 7.3 Hz, 1H), 7.00 (br. s., 1H), 6.94 (s, 1H), 4.82 (t, $J$ = 6.5 Hz, 2H), 4.03 (s, 3H), 3.55-3.50 (m, 3 H), 3.45-3.35 (m, 18H), 3.31 (t, $J$ = 5.7 Hz, 3H), 3.19-3.12 (m, 4H), 2.76 (t, $J$ = 6.5 Hz, 2H), 2.39-2.35 (m, 2H), 1.37 (s, 9H). $^{13}$C NMR (125 MHz, DMSO-$d_6$) $\delta$ 170.37, 168.99, 160.20, 148.66,145.04, 140.49, 136.89, 133.26, 128.20,

126.72, 125.86, 124.57, 124.19, 123.91, 122.91, 113.06, 118.02, 107.60, 88.20, 79.70, 69.62, 69.58, 69.50, 68.95, 66.17, 50.80, 40.10, 39.93, 39.77, 39.60, 38.59, 35.77, 34.42, 33.84, 27.72. HRMS m/z calculated for $[M]^+$: 622.2945; found: 622.2953.

**Synthesis of C5.** The tert-butyl ester group was deprotected by adding TFA (100 µL) to a solution of **C4** (15 mg, 24.0 µmol) in DCM (1 mL) at 0 °C. The reaction mixture was warmed up to the room temperature and stirring was continued for another 3.5 h. After the reaction was completed, DCM and TFA were evaporated. The traces of TFA were removed by co-evaporation with DCM. The crude compound was washed three times with cold diethyl ether, dissolved in 3 mL of ACN/$H_2O$ (1:1), and lyophilized under high vacuum to yield an orange powder as the product. (9.1 mg, 67%).$^1$H NMR (400 MHz, DMSO-$d_6$): $\delta$: 8.79 (d, $J$ = 8.1 Hz, 1 H), 8.52 (d, $J$ = 7.3 Hz, 1 H), 8.15-8.08 (m, 2 H), 8.05 (d, $J$ = 7.5 Hz, 1 H), 8.00 (t, $J$ = 7.8 Hz, 1 H), 7.79 (d, $J$ = 8.4 Hz, 1 H), 7.75 (t, $J$ = 7.7 Hz, 1 H), 7.62 (t, $J$ = 7.8 Hz, 1 H), 7.43 (t, $J$ = 7.6 Hz, 1 H), 7.34 (d, $J$ = 7.3 Hz, 1 H), 7.04 (br. s., 1 H), 6.93 (s, 1 H), 4.81 (t, $J$ = 6.4 Hz, 2 H), 4.02 (s, 4 H), 3.54 (t, $J$ = 6.4 Hz, 2 H), 3.44-3.37 (m, 8 H), 3.34-3.28 (m, 2 H), 3.16 (q, $J$ = 5.6 Hz, 2 H), 2.76 (t, $J$ = 6.4 Hz, 2 H), 2.40 (t, $J$ = 6.4 Hz, 2 H). $^{13}$C NMR (100 MHz, DMSO-$d_6$): $\delta$ 172.59, 169.00, 160.18, 148.64, 145.02, 140.47, 136.88, 133.25, 128.19, 126.72, 125.85, 124.56, 124.18, 123.91, 122.89, 118.01, 113.05, 107.60, 88.20, 69.62, 69.54, 69.49, 68.95, 66.18, 50.80, 38.61, 34.69, 34.42, 33.83. HRMS m/z calculated for $[M]^+$: 566.2319; found: 566.2333.

**Synthesis of C6.** To a solution of **C5** (6 mg, 0.01 mmol) in dry $CH_2Cl_2$ (2 mL), DIPEA (4 µL, 0.02 mmol) and HATU (8 mg, 0.02 mmol) were added sequentially, and the reaction mixture was stirred for 15 minutes. Compound **CP-1** (16.0 mg, 0.01 mmol) dissolved in $CH_2Cl_2$ (1 mL) was then added dropwise, and the reaction mixture was stirred at room temperature for 12 hours under argon and in the dark. After the starting materials were consumed (monitored by TLC), $CH_2Cl_2$ was removed under vacuum, and the residue was purified by RP-HPLC to afford **C6** (11 mg, 52% yield) as an orange viscous liquid. $^1$H NMR (400 MHz, DMSO-$d_6$): $\delta$: 8.80 (d, $J$ = 8.6 Hz, 1H), 8.54 (d, $J$ = 7.3 Hz, 1H), 8.14 (d, $J$ = 8.6 Hz, 1H), 8.10 (t, $J$ = 5.5 Hz, 1H), 8.06 (d, $J$ = 7.8 Hz, 1H), 8.00 (t, $J$ = 7.8 Hz, 1H), 7.88-7.73 (m, 5H), 7.63 (t, $J$ = 7.8 Hz, 1H), 7.47-7.40 (m, 1H), 7.36 (d, $J$ = 7.3 Hz, 1H), 7.09 (s, 1H), 6.95 (s, 1H), 4.82 (t, $J$ = 6.3 Hz, 2H), 4.03 (s, 2H), 3.57-3.46 (m, 12H), 3.43-3.38 (br. s, 30H), 3.37 (s, 3H), 3.34-3.27 (m, 3H), 3.22 (t, $J$ = 7.5 Hz, 3H), 3.16 (q, $J$ = 5.4 Hz, 1H), 3.07-2.93 (m, 6H), 2.77 (t, $J$ = 6.4 Hz, 1H), 2.34-2.19 (m, 7H), 1.58-1.44 (m, 6H),

1.39 (br. s, 81H), 1.25-1.15 (m, 3H). $^{13}$C NMR (125 MHz, DMSO-$d_6$): $\delta$ 171.5, 170.0, 169.8, 169.0, 160.2, 158.3, 158.0, 148.7, 145.0, 140.5, 136.9, 133.3, 128.2, 126.7, 125.9, 124.6, 124.2, 123.9, 122.9, 118.0, 113.1, 107.6, 88.2, 80.3, 79.9, 69.6, 69.0, 69.5, 67.3, 66.8, 64.5, 59.6, 53.2, 50.8, 36.5, 35.9, 35.5, 34.4, 33.8, 29.7, 28.9, 27.7, 22.9. HRMS m/z calculated for $[M]^+$: 2122.2398; found: 2122.2403.

**Synthesis of probe 1.** To compound **C6** (11 mg, 0.005 mmol), a 20% v/v solution of TFA in $CH_2Cl_2$ was added at 0 ºC. The orange-colored solution immediately decolorized, and the reaction was stirred at room temperature for 4-5 hours in the dark. Upon complete consumption of **C6**, as monitored by TLC and LCMS, the reaction mixture was evaporated under reduced pressure, restoring the orange color. Residual TFA was removed by repeated co-evaporation with $CH_2Cl_2$. The crude product was washed three times with cold diethyl ether, and the supernatant was decanted. After additional washes with cold diethyl ether, the compound was dissolved in a 50% v/v ACN/water mixture (1 mL) and lyophilized to yield a bright orange-colored product (5.5 mg, 68% yield). $^1$H NMR (400 MHz, DMSO-$d_6$): $\delta$: 8.80 (d, $J$ = 8.8 Hz, 1H), 8.53 (d, $J$ = 7.3 Hz, 1H), 8.17-8.09 (m, 2H), 8.05 (d, $J$ = 7.7 Hz, 1H), 8.00 (t, $J$ = 7.8 Hz, 1H), 7.84-7.76 (m, 4H), 7.62 (t, $J$ = 7.8 Hz, 1H), 7.43 (t, $J$ = 7.6 Hz, 1H), 7.36 (d, $J$ = 7.3 Hz, 1H), 7.26 (s, 1H), 7.14 (s, 1H), 7.10 (br. s., 1H), 7.01 (s, 1H), 6.94 (s, 1H), 4.82 (t, $J$ = 6.4 Hz, 2H), 4.03 (s, 3H), 3.60-3.42 (m, 30H), 3.36-3.27 (m, 6H), 3.20-3.13 (m, 2H), 3.00 (d, $J$ = 5.5 Hz, 7H), 2.77 (t, $J$ = 6.3 Hz, 2H), 2.33-2.22 (m, 10H), 1.61-1.41 (m, 6H), 1.39-1.29 (m, 12H). $^{13}$C NMR (125 MHz, DMSO-$d_6$): $\delta$ 173.9, 173.1, 170.3, 169.9, 169.0, 161.0, 160.2, 148.7, 145.0, 140.5, 136.9, 133.3, 128.2, 126.7, 125.9, 124.6, 124.2, 123.9, 122.9, 118.0, 117.3, 117.1, 115.0, 113.1, 107.6, 88.2, 69.6, 69.5, 69.4, 69.0, 68.2, 67.4, 66.8, 64.2, 59.6, 53.3, 50.8, 40.1, 40.0, 39.9, 39.8, 39.7, 39.6, 39.3, 39.2, 39.0, 38.6, 38.4, 36.5, 35.9, 34.4, 33.9, 29.2, 28.8, 28.7, 23.1. HRMS m/z calculated for $[M]^+$: 1617.6764; found: 1617.6777.

**Synthetic scheme of probe 2**

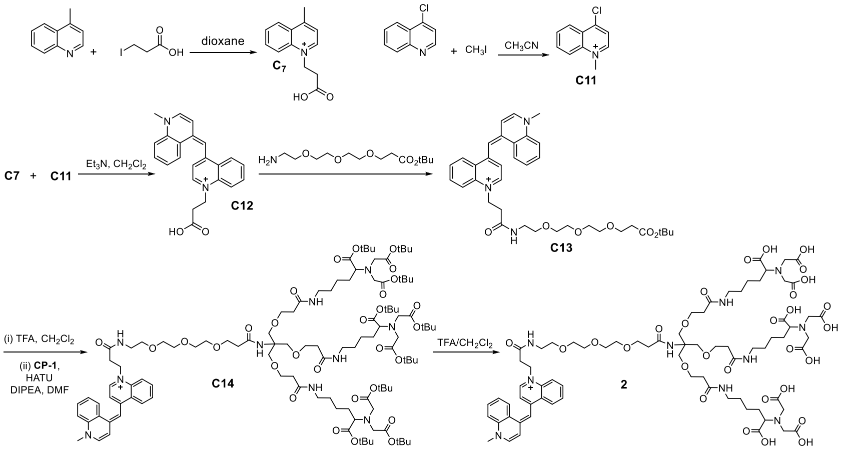

**Synthesis of C11.** To 4-chloroquinoline (2.6 mL, 16 mmol) was added methyl iodide (2 mL, 32 mmol) in acetonitrile. The reaction mixture was stirred at 55 ºC for 24 hours. Upon completion, as monitored by TLC, the solvent was evaporated under reduced pressure. The resulting yellow solid was repeatedly washed with hexane, yielding 3.3 g of product (67.5%). $^1$H NMR (300 MHz, DMSO-$d_6$): $\delta$ 9.14-9.06 (m, 1H), 8.87 (dd, $J$ = 5.7, 2.4 Hz, 1H), 8.48 (d, $J$ = 8.8 Hz, 1H), 8.41 (d, $J$ = 8.4 Hz, 1H), 8.37-8.27 (m, 1H), 8.18-8.06 (m, 1H), 4.54 (s, 3H), HRMS m/z calculated for $[M]^+$: 178.0418; found: 178.0420.

**Synthesis of C12.** Triethylamine (2.08 mL, 15.0 mmol) was added to a mixture of **C7** (510 mg, 1.5 mmol) and **C11** (460 mg, 1.5 mmol) in dry DCM (5 mL), after which the solution turned blue. The reaction mixture was stirred at room temperature overnight. Afterward, the solvent was evaporated, and the product was purified using Combiflash chromatography to afford compound **C12** (410 mg, yield: 56%).$^{1H\ NMR}$ (300 MHz, DMSO-$d_6$): $\delta$: 8.66 (dd, $J$ = 8.6, 2.2 Hz, 2H), 8.35 (d, $J$ = 7.0 Hz, 1H), 8.12 (d, $J$ = 7.3 Hz, 1H), 8.03-7.95 (m, 1H), 7.93-7.84 (m, 3H), 7.66-7.54 (m, 4H), 7.21 (s, 1H), 4.62 (br. s., 2H), 2.44 (br. s., 2H). HRMS m/z calculated for $[M]^+$: 357.1598; found: 357.1603.

**Synthesis of C13.** To a solution of **C12** (48.4 mg, 0.1 mmol) in DMF (2 mL), HATU (76 mg, 0.2 mmol) and DIPEA (35 µL, 0.2 mmol) were added and allowed to stir at room temperature for 10 minutes. Later, tert-butyl 3-(2-(2-(2-aminoethoxy)ethoxy)ethoxy)propanoate (32 mg, 0.12 mmol) was added to the reaction mixture under inert conditions, and the reaction was allowed to proceed

overnight. Upon completion, DMF was evaporated under vacuum, and the crude product was purified by RP-HPLC using an ACN/$H_2O$ system. The purified product was lyophilized to afford a blue compound (20 mg, 27%). $^1$H NMR (400 MHz, DMSO-$d_6$): $\delta$: 9.39 (d, $J$ = 6.4 Hz, 1H), 8.93 (d, $J$ = 4.6 Hz, 1H), 8.76 (d, $J$ = 8.7 Hz, 1H), 8.54 (d, $J$ = 8.7 Hz, 1H), 8.31-8.21 (m, 2H), 8.12-8.05 (m, 2H), 8.00 (d, $J$ = 6.2 Hz, 1H), 7.87 (t, $J$ = 5.6 Hz, 1H), 7.80 (t, $J$ = 7.2 Hz, 1H), 7.65 (t, $J$ = 7.2 Hz, 1H), 7.52 (d, $J$ = 4.6 Hz, 1H), 6.11 (t, $J$ = 7.2 Hz, 1H), 3.54 (t, $J$ = 6.3 Hz, 2H), 4.59 (s, 3H), 3.45-3.40 (m, 8H), 3.34-3.29 (m, 2H), 3.19-3.11 (m, 2H), 2.55 (d, $J$ = 5.5 Hz, 2H), 2.39 (t, $J$ = 6.2 Hz, 2H), 2.34-2.27 (m, 2H), 1.40-1.35 (m, 9H). $^{13}$C NMR (100 MHz, DMSO-$d_6$): $\delta$ 171.3, 170.4, 160.5, 150.3, 149.7, 147.8, 146.7, 138.4, 135.0, 130.4, 129.8, 127.6, 126.3, 125.4, 123.2, 121.0, 120.6, 120.2, 79.7, 69.7, 69.6, 69.5, 69.0, 66.2, 45.3, 38.5, 35.8, 32.6, 30.1, 27.7. HRMS m/z calculated for $[M]^+$: 616.3381; found: 616.3367.

**Synthesis of C14.** To a solution of C**13** (10 mg, 0.015 mmol) in dry $CH_2Cl_2$ (2 mL), TFA (200 µL, 10% v/v of TFA/$CH_2Cl_2$) was added and the mixture was stirred at room temperature in the dark until the reaction was complete, as monitored by LCMS. Upon completion, the reaction mixture was evaporated under vacuum and resuspended in ACN/water (1 mL, 50% v/v of ACN/water), then frozen under liquid nitrogen and lyophilized to yield a bluish powder. The crude powder was resuspended in $CH_2Cl_2$, and DIPEA (6 µL, 0.03 mmol) and HATU (12 mg, 0.03 mmol) were added sequentially, followed by stirring the reaction mixture for 15 minutes. Subsequently, compound **CP-1** (24.0 mg, 0.015 mmol), dissolved in $CH_2Cl_2$ (1 mL), was added dropwise, and the mixture was stirred at room temperature for 12 hours under argon and dark conditions. After the starting materials were consumed (as monitored by LCMS), $CH_2Cl_2$ was removed under vacuum, and the residue was purified by RP-HPLC to afford **C14** (13 mg, 41% yield) as a blue-colored sticky gum. $^1$H NMR (500 MHz, METHANOL-$d_4$): $\delta$: 9.34 (d, $J$ = 6.2 Hz, 1H), 9.32 (d, $J$ = 6.1 Hz, 1H), 8.69-8.58 (m, 3H), 8.72 (d, $J$ = 8.9 Hz, 1H), 8.13 (t, $J$ = 7.7 Hz, 2H), 8.45-8.33 (m, 2H), 7.77 (d, $J$ = 6.1 Hz, 1H), 7.70 (d, $J$ = 6.1 Hz, 1H), 5.42 (t, $J$ = 6.2 Hz, 2H), 3.67 (br. s., 16H), 4.75 (s, 2H), 3.46 (t, $J$ = 5.4 Hz, 2H), 3.63-3.5 (m, 30H), 3.41 (t, $J$ = 7.4 Hz, 4 H), 3.17-3.22 (m, 6H), 3.11 (t, $J$ = 6.2 Hz, 2H), 2.44-2.42 (m, 6H), 1.73-1.66 (m, 6H), 1.59-1.54 (m, 6H), 1.50 (br. s., 27H), 1.49 (s, 56H), 1.45-1.35 (m, 6H). HRMS m/z calculated for $[M]^+$: 2116.2834; found: 2116.2835.

**Synthesis of probe 2.** To compound **C13** (13 mg, 0.006 mmol), TFA/$CH_2Cl_2$ (10% v/v) was added at 0 °C, resulting in the immediate decolorization of the blue-colored solution. The reaction was stirred at room temperature in the dark for 8 hours. Complete consumption of **C11** was monitored by TLC and LCMS. The reaction mixture was then evaporated under reduced pressure, restoring the blue color. Residual TFA was removed by repeated co-evaporation with $CH_2Cl_2$. The crude product was washed three times with cold diethyl ether and the supernatant was decanted. After further washing with cold diethyl ether, the compound was dissolved in a 1 mL ACN/water mixture (50% v/v) and lyophilized to yield a blue-colored product (7 mg, 70% yield). $^1$H NMR (500 MHz, DMSO-$d_6$): $\delta$: 8.70 (t, $J$ = 8.1 Hz, 2H), 8.26 (d, $J$ = 7.2 Hz, 1H), 8.14- 8.03 (m, 2H), 8.00-7.92 (m, 2H), 7.80 (d, $J$ = 4.8 Hz, 3H), 7.72-7.56 (m, 3H), 7.26 (s, 1H), 7.10 (br. s., 1H), 4.72-4.65 (m, 2H), 4.09 (s, 3H), 3.58-3.39 (m, 35H), 3.34 (d, $J$ = 6.2 Hz, 4H), 3.18 (d, $J$ = 5.4 Hz, 3H), 3.07-2.93 (m, 6H), 2.20- 2.38 (m, 6H), 1.59-1.53 (m, 4H), 1.40 (br. s., 12H), 1.30-1.22 (m, 6H). HRMS m/z calculated for $[M]^+$: 1611.7200; found: 1611.7207.

**Synthetic scheme of probe 3**

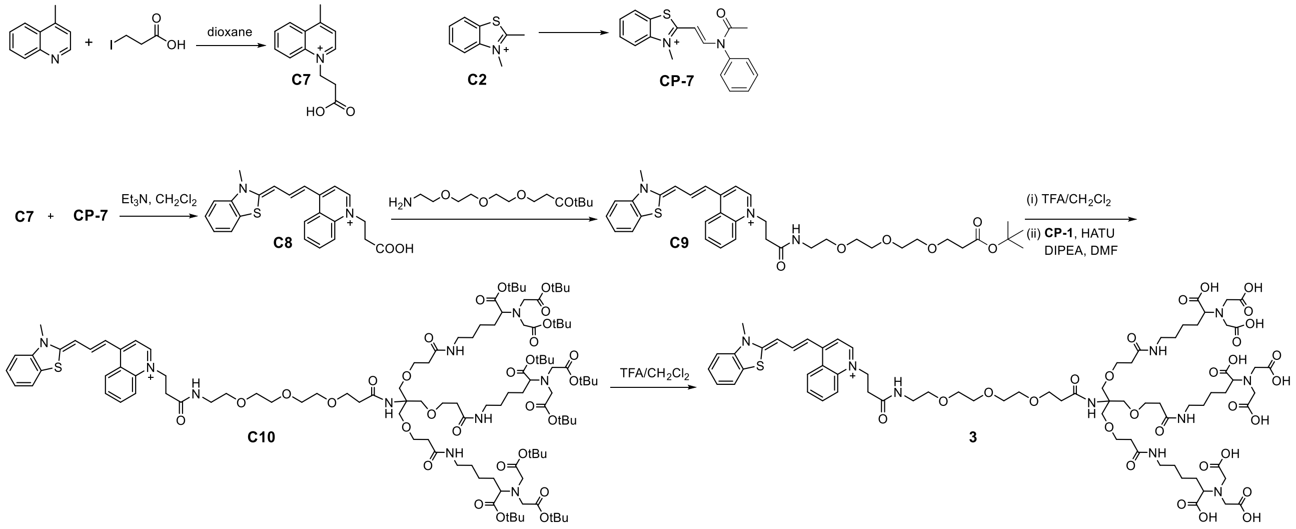


**Synthesis of C7.** 3-iodopropionic acid (4.0 g, 20.0 mmol) was added to a solution of 4-methylquinoline (2.4 mL, 16.7 mmol) in 30 mL of dioxane. The solution was stirred under reflux (~100 °C) for 24 hours. After cooling to room temperature, the solvent was removed, and the precipitate was washed twice with hexane and six times with acetone, yielding a bright yellow solid (3.9 g, 68% yield). $^1$H NMR (300 MHz, DMSO-$d_6$): $\delta$ 6.84 (d, $J$ = 6.1 Hz, 1H), 6.19 (d, $J$ = 8.6 Hz, 1H), 6.07 (d, $J$ = 9.2 Hz, 1H), 5.92 (t, $J$ = 8.0 Hz, 1H), 5.70 (t, $J$ = 7.7 Hz, 1H), 5.57 (d, $J$

= 6.1 Hz, 1H), 2.95 (t, *J* = 6.5 Hz, 2H), 0.90 (t, *J* = 6.5 Hz, 2H), 0.71 (s, 3H). HRMS-ESI (m/z): calculated for $[M]^+$: 216.1019; found: 216.1024.

**Compound CP7**. 2,3-Dimethylbenzothiazolium iodide (**C2**, 500 mg, 1.72 mmol) and N,N′-diphenylformamidine (676.4 mg, 3.45 mmol) were suspended in acetic anhydride (6 mL). The reaction mixture was stirred under reflux for 2 hours. After cooling to room temperature, ethyl acetate (30 mL) was added with stirring. The resulting precipitate was filtered, washed twice with diethyl ether and twice with ethyl acetate, and then dried under vacuum to yield a dark red powder (535.5 mg, 71.3%). $^1$H NMR (500 MHz, DMSO-$d_6$): $\delta$: 8.80 (d, *J* = 13.9 Hz, 1H), 8.32 (d, *J* = 8.0 Hz, 1H), 8.10 (d, *J* = 8.4 Hz, 1H), 7.82-7.76 (m, 1H), 7.74-7.66 (m, 3H), 7.65 (d, *J* = 7.2 Hz, 1H), 7.54 (d, *J* = 7.2 Hz, 2H), 5.70 (d, *J* = 13.9 Hz, 1H), 3.32 (s, 2H), 2.06 (s, 3H).$^{13}$C NMR (125 MHz, DMSO-$d_6$): $\delta$: 171.9, 170.0, 145.0, 141.7, 136.9, 130.6, 130.1, 129.0, 128.3, 127.9, 126.5, 124.0, 116.2, 96.4, 35.6, 23.1. MS-ESI (m/z): calculated for $[M-I]^+$: 309.11; found: 309.27.

**Synthesis of C8.** Triethylamine (1.5 mL, 10.8 mmol) was added to a mixture of **C2** (290 mg, 1.0 mmol) and **C7** (340 mg, 1.0 mmol) in 5 mL of dry DCM. The solution changed color to dark blue, and the reaction mixture was stirred at room temperature overnight. Afterward, the solvent was evaporated, and the product was purified using Combiflash chromatography (235 mg, 45% yield). $^1$H NMR (300 MHz, DMSO-$d_6$): $\delta$: 8.73-8.63 (m, 2H), 8.34 (d, *J* = 7.2 Hz, 1H), 8.12 (d, *J* = 7.3 Hz, 1H), 8.06-7.96 (m, 2H), 7.95-7.83 (m, 2H), 7.69-7.55 (m, 4H), 7.22 (s, 1H), 4.02 (s, 3H), 4.62 (t, *J* = 6.6 Hz, 2H), 2.44 (t, *J* = 6.6 Hz, 1H). HRMS m/z calculated for $[M]^+$: 389.1318; found: 389.1322.

**Synthesis of C9.** To a solution of **C8** (103 mg, 0.2 mmol) in DMF (2 mL), HATU (152 mg, 0.4 mmol) and DIPEA (70 µL, 0.4 mmol) were added, and the mixture was stirred at room temperature for 10 minutes. Then, tert-butyl 3-(2-(2-(2-aminoethoxy)ethoxy)ethoxy)propanoate (64 mg, 0.24 mmol) was added under inert conditions, and the reaction was allowed to proceed overnight. Upon completion, DMF was evaporated under vacuum, and the crude product was purified by RP-HPLC using an ACN/$H_2O$ system. The purified product was lyophilized to afford a dark blue compound (38 mg, 24.5% yield $^{1H\ NMR}$ (400 MHz, DMSO-$d_6$): $\delta$: 8.46 (d, *J* = 8.4 Hz, 1H), 8.27 (d, *J* = 7.3 Hz, 1H), 8.16 (t, *J* = 12.8 Hz, 1H), 8.12-8.03 (m, 2H), 7.95 (t, *J* = 7.7 Hz, 1H), 7.90 (d, *J* = 7.7 Hz, 1H), 7.81 (d, *J* = 7.3 Hz, 1H), 7.70 (t, *J* = 7.6 Hz, 1H), 7.61 (d, *J* = 8.1 Hz, 1H), 7.50 (t, *J*=7.7 Hz, 1H), 7.32 (t, *J* = 7.6 Hz, 1H), 7.09 (d, *J* = 13.2 Hz, 1H), 6.51 (d, *J* = 12.3 Hz, 1H), 4.76 (t, *J* = 6.3

Hz, 2H), 4.10 (q, $J$ = 5.3 Hz, 1H), 3.75 (s, 3H), 3.54 (t, $J$ = 6.3 Hz, 2H), 3.47-3.38 (m, 8H), 3.16 (dd, $J$ = 5.3, 2.6 Hz, 3H), 2.73 (t, $J$ = 6.3 Hz, 2H), 2.37 (t, $J$ = 6.3 Hz, 2H), 1.36 (s, 9H). $^{13}$C NMR (100 MHz, DMSO-$d_6$): $\delta$ 170.4, 169.0, 162.0, 150.4, 144.0, 142.9, 142.0, 137.7, 133.3, 127.7, 126.6, 125.2, 124.7, 124.3, 124.2, 122.6, 112.6, 117.8, 109.2, 99.1, 79.7, 69.7, 69.6, 69.5, 69.0, 66.2, 50.5, 48.6, 38.6, 35.8, 34.5, 32.9, 27.7. HRMS m/z calculated for $[M]^+$: 648.3102; found: 648.3103.

**Synthesis of C10.** To a solution of **C9** (10 mg, 0.015 mmol) in dry $CH_2Cl_2$(2 mL), TFA (200 µL, 10% v/v in $CH_2Cl_2$) was added, and the mixture was stirred at room temperature in the dark until the reaction was complete. Then, the reaction mixture was evaporated under vacuum, resuspended in ACN/water (1 mL, 50% v/v), and lyophilized to a dark bluish powder. The crude powder was then resuspended in $CH_2Cl_2$ (1 mL), and then DIPEA (6 µL, 0.03 mmol) and HATU (12 mg, 0.03 mmol) were sequentially added. The reaction mixture was stirred for 15 minutes, after which compound **CP-1** (24.0 mg, 0.015 mmol) dissolved in $CH_2Cl_2$ (1 mL) was added dropwise. The reaction was stirred at room temperature for 12 hours under argon and in the dark. After consumption of the starting materials, as monitored by LCMS, $CH_2Cl_2$ was removed under vacuum, and the residue was purified by RP-HPLC to afford **C10** (8 mg, 25% yield) as a blue-colored sticky gum. $^1$H NMR (400 MHz, ACN-$d_3$): $\delta$ 8.38 (d, $J$ = 8.4 Hz, 1H), 8.02-8.18 (m, 2H), 7.97- 7.86 (m, 2H), 7.74 (d, $J$ = 7.6 Hz, 1H), 7.71-7.62 (m, 1H), 7.57 (d, $J$ = 7.3 Hz, 1H), 7.54-7.46 (m, 1H), 7.44-7.38 (m, 1H), 7.32 (t, $J$ = 7.6 Hz, 1H), 6.97 (d, $J$ = 13.4 Hz, 2H), 6.76 (t, $J$ = 5.4 Hz, 2H), 6.38 (d, $J$ = 12.3 Hz, 1H), 6.63 (s, 1H), 4.73 (t, $J$ = 6.2 Hz, 2H), 3.69 (s, 3H), 3.52-3.63 (m, 15H), 3.52-3.40 (m, 15H), 3.40-3.33 (m, 6H), 3.33-3.21 (m, 6H), 3.11 (q, $J$ = 6.5 Hz, 6H), 2.96 (br. s., 10H), 2.80 (t, $J$ = 6.2 Hz, 2H), 2.36-2.26 (m, 6H), 1.68-1.52 (m, 6H), 1.50-1.39 (d, 81H), 1.36-1.24 (m, 3H).$^{13}$C NMR (100 MHz, ACN-$d_3$):$\delta$ 173.0, 172.0, 171.5, 170.4, 164.2, 145.3, 143.8, 139.2, 134.5, 128.8, 127.7, 126.5, 125.8, 125.5, 123.4, 113.4, 110.1, 109.9, 82.0, 81.5, 71.1, 71.0, 70.4, 70.0, 68.5, 66.2, 60.9, 54.6, 52.0, 39.9, 38.0, 37.3, 35.6, 33.9, 30.8, 30.0, 28.4, 24.2. HRMS m/z calculated for $[M]^+$: 2148.2555; found: 2148.2549.

**Synthesis of probe 3.** To compound C**14** (8 mg, 0.0037 mmol), a 10% v/v solution of TFA in $CH_2Cl_2$ was added at 0 ºC, which immediately decolorized the blue solution. The reaction was stirred at room temperature for 12 hours in the dark. Upon complete consumption of C**14**, as monitored by TLC and LCMS, the reaction mixture was evaporated under reduced pressure,

restoring the blue color. Residual TFA was removed by repeated co-evaporation with $CH_2Cl_2$.The crude product was washed three times with cold diethyl ether, and the supernatant was decanted. After additional washes with cold diethyl ether, the compound was dissolved in a 50% v/v ACN/water mixture (1 mL) and lyophilized to obtain a deep, blue-colored product (3.1 mg, 51% yield). $^{1H\ NMR}$ (500 MHz, DMSO-$d_6$): $\delta$: 8.81 (d, $J$ = 8.4 Hz, 1H), 8.57-8.48 (m, 1H), 8.15 (d, $J$ = 9.1 Hz, 1H), 8.07 (dd, $J$ = 7.4, 3.9 Hz, 1H), 7.89-7.78 (m, 4H), 7.76 (t, $J$ = 7.8 Hz, 1H), 7.63 (t, $J$ = 7.7 Hz, 1H), 7.44 (t, $J$ = 7.5 Hz, 1H), 7.37 (d, $J$ = 7.2 Hz, 1H), 6.96 (s, 1H), 4.83 (t, $J$ = 5.1 Hz, 2H), 4.04 (s, 3H), 3.53 (d, $J$ = 9.5 Hz, 18H), 3.48-3.39 (m, 14H), 3.21-3.12 (m, 2H), 3.05-2.96 (m, 4H), 2.78 (br. s., 2H), 2.34-2.22 (m, 12H), 1.56-1.67 (m, 3H), 1.55-1.44 (m, 6H), 1.44-1.33 (m, 14H), HRMS m/z calculated for $[M]^+$: 1643.6921; found: 1643.6929.

## 17. Synthesis of HSA analogs: Procedures and schemes

The chemical structures of heparan sulfate analogs (HSA 1-10)

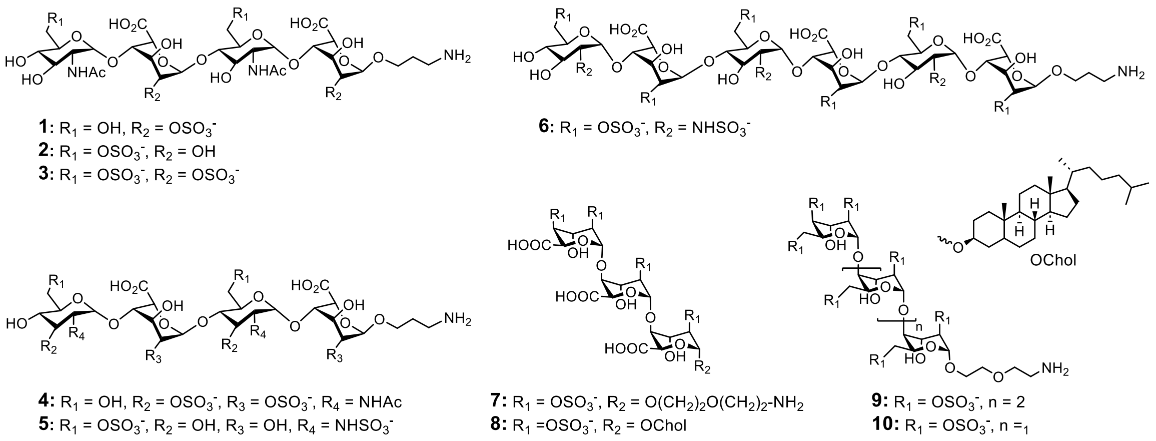


The synthesis of compounds **1–4**, **7–8**, and **10** was previously reported in the literature.[8-10]The building blocks for tetrasaccharides **11** and **15**, as well as disaccharide **16**,[11] were synthesized using previously published procedures.

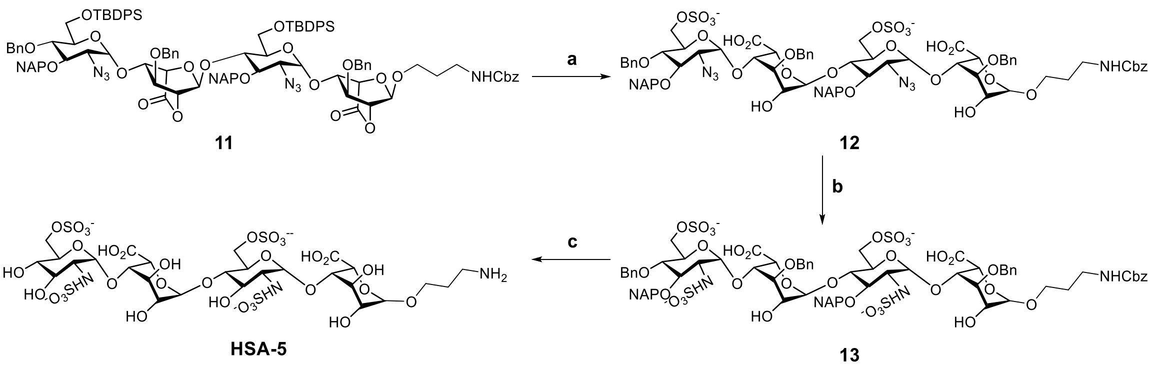


**Synthesis of HSA-5. a:** (1) HF.Py/ Py 0˚C,(2) $SO_3$.$NMe_3$, DMF, 60˚C; (3) LiOH.$H_2O$, $H_2O$:THF,(1:1). **b:** 1M $PMe_3$.THF, 0.1M $NaOH_{aq}$, $SO_3$.Py, MeOH. **c**: $Pd(OH)_2$/C/$H_2$ , $H_2O$.

***N*-benzyloxycarbonyl-3-aminopropyl-*O*-[(2-azido-4-benzyl-2-deoxy-3-*O*-naphthylmethy-6-*O*-sulfonato-*α*-D-gluglucopyranosyl)-(1→4)-*O*-(3-*O*-benzyl-*α*-L-idopyranosyluronate)-(1→4)-*O*-[(2-azido-3-*O*-naphthylmethy-6-*O*-sulfonato-2-deoxy-*α*-D-gluglucopyranosyl)]-(1→4)-*O*-(3-*O*-benzyl-*α*-L-idopyranosiduronate (12).** Compound **11** (1 mmol) was dissolved in 5 mL of 70% HF·Py (hydrogen fluoride-pyridine complex) in pyridine and stirred at room

temperature (RT) for 12 hours. Afterward, the solvent was removed, and the residue was dissolved in 2 mL of DMF (N,N-dimethylformamide). To this solution, the $SO_3 \cdot NMe_3$ complex (10 mmol per hydroxyl group, 10 equivalents) was added under a nitrogen ($N_2$) atmosphere. The mixture was stirred at 70°C for 3 days. Upon completion of the reaction, the mixture was concentrated under reduced pressure, and the resulting product was purified by silica gel column chromatography using a mixture of methanol/dichloromethane (MeOH/DCM, 1:10 v/v), the solvent was removed and the compound was dissolved in THF:$H_2O$ (1:1) followed by addition of LiOH.$H_2O$ (10 eq) and kept it on stirring for 2 h at room temperature. After 2 hours, the reaction was quenched using DOWEX 50WX2 $H^+$ resin. The reaction mixture was evaporated and purified by reverse-phase C18 column chromatography (MeOH/$H_2O$, 3:2 v/v), followed by evaporation and drying under vacuum, yielding compound **12** in 67% over three steps. $^1$H NMR (400 MHz, Methanol-$d_4$) $\delta$: 7.79 (dt, $J$ = 20.1, 6.5 Hz, 8H), 7.44 (dddd, $J$ = 12.3, 6.8, 5.0, 2.6 Hz, 6H), 7.38-7.20 (m, 22H), 5.40 (s, 1H), 5.15-5.01 (m, 5H), 4.99-4.93 (m, 2H), 4.78-4.72 (m, 2H), 4.64 (d, $J$ = 12.7 Hz, 4H), 4.55 (s, 1H), 4.48-4.42 (m, 1H), 4.37-4.26 (m, 5H), 4.22 (d, $J$ = 9.8 Hz, 1H), 4.19-4.13 (m, 1H), 4.06 (dd, $J$ = 13.7, 9.9 Hz, 1H), 4.02-3.91 (m, H), 3.90-3.71 (m, 4H), 3.70-3.61 (m, 3H), 3.54 (dd, $J$ = 10.1, 5.0 Hz, 2H), 3.23 (ddd, $J$ = 25.5, 12.5, 6.0 Hz, 2H), 1.83-1.75 (m, 2H). $^{13}$C NMR (101 MHz, Methanol-$d_4$) δ 175.13, 174.36, 157.45, 138.08, 133.46, 133.12, 128.15, 128.09, 128.05, 127.95, 127.85, 127.69, 127.63, 127.53, 127.40, 127.25, 126.76, 126.19, 126.02, 125.80, 125.52, 125.41, 101.23, 95.41, 94.95, 80.98, 79.07, 73.32, 72.77, 72.38, 72.05, 71.34, 70.43, 70.23, 69.96, 68.50, 68.07, 67.16, 66.30, 66.12, 65.96, 65.68, 64.55, 47.71, 47.50, 47.29, 47.07, 38.62, 29.42, 28.56. HRMS m/z calculated for $C_{78}H_{81}N_7O_{29}S_2^{-2}$ 821.7266; found 821.7269.

***N*-benzyloxycarbonyl-3-aminopropyl-*O*-[(2-amino-4-benzyl-2-deoxy-3-*O*-naphthylmethy-6-*O*-sulfonato-$\alpha$-D-gluglucopyranosyl)-(1→4)-*O*-(3-*O*-benzyl-$\alpha$-L-idopyranosyluronate)-(1→4)-*O*-[(2-amino-3-*O*- naphthylmethy-6-*O*-sulfonato-2-deoxy-$\alpha$-D-gluglucopyranosyl)]-(1→4)-*O*-(3-*O*-benzyl-$\alpha$-L-idopyranosiduronate (13).** A solution of compound **12** in THF (3 mL) was prepared, and 1M $PMe_3$ in THF (8 equivalents with respect to azide group) along with 0.1M aqueous NaOH (10 equivalents) were added. The mixture was stirred at room temperature for 12 hours. Afterward, the reaction was quenched with glacial acetic acid, and the solvent was evaporated. The resulting product was purified using reverse-phase C18 column chromatography (MeOH/$H_2O$, 2:3 v/v), followed by passage through a DOWEX 50WX8 $Na^+$ resin column.

Subsequently, a solution of the amine derivative in MeOH (3 mL) was prepared, to which 0.1M NaOH (4 equivalents per amine groups), triethylamine (8 equivalents per amine groups), and $SO_3$·Py (10 equivalents per amine groups) were added in portions at 1-hour intervals, maintaining the pH of the reaction mixture between 9 and 10. The mixture was stirred at room temperature, and the progress of the reaction was monitored by TLC using a solvent system of ethyl acetate/pyridine/water/acetic acid (8:5:3:1, v/v/v/v). After completion, the solvent was evaporated, and the crude product was purified using reverse-phase C18 column chromatography (MeOH/$H_2O$, 2:3 v/v), followed by passage through a DOWEX 50WX8 $Na^+$ resin column to obtain compound **13** in 58% yield. $^1$H NMR (600 MHz, Methanol-$d_4$) $\delta$: 7.95-7.70 (m, 9H), 7.58-7.42 (m, 6H), 7.41-7.17 (m, 19H), 5.33 (t, $J$ = 10.3 Hz, 2H), 5.18 (d, $J$ = 11.6 Hz, 1H), 5.04 (d, $J$ = 3.6 Hz, 2H), 4.99-4.79 (m, 1H), 4.78-4.67 (m, 2H), 4.64 (t, $J$ = 9.7 Hz, 2H), 4.59-4.55 (m, 1H), 4.55-4.47 (m, 2H), 4.47-4.29 (m, 3H), 4.27 (s, 1H), 4.19 (dd, $J$ = 17.5, 10.0 Hz, 3H), 4.10 (dd, $J$ = 11.5, 7.6 Hz, 1H), 4.02 (s, 1H), 3.92 (t, $J$ = 4.1 Hz, 1H), 3.89-3.72 (m, 6H), 3.64 (s, 1H), 3.56 (ddt, $J$ = 15.5, 10.5, 4.4 Hz, 2H), 3.30 (s, 1H), 3.22 (dt, $J$ = 13.3, 6.3 Hz, 1H), 3.12 (s, 1H), 2.83 (s, 1H), 1.83-1.78 (m, 2H). $^{13}$C NMR (101 MHz, Methanol-$d_4$) δ 174.98, 174.32, 157.37, 138.30, 138.06, 136.89, 136.29, 133.44, 133.05, 133.01, 128.16, 128.05, 127.87, 127.75, 127.64, 127.56 (d, $J$ = 2.9 Hz), 127.27, 127.17, 126.75, 125.67, 125.64, 125.53, 125.41, 125.33, 100.94, 77.91, 75.28, 75.00, 74.48, 71.39, 71.13, 70.47, 66.19, 66.01, 65.59, 38.62, 31.66, 29.33 (d, $J$ = 9.0 Hz), 24.60, 22.33, 13.03. HRMS m/z calculated for $C_{78}H_{85}N_3O_{29}S_2^{-2}$ 795.7361; found 795.7365.

**3-aminopropyl-*O*-[(2-sulfonatomido-2-deoxy-6-*O*-sulfonato-*α*-D-gluglucopyranosyl)-(1→4)-*O*-(*α*-L-idopyranosyluronate)-(1→4)-*O*-[(2-sulfonatomido-6-*O*-sulfonato-2-deoxy-*α*-D-gluglucopyranosyl)]-(1→4)-*O*-*α*-L-idopyranosiduronate (HSA-5).** Compound **13** was dissolved in water, and $Pd(OH)_2/C$ was added. The mixture was stirred under a hydrogen ($H_2$) atmosphere for 48 hours. Afterward, the reaction mixture was filtered, concentrated, and eluted through a Bond Elute C18 column using water as the eluent. The combined water fractions were pooled and lyophilized, yielding compound **HSA-5** in 62% yield. $^1$H NMR (400 MHz, Deuterium oxide) $\delta$: 5.48 (dt, $J$ = 10.5, 3.6 Hz, 1H), 5.35 (dd, $J$ = 8.9, 3.5 Hz, 2H), 5.27-5.09 (m, 1H), 5.02 (d, $J$ = 10.9 Hz, 1H), 4.97-4.89 (m, 2H), 4.30 (d, $J$ = 10.9 Hz, 1H), 4.22 (ddt, $J$ = 11.8, 8.7, 4.8 Hz, 4H), 4.11 (t, $J$ = 4.3 Hz, 3H), 4.02-3.82 (m, 3H), 3.82-3.66 (m, 4H), 3.57 (dd, $J$ = 8.7, 5.1 Hz, 2H), 3.42 (ddd, $J$ = 14.4, 10.8, 3.6 Hz, 1H), 3.25 (q, $J$ = 8.6 Hz, 1H), 3.16 (d, $J$ = 5.5 Hz, 3H), 2.03-1.97

(m, 3H). $^{13}C$ NMR (101 MHz, Deuterium Oxide) $\delta$: 100.72, 100.54, 96.50, 74.43, 71.01, 70.83, 70.43, 69.80, 69.12, 67.82, 67.51, 66.89, 66.75, 66.47, 66.19, 57.56, 54.12, 53.74, 38.28, 38.15, 26.23, 26.11. HRMS m/z calculated for $C_{27}H_{43}N_3O_{33}S_4^{-5}$ 213.0138; found 213.0139.

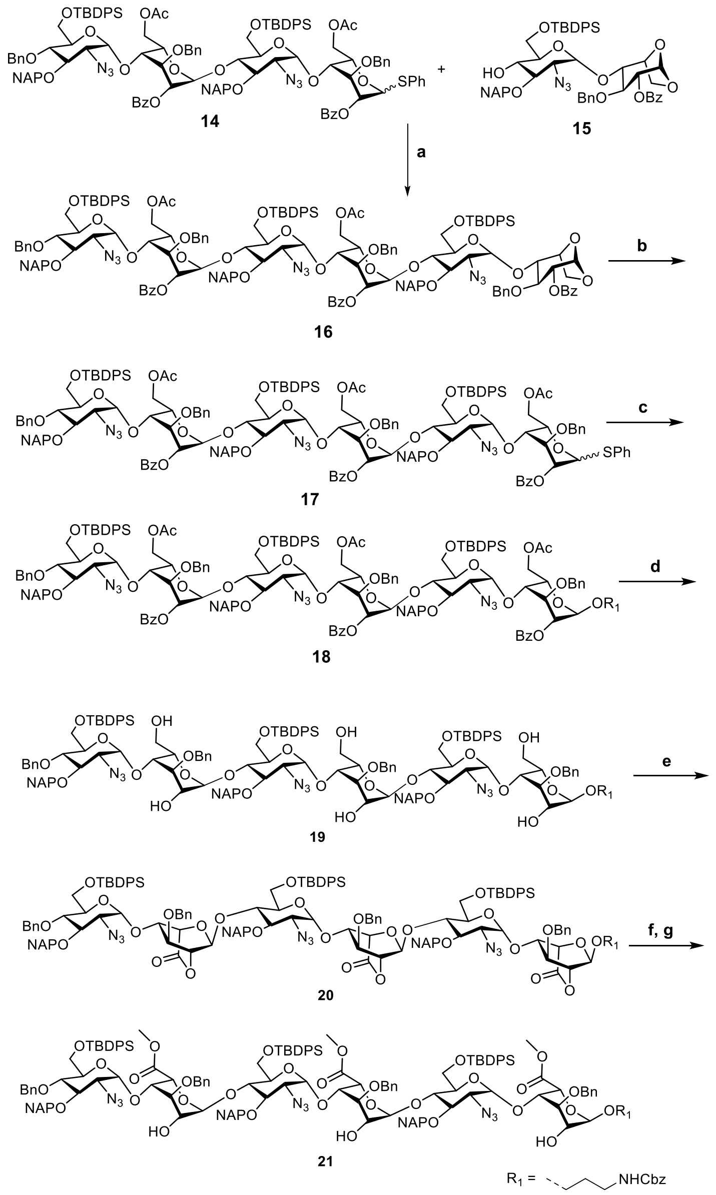

**Synthesis of hexasaccharide precursor.** **a**: NIS, TMSOTf, 4 Å MS, -10 ˚C, $CH_2Cl_2$, **b**: TMSSPh, $ZnI_2$, $CH_2Cl_2$, **c**: Benzyl (3-hydroxypropyl)carbamate, NIS, TMSOTf, 4 Å MS, -10 ˚C, $CH_2Cl_2$, **d**: NaOMe, $CH_2Cl_2$:MeOH (1:1), **e**: TEMPO, $CH_2Cl_2$:MeOH (1:1), **f**: $LiOH.H_2O$, $H_2O$:THF (1:1), **g**: MeI, $K_2CO_3$, DMF.

**1,6-Anhydro-[(2-azido-4-*O*-benzyl-6-*O*-*tert*-butyldiphenylsilyl-3-*O*-(2-naphthylmethyl)-2-deoxy-*α*-D-glucopyranosyl)-(1→4)-*O*-(2-*O*-benzoyl-3-*O*-benzyl-6-*O*-acetyl-*α*-L-**

**idopyranosyl)-(1→4)-*O*-(2-azido-4-*O*-benzyl-6-*O*-*tert*-butyldiphenylsilyl-3-*O*-(2-naphthylmethyl)-2-deoxy-*α*-D-glucopyranosyl)]-(1→4)-*O*-(2-*O*-benzoyl-3-*O*-benzyl-6-*O*-acetyl-*α* -L-idopyranosyl)-(1→4)-*O*-(2-azido-4-*O*-benzyl-6-*O*-*tert*-butyldiphenylsilyl-3-*O*-(2-naphthylmethyl)-2-deoxy-*α*-D-glucopyranosyl)]-(1→4)-*O*-2-*O*-benzoyl-3-*O*-benzyl-*β*-L-idopyranose (16).** A solution of tetrasaccharide donor **14** (0.87 g, 0.74 mmol) and disaccharide acceptor **15** (0.55 g, 0.60 mmol) in $CH_2Cl_2$ (15 mL) was stirred under a nitrogen atmosphere in a round-bottom flask containing freshly dried 4 Å molecular sieves for 2 hours. The mixture was cooled to -10°C, followed by the addition of NIS (0.26 g, 1.18 mmol) and TMSOTf (26 µL, 0.148 mmol). After 15 minutes, the reaction progress was monitored by TLC and subsequently quenched by the addition of a few drops of triethylamine. The molecular sieves were filtered through Celite, and the organic layer was washed with aqueous sodium thiosulfate and brine. The collected organic layer was dried over sodium sulfate, filtered, concentrated, and purified by silica gel column chromatography (ethyl acetate/hexane, 1:6 v/v) to yield compound **16** with a 90% yield. $^1$H NMR (400 MHz, Chloroform-d) $\delta$: 8.08 (d, $J$ = 1.2 Hz, 1H), 8.06-8.04 (m, 2H), 8.05-8.03 (m, 2H), 7.84-7.75 (m, 3H), 7.72-7.65 (m, 9H), 7.65-7.60 (m, 11H), 7.59-7.54 (m, 3H), 7.50-7.43 (m, 6H), 7.43-7.36 (m, 9H), 7.36-7.29 (m, 23H), 7.29-7.27 (m, 3H), 7.24-7.19 (m, 11H), 7.16-7.13 (m, 4H), 5.52 (d, $J$ = 1.8 Hz, 1H), 5.42 (s, 1H), 5.35 (d, $J$ = 2.2 Hz, 1H), 5.22 (t, $J$ = 2.9 Hz, 1H), 5.21-5.16 (m, 2H), 5.12 (d, $J$ = 11.3 Hz, 1H), 5.05 (dd, $J$ = 8.1, 1.8 Hz, 1H), 4.91-4.78 (m, 6H), 4.75-4.62 (m, 6H), 4.58 (d, $J$ = 10.7 Hz, 1H), 4.49-4.36 (m, 2H), 4.40-4.28 (m, 3H), 4.19-4.08 (m, 5H), 4.05-4.00 (m, 3H), 3.96-3.87 (m, 5H), 3.87-3.80 (m, 3H), 3.77-3.66 (m, 4H), 3.65-3.59 (m, 3H), 3.57-3.47 (m, 4H), 3.40 (dd, $J$ = 10.3, 3.8 Hz, 1H), 3.30-3.23 (m, 2H), 1.45 (s, 3H), 1.23 (s, 3H), 1.04 (d, $J$ = 1.9 Hz, 18H), 0.98 (s, 9H). $^{13}$C NMR (101 MHz, Chloroform-d) $\delta$: 170.1, 170.1, 165.9, 165.9, 165.8, 138.3, 138.0, 137.5, 137.5, 136.0, 136.0, 135.7, 135.7, 135.7, 135.5, 135.3, 133.5, 133.4, 133.4, 133.3, 133.3, 133.1, 133.1, 133.0, 132.9, 132.8, 130.0, 129.9, 129.9, 129.8, 129.8, 129.5, 128.9, 128.7, 128.6, 128.5, 128.5, 128.3, 128.2, 128.1, 128.0, 127.9, 127.9, 127.8, 127.8, 127.7, 127.5, 127.0, 126.5, 126.2, 126.1, 126.1, 126.1, 126.0, 125.9, 125.8, 99.4, 99.3, 98.3, 97.4, 97.1, 96.9, 79.3, 79.1, 78.9, 78.5, 77.7, 77.5, 77.4, 77.2, 77.0, 76.8, 75.4, 75.1, 75.1, 74.2, 73.7, 73.2, 72.9, 72.8, 64.4, 64.2, 64.1, 62.4, 62.2, 34.8, 27.0, 26.9, 26.8, 25.4, 20.3, 20.0, 19.5, 19.4. HRMS m/z calculated for $[M+Na]^+$ 2961.1589; found, 2961.1571.

**Phenyl-[(2-azido-4-*O*-benzyl-6-*O*-*tert*-butyldiphenylsilyl-3-*O*-(2-naphthylmethyl)-2-deoxy-*α*-D-glucopyranosyl)-(1→4)-*O*-(2-*O*-benzoyl-3-*O*-benzyl-6-*O*-acetyl-*α*-L-idopyranosyl)-**

**(1→4)-*O*-[(2-azido-4-*O*-benzyl-6-*O*-*tert*-butyldiphenylsilyl-3-*O*-(2-naphthylmethyl)-2-deoxy-*α*-D-glucopyranosyl)]-(1→4)-*O*-(2-*O*-benzoyl-3-*O*-benzyl-6-*O*-acetyl-*α*-L-idopyranosyl)-(1→4)-*O*-[(2-azido-4-*O*-benzyl-6-*O*-*tert*-butyldiphenylsilyl-3-*O*-(2-naphthylmethyl)-2-deoxy-*α*-D-glucopyranosyl)]-(1→4)-*O*-2-*O*-benzoyl-3-*O*-benzyl-6-*O*-acetyl-1-thio-*α*-L-idopyranoside (17).** A solution of compound **16** (1 g, 0.98 mmol) in acetic anhydride (10 mL) was stirred at ice-cold temperature for 15 minutes before the addition of $Cu(OTf)_2$ (0.035 g, 0.098 mmol). After 16 hours, the reaction mixture was concentrated under reduced pressure, and the residue was extracted with ethyl acetate and sodium bicarbonate, then washed with brine. The combined organic layer was dried over sodium sulfate, filtered, concentrated, and purified by silica gel column chromatography (ethyl acetate/hexane, 1:5 v/v) to yield the product with an 80% yield. Subsequently, a solution of the product (0.98 g, 0.88 mmol), zinc iodide (0.59 g, 1.84 mmol), and phenyl trimethylsilyl sulphide (0.50 g, 2.72 mmol) in $CH_2Cl_2$ (15 mL) was stirred under a nitrogen atmosphere for 2 hours. Upon completion, the reaction mixture was filtered through Celite, evaporated, and purified by silica gel column chromatography (ethyl acetate/hexane, 1:5 v/v) to obtain compound **17** with an 84% yield. $^{1}H$ NMR (400 MHz, Chloroform-d) $\delta$: 8.12-8.09 (m, 2H), 8.02 – 7.99 (m, 4H), 7.81-7.73 (m, 3H), 7.66-7.63 (m, 3H), 7.63-7.58 (m, 11H), 7.58-7.52 (m, 10H), 7.49 (s, 1H), 7.46-7.42 (m, 6H), 7.39-7.36 (m, 6H), 7.36-7.30 (m, 15H), 7.29-7.25 (m, 22H), 7.22-7.20 (dd, *J* = 7.4, 3.5 Hz, 6H), 7.18-7.14 (m, 3H), 7.14-7.07 (m, 6H), 5.61 (s, 1H), 5.38 (s, 1H), 5.34 (d, *J* = 5.7 Hz, 2H), 5.13 (d, *J* = 20.1 Hz, 2H), 4.92 (d, *J* = 11.7 Hz, 1H), 4.85-4.81 (m, 2H), 4.77 (d, *J* = 11.3 Hz, 2H), 4.69 (s, 2H), 4.67-4.60 (m, 3H), 4.58 (d, *J* = 3.5 Hz, 2H), 4.54 (d, *J* = 10.7 Hz, 1H), 4.46 (d, *J* = 3.8 Hz, 1H), 4.40 (d, *J* = 6.4 Hz, 1H), 4.36-4.4.31(m, 3H), 4.27 (d, *J* = 11.8 Hz, 1H), 4.23 (s, 1H), 4.15 (d, *J* = 9.5 Hz, 1H), 4.11 (d, *J* = 4.0 Hz, 1H), 4.07-4.01 (m, 3H), 4.02-3.95 (m, 5H), 3.90-3.87 (m, 1H), 3.85-3.73 (m, 6H), 3.68 (dd, *J* = 10.3, 5.2 Hz, 2H), 3.65-3.63 (m, 1H), 3.63-3.55 (m, 3H), 3.54-3.48 (m, 2H), 3.43-3.39 (m, 2H), 3.37 (s, 1H), 3.27-3.22 (m, 2H), 3.18 (dd, *J* = 10.3, 3.7 Hz, 1H), 1.84 (s, 3H), 1.23 (s, 3H), 1.17 (s, 3H), 1.00 (s, 9H), 0.98 (s, 9H), 0.94 (s, 9H). $^{13}C$ NMR (101 MHz, Chloroform-d) $\delta$: 170.4, 170.1, 170.0, 166.0, 165.9, 138.3, 137.6, 137.5, 137.4, 136.1, 136.0, 136.0, 135.8, 135.7, 135.7, 135.7, 135.5, 135.5, 135.3, 133.6, 133.5, 133.5, 133.4, 133.3, 133.3, 133.3, 133.2, 133.2, 133.1, 133.1, 132.8, 132.8, 131.8, 130.0, 129.9, 129.9, 129.9, 129.8, 129.8, 129.7, 129.7, 129.7, 129.6, 129.0, 128.9, 128.7, 128.7, 128.6, 128.6, 128.5, 128.5, 128.5, 128.5, 128.4, 128.3, 128.3, 128.3, 128.2, 128.2, 128.1, 127.9, 127.9, 127.8, 127.8, 127.8, 127.7, 127.7, 127.7, 127.5, 127.5, 127.4, 127.0, 126.2, 126.2, 126.1,

126.0, 126.0, 125.9, 125.8, 125.8, 125.6, 98.2, 98.1, 97.0, 97.0, 96.9, 96.7, 85.9, 80.4, 79.2, 78.8, 77.7, 77.5, 77.5, 77.4, 77.2, 76.8, 75.4, 75.1, 74.3, 73.4, 73.3, 73.1, 72.9, 72.9, 72.8, 72.3, 72.2, 72.1, 71.9, 71.6, 69.9, 68.8, 68.4, 66.4, 64.5, 64.4, 64.1, 63.0, 62.4, 62.3, 62.1, 62.0, 28.5, 26.9, 26.9, 26.8, 24.8, 23.9, 20.7, 20.0, 20.0, 19.5.

**N-benzyloxycarbonyl-3-aminopropyl-*O*-[(2-azido-4-*O*-benzyl-6-*O*-*tert*-butyldiphenylsilyl-3-*O*-(2-naphthylmethyl)-2-deoxy-*α*-D-glucopyranosyl)-(1→4)-*O*-(2-*O*-benzoyl-3-*O*-benzyl-6-*O*-acetyl-*α*-L-idopyranosyl)-(1→4)-*O*-[(2-azido-4-*O*-benzyl-6-*O*-*tert*-butyldiphenylsilyl-3-*O*-(2-naphthylmethyl)-2-deoxy-*α*-D-glucopyranosyl)]-(1→4)-*O*-(2-*O*-benzoyl-3-*O*-benzyl-6-*O*-acetyl-*α*-L-idopyranosyl)-(1→4)-*O*-[(2-azido-4-*O*-benzyl-6-*O*-*tert*-butyldiphenylsilyl-3-*O*-(2-naphthylmethyl)-2-deoxy-*α*-D-glucopyranosyl)]-(1→4)-*O*-2-*O*-benzoyl-3-*O*-benzyl-6-*O*-acetyl-1-*α*-L-idopyranoside (18).** A solution of donor **17** (0.90 g, 0.42 mmol) and linker benzyl (3-hydroxypropyl) carbamate (0.097 g, 0.46 mmol) in $CH_2Cl_2$ (15 mL) was stirred under a nitrogen atmosphere in a round-bottom flask containing freshly dried 4 Å molecular sieves for 2 hours. Subsequently, NIS (0.15 g, 0.67 mmol) and triflic acid (TMSOTf, 7.4 µL, 0.084 mmol) were added at room temperature. The reaction progress was monitored by TLC, and upon completion, it was quenched by the addition of a few drops of triethylamine. The molecular sieves were filtered through Celite, and the organic layer was washed with aqueous sodium thiosulfate and brine. The collected organic layer was dried over sodium sulfate, filtered, concentrated, and purified by silica gel column chromatography (ethyl acetate/hexane, 1:3 v/v) to yield compound **18** with a 78% yield. $^1$H NMR (400 MHz, Chloroform-d) $\delta$: 8.12-8.10 (m, 2H), 8.05-7.99 (m, 3H), 7.83-7.74 (m, 3H), 7.68-7.60 (m, 12H), 7.60-7.54 (m, 7H), 7.51 (s, 1H), 7.49-7.45 (m, 3H), 7.42-7.36 (m, 9H), 7.36-7.31 (m, 20H), 7.30-7.27 (m, 15H), 7.25-7.20 (m, 7H), 7.19-7.10 (m, 9H), 5.66 (t, $J$ = 5.5 Hz, 1H), 5.39 (d, $J$ = 14.4 Hz, 2H), 5.16 (d, $J$ = 20.4 Hz, 2H), 5.09 (s, 2H), 5.05 (s, 1H), 4.98 (s, 1H), 4.87-4.77 (m, 4H), 4.72 (d, $J$ = 7.9 Hz, 2H), 4.68 (d, $J$ = 3.0 Hz, 1H), 4.64 (d, $J$ = 3.0 Hz, 1H), 4.60 (d, $J$ = 3.5 Hz, 2H), 4.56 (d, $J$ = 10.7 Hz, 1H), 4.43-4.24 (m, 7H), 4.18-4.04 (m, 4H), 4.05-3.93 (m, 6H), 3.92-3.65 (m, 11H), 3.63-3.52 (m, 6H), 3.44 (d, $J$ = 7.1 Hz, 2H), 3.40 (s, 2H), 3.31-3.24 (m, 2H), 3.21 (dd, $J$ = 10.3, 3.5 Hz, 2H), 1.89-1.80 (m, 5H), 1.21 (d, $J$ = 14.5 Hz, 6H), 1.03 (s, 9H), 1.00 (s, 9H), 0.96 (s, 9H). $^{13}$C NMR (101 MHz, Chloroform-d) $\delta$: 170.5, 170.1, 170.0, 165.9, 165.9, 165.8, 156.7, 138.3, 137.6, 137.5, 136.8, 136.1, 136.0, 136.0, 135.8, 135.7, 135.7, 135.7, 135.5, 135.3, 133.6, 133.5, 133.4, 133.3, 133.3, 133.3, 133.1, 132.8, 132.8, 130.0, 129.9, 129.9, 129.8, 129.8, 129.8, 129.7, 129.7, 129.7, 129.6, 129.3, 128.9, 128.8, 128.7, 128.7, 128.6, 128.6,

128.5, 128.5, 128.5, 128.5, 128.4, 128.4, 128.3, 128.3, 128.2, 128.2, 128.1, 128.1, 128.0, 127.9, 127.9, 127.8, 127.8, 127.8, 127.7, 127.7, 127.7, 127.7, 127.6, 127.5, 127.5, 127.4, 127.0, 126.2, 126.2, 126.1, 126.0, 126.0, 125.9, 125.8, 125.8, 125.7, 125.3, 98.2, 98.2, 97.9, 97.0, 96.9, 96.7, 80.4, 79.2, 78.8, 77.7, 77.5, 77.4, 77.2, 76.8, 75.4, 75.1, 73.9, 73.2, 73.1, 72.9, 72.7, 72.6, 72.2, 72.1, 71.8, 71.6, 68.8, 68.4, 67.2, 66.7, 65.1, 64.9, 64.5, 64.4, 64.1, 63.2, 62.4, 62.0, 39.8, 29.8, 29.5, 27.0, 27.0, 26.9, 26.9, 26.8, 20.7, 20.0, 19.5, 19.5, 19.4.

**N-benzyloxycarbonyl-3-aminopropyl-*O*-[(2-azido-4-*O*-benzyl-6-*O*-*tert*-butyldiphenylsilyl-3-*O*-(2-naphthylmethyl)-2-deoxy-*α*-D-glucopyranosyl)-(1→4)-*O*-(3-*O*-benzyl-*α*-L-idopyranosyl)-(1→4)-*O*-[(2-azido-4-*O*-benzyl-6-*O*-*tert*-butyldiphenylsilyl-3-*O*-(2-naphthylmethyl)-2-deoxy-*α*-D-glucopyranosyl)]-(1→4)-*O*-(3-*O*-benzyl-*α*-L-idopyranosyl)-(1→4)-*O*-[(2-azido-4-*O*-benzyl-6-*O*-*tert*-butyldiphenylsilyl-3-*O*-(2-naphthylmethyl)-2-deoxy-*α*-D-glucopyranosyl)]-(1→4)-*O*-3-*O*-benzyl-*α*-L-idopyranoside (19).** To a solution of compound **18** (0.84 g, 0.37 mmol) in $CH_2Cl_2$ (7 mL) and MeOH (7 mL) was added NaOMe (0.06g, 1.11 mmol) and stirred at room temperature. After 12 h, reaction mixture was quenched using Amberlite IR-120(H) resin, filtered, evaporated and purified through silica gel column chromatography (ethyl acetate/ hexane, 1:2.5 v/v) to obtain compound **19** in 93 % yield. $^1$H NMR (400 MHz, Chloroform-d) $\delta$: 7.87-7.83 (m, 2H), 7.83-7.77 (m, 7H), 7.77-7.72 (m, 6H), 7.71-7.70 (m, 1H), 7.69 (d, $J$ = 1.3 Hz, 1H), 7.59-7.50 (m, 10H), 7.49-7.46 (m, 13H), 7.45-7.47 (d, $J$ = 1.5 Hz, 4H), 7.42-7.37 (m, 13H), 7.36-7.32 (m, 6H), 7.30-7.28 (m, 7H), 7.20-7.11 (m, 2H), 6.99-6.92 (m, 1H), 5.72 (d, $J$ = 7.5 Hz, 1H), 5.24 (d, $J$ = 2.0 Hz, 1H), 5.16 (s, 1H), 5.09-5.02 (m, 4H), 5.01-4.94 (m, 2H), 4.91-4.83 (m, 3H), 4.81 (s, 1H), 4.77-4.68 (m, 3H), 4.70 (s, 1H), 4.69-4.60 (m, 6H), 4.55 (d, $J$ = 12.1 Hz, 1H), 4.31-4.26 (m, 2H), 4.20 (t, $J$ = 6.8 Hz, 1H), 4.13-4.00 (m, 1H), 4.03 (t, $J$ = 9.5 Hz, 1H), 3.91 (d, $J$ = 6.0 Hz, 3H), 3.87 (s, 3H), 3.85 (s, 2H), 3.81-3.77 (m, $J$ = 13.8 Hz, 5H), 3.74-3.70 (m, 3H), 3.67 (d, $J$ = 5.0 Hz, 1H), 3.64 (d, $J$ = 3.5 Hz, 1H), 3.61 (d, $J$ = 4.3 Hz, 3H), 3.57 (s, 4H), 3.55 (s, 1H), 3.38 (d, $J$ = 10.3 Hz, 2H), 3.29-3.16 (m, 3H), 2.81-2.69 (m, 2H), 1.90-1.81 (m, 2H), 1.11 (s, 9H), 1.08 (s, 9H), 1.06 (s, 9H). $^{13}$C NMR (101 MHz, Chloroform-d) $\delta$: 156.8, 138.2, 137.6, 137.6, 137.5, 136.7, 136.2, 136.1, 135.9, 135.9, 135.9, 135.7, 135.7, 135.2, 135.2, 135.0, 133.6, 133.4, 133.2, 133.2, 132.9, 132.9, 132.7, 132.7, 130.0, 129.9, 129.9, 129.9, 129.8, 128.9, 128.8, 128.7, 128.7, 128.6, 128.6, 128.6, 128.5, 128.5, 128.4, 128.4, 128.2, 128.1, 128.0, 128.0, 127.9, 127.9, 127.8, 127.8, 127.8, 127.8, 127.7, 127.7, 127.6, 127.6, 127.2, 126.7, 126.6, 126.4, 126.3, 126.2, 126.2, 126.1, 126.0, 125.7, 125.7, 101.1, 100.2, 99.7, 94.9, 94.4, 94.0,

81.3, 80.1, 80.0, 77.9, 77.5, 77.4, 77.2, 76.8, 76.1, 75.6, 75.1, 73.8, 73.0, 72.9, 72.5, 72.4, 72.3, 72.1, 72.0, 70.4, 69.7, 69.3, 66.7, 66.3, 66.1, 64.7, 64.6, 64.1, 62.6, 62.0, 61.6, 61.5, 39.8, 36.7, 29.8, 27.0, 26.9, 26.9, 26.9, 19.5.

**N-benzyloxycarbonyl-3-aminopropyl-*O*-[(2-azido-4-*O*-benzyl-6-*O*-*tert*-butyldiphenylsilyl -3-*O*-(2-naphthylmethyl)-2-deoxy-*α*-D-glucopyranosyl)-(1→4)-*O*-(3-*O*-benzyl-*α*-L-idopyranosidurono-6,2-lactone)-(1→4)-*O*-[(2-azido-4-*O*-benzyl-6-*O*-*tert*-butyldiphenylsilyl-3-*O*-(2-naphthylmethyl)-2-deoxy-*α*-D-glucopyranosyl)]-(1→4)-*O*-(3-*O*-benzyl-*α*-L-idopyranosidurono-6,2-lactone)-(1→4)-*O*-[(2-azido-4-*O*-benzyl-6-*O*-*tert*-butyldiphenylsilyl-3-*O*-(2-naphthylmethyl)-2-deoxy-*α*-D-glucopyranosyl)]-(1→4)-*O*-3-*O*-benzyl-*α*-L-idopyranosidurono-6,2-lactone (20).** To a solution of compound **19** (0.67 g, 0.34 mmol) in $CH_2Cl_2$ (5 mL) and $H_2O$ (5 mL) was added TEMPO (0.01g, 0.07 mmol), BAIB (0.54 g, 1.70 mmol) and stirred at room temperature. After 16 h, the reaction mixture was extracted using saturated aqueous $NH_4Cl$. The collected organic layer was dried over $Na_2SO_4$, filtered, concentrated and purified through silica gel column chromatography (ethyl acetate/ hexane= 1/3, v/v) to obtain compound **20** in 56 % yield. $^1H$ NMR (400 MHz, Chloroform-d) $\delta$: 7.93-7.78 (m, 11H), 7.74-7.64 (m, 12H), 7.64-7.60 (m, 2H), 7.54-7.52 (m, 1H), 7.52-7.47 (m, 6 H), 7.46 (m, 3H), 7.45-7.43 (m, 4H), 7.42-7.39 (m, 14H), 7.38 (s, 3H), 7.37-7.33 (m, 12H), 7.32-7.29 (m, 9H), 7.19 (dd, $J$ = 6.6, 3.0 Hz, 2H), 5.87 (t, $J$ = 5.5 Hz, 1H), 5.51 (s, 1H), 5.45 (s, 1H), 5.26 (d, $J$ = 12.4 Hz, 1H), 5.21 – 5.15 (m, 1H), 5.11 (d, $J$ = 9.4 Hz, 2H), 5.05 (d, $J$ = 19.9 Hz, 2H), 5.00-4.95 (m, 2H), 4.93 (d, $J$ = 5.0 Hz, 1H), 4.88-4.81 (m, 2H), 4.80-4.72 (m, 2H), 4.70-4.64 (m, 2H), 4.61 (d, $J$ = 12.0 Hz, 1H), 4.54 (s, 1H), 4.51-4.46 (m, 2H), 4.35 (d, $J$ = 3.2 Hz, 1H), 4.31-4.27 (m, 1H), 4.26-4.21 (m, 3H), 4.20-4.16 (m, 2H), 4.03-3.93 (m, 4H), 3.90-3.83 (m, 3H), 3.83-3.77 (m, 4H), 3.77-3.70 (m, 4H), 3.67-3.63 (m, 3H), 3.62-3.54 (m, 4H), 3.62-3.55 (m, 4H), 3.46-3.28 (m, 3H), 2.87-2.79 (m, 1H), 1.94-1.85 (m, 2H), 1.10 (m, $J$ = 5.5 Hz, 27H). $^{13}C$ NMR (101 MHz, Chloroform-d) $\delta$: 167.6, 167.4, 167.2, 156.7, 138.2, 137.1, 137.1, 136.9, 136.8, 136.4, 136.2, 136.1, 136.1, 136.0, 135.7, 135.7, 135.6, 135.3, 133.7, 133.4, 133.4, 133.4, 133.2, 133.1, 133.0, 132.9, 132.9, 132.8, 132.7, 130.1, 130.0, 129.8, 129.8, 128.9, 128.8, 128.7, 128.6, 128.6, 128.6, 128.5, 128.4, 128.4, 128.3, 128.3, 128.3, 128.2, 128.2, 128.2, 128.1, 128.1, 128.0, 127.9, 127.8, 127.8, 127.8, 127.7, 127.6, 127.3, 126.7, 126.6, 126.3, 126.2, 126.1, 126.1, 125.2, 124.9, 124.8, 100.0, 99.0, 96.9, 96.8, 81.1, 80.6, 80.3, 80.1, 78.3, 78.2, 78.1, 77.8, 77.5, 77.4, 77.2, 76.9, 76.2, 76.1, 75.9, 75.1, 74.4, 72.8, 72.6, 72.4, 72.3, 72.2, 71.9, 69.5, 69.2, 66.7, 63.4, 63.2, 40.0, 36.7, 29.6, 27.0, 24.8, 19.5, 19.4.

**N-benzyloxycarbonyl-3-aminopropyl-*O*-[(methyl(2-azido-4-*O*-benzyl-6-*O*-*tert*-butyldiphenylsilyl-3-*O*-(2-naphthylmethyl)-2-deoxy-*α*-D-glucopyranosyl))]-(1→4)-*O*-(3-*O*-benzyl-*α*-L-idopyranosidurono-6,2-lactone)-(1→4)-*O*-(methyl(2-azido-4-*O*-benzyl-6-*O*-*tert*-butyldiphenylsilyl-3-*O*-(2-naphthylmethyl)-2-deoxy-*α*-D-glucopyranosyl))]-(1→4)-*O*-(3-*O*-benzyl-*α*-L-idopyranosidurono-6,2-lactone)-(1→4)-*O*-[methyl((2-azido-4-*O*-benzyl-6-*O*-*tert*-butyldiphenylsilyl-3-*O*-(2-naphthylmethyl)-2-deoxy-*α*-D-glucopyranosyl))]-(1→4)-*O*-3-*O*-benzyl-*α*-L-idopyranosyluronate (21).** The compound **20** was dissolved in THF:$H_2O$ (1:1) followed by addition of LiOH.$H_2O$ (10eq) and kept it on stirring for 2 h at room temperature followed by evaporation and dried over vaccum. After that reaction mixture was dissolved in dry DMF and $K_2CO_3$ (3eq), methyl iodide (9 eq) was added and kept it on stirring for at room temperature. After 12 h, extraction was done using 10 % HCl solution and DCM. Then, the organic layer was evaporated, purified using silica gel chromatography using ethyl acetate:hexane (2:3) to obtain compound **21** (78%). $^{1}$H NMR (400 MHz, Chloroform-d) $\delta$: 7.88-6.97 (m, 76H), 5.55 (d, $J$ = 5.9 Hz, 1H), 5.37 (d, $J$ = 21.1 Hz, 2H), 5.09-4.93 (m, 6H), 4.93-4.76 (m, 5H), 4.77-4.52 (m, 10H), 4.14-4.04 (m, 3H), 4.02-3.60 (m, 19H), 3.63-3.34 (m, 13H), 3.35-3.19 (m, 3H), 2.67 (d, $J$ = 6.7 Hz, 3H), 1.91-1.75 (m, 2H). $^{13}$C NMR (151 MHz, Chloroform-d) $\delta$: 169.7, 168.7, 168.7, 156.5, 138.3, 137.2, 137.1, 136.6, 136.1, 136.0, 136.0, 136.0, 135.9, 135.8, 135.6, 135.1, 135.1, 133.4, 133.4, 133.3, 133.3, 133.3, 133.2, 133.2, 133.1, 132.8, 132.8, 129.8, 129.8, 129.8, 128.8, 128.8, 128.8, 128.7, 128.6, 128.5, 128.4, 128.4, 128.4, 128.4, 128.3, 128.3, 128.2, 128.1, 128.0, 128.0, 127.9, 127.8, 127.8, 127.8, 127.7, 127.7, 127.6, 127.5, 127.5, 127.4, 127.2, 127.1, 126.5, 126.4, 126.2, 126.1, 126.0, 125.8, 125.7, 125.7, 125.6, 101.5, 100.6, 100.4, 95.0, 94.9, 94.3, 80.9, 79.5, 79.4, 77.5, 77.3, 77.1, 76.9, 76.0, 75.3, 75.1, 74.8, 73.7, 73.3, 73.1, 72.7, 72.7, 72.6, 72.6, 72.4, 72.1, 71.9, 71.8, 71.0, 70.7, 70.5, 68.0, 67.2, 66.9, 66.8, 66.7, 66.5, 64.4, 64.2, 63.9, 62.4, 62.1, 61.7, 52.2, 51.0, 50.9, 39.9, 36.7, 27.0, 26.9, 26.9, 26.9, 19.5, 19.4, 19.4. HRMS m/z calculated for $[M+Na]^+$ 2858.1450; found, 2858.1437.

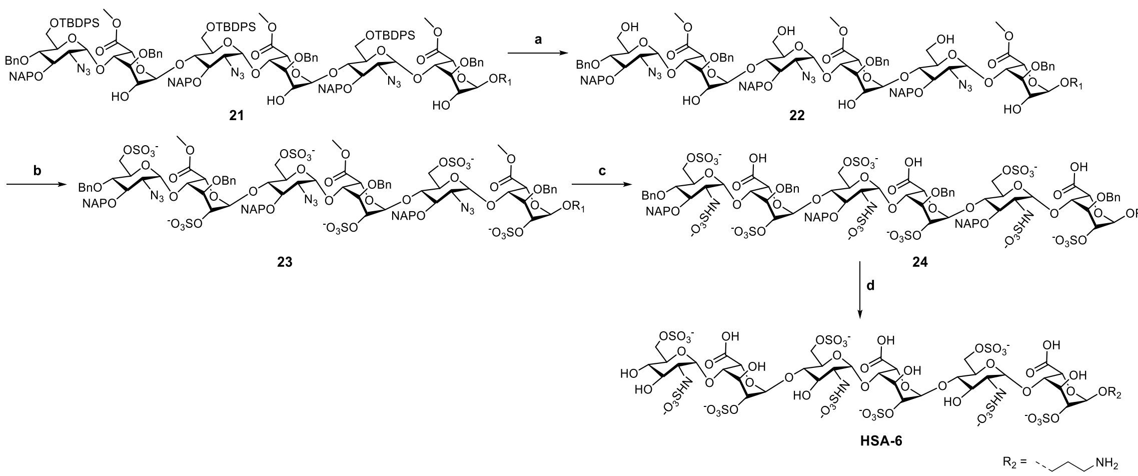

**Synthesis of HSA-6**. **a**: HF.Py., Py., 0 °C, 12 h, **b**: $SO_3$.$NEt_3$, DMF, 72 °C, 5 days, **c**: LiOH, MeOH; 1M $PMe_3$.THF, 0.1M $NaOH_{aq}$, $SO_3$.Py, MeOH, **d**: $Pd(OH)_2$/C/$H_2$, $H_2O$.

**N-benzyloxycarbonyl-3-aminopropyl-*O*-[(methyl(2-azido-4-*O*-benzyl-3-*O*-(2 naphthylmethyl)-2-deoxy-*α*-D-glucopyranosyl))]-(1→4)-*O*-(3-*O*-benzyl-*α*-L-idopyranosiduronate)-(1→4)-*O*-[(methyl(2-azido-3-*O*-(2-naphthylmethyl)-2-deoxy-*α*-D-glucopyranosyl))]-(1→4)-*O*-(3-*O*-benzyl-*α*-L-idopyranosiduronate)-(1→4)-*O*-[(methyl(2-azido-3-*O*-(2-naphthylmethyl)-2-deoxy-*α*-D-glucopyranosyl))]-(1→4)-*O*-3-*O*-benzyl- *α*-L-idopyranosiduronate (22)**. A solution of compound **21** (1 g, 1 eq) in dry pyridine (5 mL) was cooled to 0 °C, and a 70% HF·Py complex (5 eq per TBDPS) was added. The reaction mixture was stirred for 12 h. Afterward, ethyl acetate was added, and the mixture was washed three times with 1 N HCl. The organic layer was then evaporated, and the residue was purified by silica gel column chromatography (ethyl acetate:hexane, 2:3, v/v) to obtain compound **21** in 87% yield. $^1$H NMR (400 MHz, Chloroform-d) $\delta$: 7.84-7.55 (m, 10H), 7.48-7.17 (m, 36H), 5.61-5.53 (m, 1H), 5.32-5.16 (m, 2H), 5.05 (t, $J$ = 12.1 Hz, 3H), 5.01-4.88 (m, 3H), 4.89-4.59 (m, 12H), 4.51 (d, $J$ = 12.3 Hz, 1H), 4.07-3.83 (m, 9H), 3.79 (s, 8H), 3.73-3.65 (m, 3H), 3.67-3.38 (m, 14H), 3.36-3.17 (m, 3H), 2.98-2.86 (m, 4H), 1.91-1.78 (m, 2H). $^{13}$C NMR (101 MHz, Chloroform-d) $\delta$: 171.2, 170.0, 169.3, 169.2, 156.5, 137.8, 137.2, 137.0, 136.6, 135.0, 135.0, 133.4, 133.0, 132.9, 128.8, 128.8, 128.6, 128.5, 128.5, 128.4, 128.3, 128.2, 128.2, 128.1, 128.0, 127.9, 127.7, 127.6, 126.9, 126.2, 126.1, 126.0, 125.9, 125.8, 125.5, 101.7, 101.7, 100.8, 100.7, 95.3, 95.3, 80.8, 77.4, 77.3, 77.1, 76.7, 75.8, 75.2, 74.7, 72.8, 72.5, 72.2, 71.9, 71.2, 68.0, 66.7, 63.7, 61.0, 60.4, 52.5, 51.6, 51.5, 39.9, 29.5, 21.1, 14.2. HRMS m/z calculated for $[M+Na]^+$ 2120.8019; found, 2120.8011.

**N-benzyloxycarbonyl-3-aminopropyl-*O*-[(methyl(2-azido-4-*O*-benzyl-6-*O*-sulfonato-3-*O*-(2-naphthylmethyl)-2-deoxy-*α*-D-glucopyranosyl))]-(1→4)-*O*-(3-*O*-benzyl-2-*O*-sulfonato--*α*-L-**

**idopyranosiduronate)-(1→4)-*O*-[(methyl(2-azido-6-*O*-sulfonato-3-*O*-(2-naphthylmethyl)-2-deoxy-*α*-D-glucopyranosyl))]-(1→4)-*O*-(3-*O*-benzyl-2-*O*-sulfonato-*α*-L-idopyranosiduronate)-(1→4)-*O*-[(methyl(2-azido-6-*O*-sulfonato-3-*O*-(2-naphthylmethyl)-2-deoxy-*α*-D-glucopyranosyl))]-(1→4)-*O*-3-*O*-benzyl-2-*O*-sulfonato-*α*-L-idopyranosiduronate (23).** To a solution of starting material **22** (1 mmol, 1eq) in DMF (2 mL) was added $SO_3$.$NEt_3$ complex (10 mmol per OH, 30 eq) under $N_2$ atmosphere and stirred at 70 $^o$ C for 5 days. Upon completion of reaction, the reaction mixture was concentrated under reduced pressure and purified using silica column chromatography (MeOH:DCM, 1:10 v/v) to obtain product **23** in 67 % yield. $^1$H NMR (600 MHz, Methanol-d4) *δ*: 7.94-6.78 (m, 44H), 5.46-4.90 (m, 10H), 4.81-4.53 (m, 16H), 4.41-3.52 (m, 31H), 3.51-3.38 (m, 2H), 3.33-3.17 (m, 3H), 2.98-2.63 (m, 3H), 1.81-1.77 (m, 2H). $^{13}$C NMR (151 MHz, Methanol-d4) *δ*: 170.3, 170.1, 169.9, 157.4, 138.2, 138.0, 137.9, 137.7, 136.9, 135.5, 135.2, 133.4, 133.4, 133.3, 133.3, 133.1, 133.0, 133.0, 128.9, 128.9, 128.7, 128.5, 128.5, 128.4, 128.4, 128.4, 128.3, 128.2, 128.2, 128.1, 128.1, 128.0, 128.0, 127.9, 127.9, 127.8, 127.7, 127.7, 127.6, 127.6, 127.6, 127.5, 127.5, 127.5, 127.4, 127.3, 127.3, 127.3, 127.1, 127.0, 126.4, 126.3, 126.1, 126.0, 125.8, 125.7, 125.7, 125.7, 125.6, 125.5, 101.5, 100.7, 100.4, 96.2, 95.5, 95.4, 78.9, 77.2, 74.9, 74.6, 74.4, 74.3, 72.8, 72.6, 72.4, 70.7, 70.4, 68.3, 66.5, 66.1, 66.0, 65.7, 65.4, 64.0, 63.4, 53.5, 51.9, 51.8, 51.0, 50.9, 48.5, 48.3, 48.1, 48.0, 47.8, 47.7, 47.6, 47.4, 47.3, 38.1, 29.3. HRMS m/z calculated for $[M/6]^{6-}$ 432.5837; found,432.5829.

**N-benzyloxycarbonyl-3-aminopropyl-*O*-[(2-sulfonatoimido-4-*O*-benzyl-6-*O*-sulfonato-3-*O*-(2-naphthylmethyl)-2-deoxy-*α*-D-glucopyranosyl))]-(1→4)-*O*-(3-*O*-benzyl-2-*O*-sulfonato-*α*-L-idopyranosiduronate)-(1→4)-*O*-(2-sulfonatoimido-6-*O*-sulfonato-3-*O*-(2-naphthylmethyl)-2-deoxy-*α*-D-glucopyranosyl)]-(1→4)-*O*-(3-*O*-benzyl-2-*O*-sulfonato-*α*-L-idopyranosiduronate)-(1→4)-*O*-(2-sulfonatoimido-6-*O*-sulfonato-3-*O*-(2-naphthylmethyl)-2-deoxy-*α*-D-glucopyranosyl)]-(1→4)-*O*-3-*O*-benzyl-2-*O*-sulfonato-*α*-L-idopyranosiduronate (24).** To a solution of compound 23 (1 g, 1 eq) in THF:$H_2O$ (1:1, 5 mL), LiOH·$H_2O$ (20 eq) was added, and the reaction mixture was stirred at room temperature. After 12 hours, the reaction was quenched using IR $H^+$ resin, evaporated, and purified by reverse-phase C18 column chromatography (MeOH:$H_2O$, 1:9 v/v). The resulting compound (1 g) was dissolved in THF (3 mL), and 1 M $PMe_3$·THF (8 eq) and 0.1 M aqueous NaOH (10 eq) were added. The reaction mixture was stirred at room temperature for 12 hours, then quenched with glacial acetic acid. The solvent was evaporated, and the residue was purified by reverse-phase C18 column

chromatography (MeOH:$H_2O$, 2:3 v/v), followed by passage through a DOWEX 50WX8 $Na^+$ resin column. Subsequently, the residue (1 g) was dissolved in MeOH (3 mL), and 0.1 M NaOH (4 eq), triethylamine (8 eq), and $SO_3$·Py (10 eq) were added at 1-hour intervals over six additions, maintaining the pH of the reaction mixture at 9–10. The mixture was stirred at room temperature, and the reaction progress was monitored by TLC (ethyl acetate/pyridine/water/acetic acid, 8:5:3:1 v/v/v/v). After completion, the solvent was evaporated, and the residue was purified by reverse-phase C18 column chromatography (MeOH:$H_2O$, 2:3 v/v), followed by passage through a DOWEX 50WX8 $Na^+$ resin column. $^1$H NMR (400 MHz, Deuterium oxide) $\delta$: 8.23-7.02 (m, 47H), 5.42-4.92 (m, 16H), 4.82-4.54 (m, 8H), 4.52-3.59 (m, 27H), 3.58-3.13 (m, 5H), 1.93-1.76 (m, 2H). $^{13}$C NMR (151 MHz, Methanol-d) $\delta$: 174.8, 174.1, 172.4, 157.7, 138.7, 138.6, 138.5, 137.0, 136.7, 136.1, 133.4, 133.4, 133.2, 133.1, 133.0, 128.4, 128.4, 128.2, 128.1, 128.1, 128.0, 128.0, 127.9, 127.9, 127.9, 127.8, 127.8, 127.7, 127.7, 127.6, 127.6, 127.5, 127.4, 127.4, 127.4, 127.3, 127.3, 127.3, 127.2, 127.2, 127.1, 127.1, 127.0, 126.9, 126.5, 125.8, 125.8, 125.6, 125.4, 125.4, 125.3, 103.1, 102.0, 97.6, 96.9, 93.6, 92.7, 81.6, 80.6, 78.1, 77.7, 77.3, 76.2, 75.3, 75.1, 74.9, 74.8, 74.6, 74.4, 74.3, 73.2, 72.7, 70.5, 70.0, 69.7, 67.1, 67.0, 66.0, 65.9, 58.6, 53.8, 49.9, 48.5, 48.2, 48.1, 48.0, 47.8, 47.7, 47.5, 47.4, 47.2, 43.1, 42.1, 37.3, 29.5, 29.4, 29.3, 29.3, 21.4, 12.6, 12.5, 12.5, 10.5. HRMS m/z calculated for $[M/6]^{-6}$ 271.2333; found,271.2321.

**3-aminopropyl-*O*-[(2-sulfonatoimido-6-*O*-sulfonato-2-deoxy-*α*-D-glucopyranosyl)]-(1→4)-*O*-(2-*O*-sulfonato-*α*-L-idopyranosiduronate)-(1→4)-*O*-(2-sulfonatoimido-4-*O*-benzyl-6-*O*-sulfonato-2-deoxy-*α*-D-glucopyranosyl)]-(1→4)-*O*-(2-*O*-sulfonato-*α*-L-idopyranosiduronate)-(1→4)-*O*-(2-sulfonatoimido-6-*O*-sulfonato-2-deoxy-*α*-D-glucopyranosyl)]-(1→4)-*O*-2-*O*-sulfonato-*α*-L-idopyranosiduronate (HSA-6).** $Pd(OH)_2$/C was added to a solution of compound **24** in $H_2O$ and stirred under $H_2$ atmosphere. After 48 h, the mixture was filtered, concentrated, and eluted through a Bond Elute C18 column using $H_2O$ as the eluent. The combined $H_2O$ fractions were pooled and lyophilized to yield the fully deprotected NS-hexasaccharide. $^1$H NMR (400 MHz, Deuterium oxide) $\delta$: 5.35 (d, $J$ = 9.5 Hz, 3H), 5.27-5.13 (m, 2H), 5.05 (s, 1H), 4.98-4.83 (m, 4H), 4.43-4.26 (m, 4H), 4.22 (d, $J$ = 13.9 Hz, 4H), 4.14-4.09 (m, 3H), 3.95-3.90 (m, 1H), 3.82 (s, 3H), 3.80-3.67 (m, 5H), 3.65-3.52 (m, 4H), 3.29 3.20 (m, 3H), 3.20-3.03 (m, 3H), 2.06-1.94 (m, 2H). $^{13}$C NMR (101 MHz, Deuterium oxide) $\delta$: 174.9, 174.8, 174.8, 102.2, 101.8, 97.6, 97.0, 95.2, 77.1, 76.7, 76.5, 76.2, 76.2, 76.0, 75.8, 73.2, 73.1, 72.8, 72.6,

71.1, 70.5, 69.8, 69.3, 69.2, 69.0, 68.6, 68.0, 67.7, 66.3, 66.0, 65.8, 57.9, 57.4, 54.0, 53.7, 37.6, 37.6, 26.7. HRMS m/z calculated for $[M/9]^{9-}$ 199.6575; found, 199.6568.

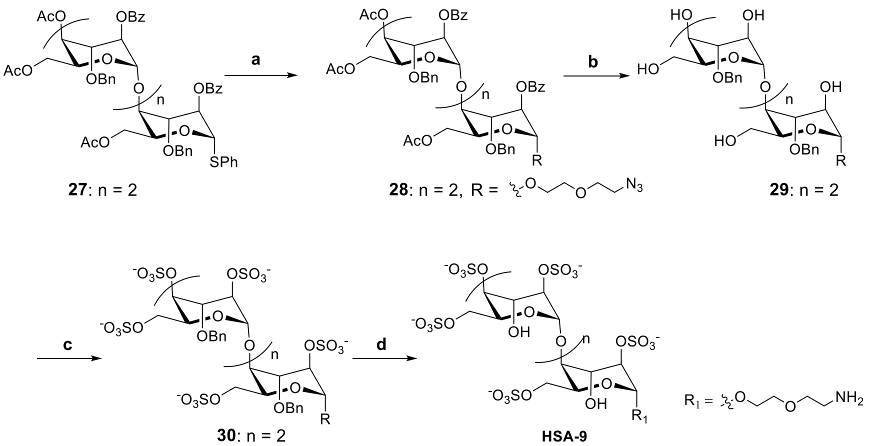


**Synthesis of compound HSA-9. a**: azidoethoxyethanol, NIS TMSOTf, 4Å molecular sieves, -10˚C, **b**: LiOH, THF/MeOH/$H_2O$ (4/2/1), 0˚C, **c**: $SO_3.NMe_3$, DMF, 60˚C, **d**: $H_2$/Pd$(OH)_2$/C, $H_2O$.

**Ethoxy-2-azidoethoxyl-O-((4,6-O-diacetyl-2-O-benzoly-3-O-benzyl)-α(1→4)-L-idopyrnosyl-(6-O-acetyl-2-O-benzoly-3-O-benzyl)-α(1→4)-L-idopyrnosyl-(6-O-acetyl-2-O-benzoly-3-O-benzyl))-α(1→4)-L-idopyrnoside (28).** Trisaccharide donor **27** (300 mg, 0.222 mmol), azido ethoxy ethanol linker (23 mg, 0.178 mmol), and freshly dried 4 Å molecular sieves were combined in a round-bottom flask and dissolved in dry DCM at a volume ratio of 1:10. The mixture was stirred for 2 h under a nitrogen atmosphere, then cooled to -10 °C. TMSOTf (8 µL, 0.044 mmol) and NIS (59.8 mg, 0.267 mmol) were added, and the mixture was stirred. The completion of the reaction was monitored by TLC. After the reaction was complete, it was neutralized with triethylamine ($Et_3N$), followed by filtration through Celite. The reaction mixture was then subjected to a sodium thiosulfate ($Na_2S_2O_3$) workup and dried over $Na_2SO_4$.Purification was performed by silica gel column chromatography using an EtOAc/hexane solvent system to obtain compound 29 in 85% yield. $^1$H NMR (400 MHz, Chloroform-*d*) $\delta$: 5.36 (dt, $J$ = 15.8, 2.7 Hz, 2H), 5.16-5.12 (m, 1H), 5.07-4.98 (m, 2H), 4.92-4.78 (m, 4H), 4.68 (dt, $J$ = 13.5, 10.5 Hz, 3H), 4.58 (t, $J$ = 2.3 Hz, 1H), 4.53-4.25 (m, 5H), 4.17-3.99 (m, 3H), 3.99-3.77 (m, 5H), 3.76-3.46 (m, 7H), 3.21 (ddd, $J$ = 5.9, 4.1, 3.0 Hz, 2H), 1.98 (s, 3H), 1.94 (s, 3H), 1.90 (s, 3H), 1.87 (s, 3H), 8.04-7.99 (m, 2H), 7.99 – 7.88 (m, 4H), 7.62-7.55 (m, 1H), 7.52-7.33 (m, 10H), 7.29 (td, $J$ = 7.3, 5.4 Hz, 6H), 7.25-7.15 (m,

7H). $^{13}C$ NMR (101 MHz, Chloroform-*d*) $\delta$: 177.47, 170.60, 170.53, 170.51, 169.95, 165.60, 165.38, 165.00, 137.92, 137.51, 137.21, 133.64, 133.36, 133.09, 130.15, 130.00, 129.76, 129.40, 129.32, 129.12, 128.49, 128.42, 128.36, 128.23, 128.20, 128.04, 127.87, 127.78, 127.69, 101.17, 100.65, 98.39, 77.47, 77.34, 77.15, 76.84, 76.73, 76.23, 75.00, 74.47, 72.93, 72.51, 72.38, 72.28, 70.39, 70.29, 70.20, 68.21, 67.73, 67.63, 67.46, 66.69, 65.99, 65.16, 64.10, 62.79, 62.22, 50.73, 31.93, 20.77, 20.75, 20.62, 20.57, 0.04. HRMS m/z calculated for $C_{72}H_{72}N_3O_{24}Na$, 1369.4897; found 1369.4899.

**Ethoxy-2-azidoethoxyl-O-((3-O-benzyl)-α(1→4)-L-idopyrnosyl-(3-O-benzyl))-α(1→4)-L-idopyrnosyl-(3-O-benzyl))-α(1→4)-L-idopyrnoside (29)**. Compound **28** (106 mg, 0.077 mmol) was dissolved in THF/MeOH/$H_2O$ (4:2:1), and LiOH (113 mg, 2.71 mmol) was added at 0 °C. The mixture was stirred for 3-4 hours, and the completion of the reaction was monitored by TLC. After completion, the reaction mixture was neutralized with Amberlite IR-120(H) resin. Subsequently, the mixture was filtered using cotton plug filtration and concentrated under reduced pressure. Purification was carried out by silica gel column chromatography using a MeOH/DCM solvent system to obtain compound **29** in 95% yield. $^{1}H$ NMR (400 MHz, Chloroform-*d*) $\delta$: 4.90 (s, 1H), 4.83 (s, 1H), 4.79 (s, 1H), 4.72 (d, $J$ = 11.8 Hz, 1H), 4.62-4.49 (m, 6H), 4.43 (d, $J$ = 8.5 Hz, 1H), 4.33 (dt, $J$ = 15.7, 6.6 Hz, 2H), 4.16 (dd, $J$ = 11.7, 5.5 Hz, 2H), 4.01 (d, $J$ = 3.8 Hz, 1H), 3.96-3.74 (m, 8H), 3.72-3.52 (m, 15H), 3.47 (dd, $J$ = 12.1, 3.4 Hz, 1H), 3.22 (t, $J$ = 5.0 Hz, 2H), 7.38-7.26 (m, 15H). $^{13}C$ NMR (101 MHz, Chloroform-*d*) $\delta$: 137.08, 128.58, 128.39, 128.34, 128.24, 127.78, 127.56, 103.08, 101.28, 77.28, 74.41, 74.26, 74.07, 73.07, 72.77, 72.62, 71.97, 71.46, 70.39, 70.09, 67.52, 66.73, 66.54, 66.47, 66.29, 64.37, 62.02, 61.05, 50.75. HRMS m/z calculated for $C_{43}H_{57}N_3O_{17}$, 887.3688; found 887.3691.

**Ethoxy-2-azidoethoxyl-O-((2,4,6-O-trisulfonato-3-O-benzyl)-α(1→4)-L-idopyrnosyl-(2,6-O-disulfonato-3-O-benzyl)-α(1→4)-L-idopyrnosyl-(2,6-O-disulfonato-3-O-benzyl))-α(1→4)-L-idopyrnoside (30).** Compound **29** (32 mg, 0.03606 mmol) and $SO_3 \cdot NMe_3$ (350 mg, 2.524 mmol) were thoroughly dried together under high vacuum, then dissolved in dry DMF and stirred at 60 °C for 72 h. The DMF was subsequently evaporated under reduced pressure, and the residue was purified by Bond Elute C18 column chromatography to obtain compound **30** in 80% yield. $^{1}H$ NMR (600 MHz, Deuterium oxide) $\delta$: 5.05 (s, 1H), 4.96 (s, 1H), 4.86 (s, 1H), 4.79-4.72 (m, 5H), 4.69-4.61 (m, 3H), 4.58-4.51 (m, 1H), 4.41-4.30 (m, 4H), 4.26 (t, $J$ = 1.8 Hz, 1H), 4.17 (dd, $J$ =

11.6, 8.5 Hz, 1H), 4.14-4.06 (m, 6H), 3.98 (dd, *J* = 10.6, 3.4 Hz, 1H), 3.89-3.83 (m, 1H), 3.73-3.67 (m, 3H), 3.67-3.60 (m, 4H), 3.36-3.31 (m, 2H), 7.49-7.23 (m, 15H). $^{13}$C NMR (151 MHz, Deuterium Oxide) *δ*: 137.74, 136.96, 128.82, 128.71, 128.65, 128.62, 128.60, 128.54, 128.24, 128.16, 128.06, 100.56, 100.40, 98.43, 75.53, 75.08, 74.41, 73.18, 73.06, 72.37, 71.82, 71.46, 70.91, 70.25, 69.37, 69.22, 67.99, 67.95, 67.69, 67.24, 65.97, 65.73, 64.12, 50.19.HRMS m/z calculated for $C_{43}H_{50}N_3O_{38}S_7^{-7}$, 205.7165; Found 205.7169.

**Ethoxy-2-aminoethoxy-O-((2,4,6-O-trisulfonato)-α(1→4)-L-idopyrnosyl-(2,6-O-disulfonato)-α(1→4)-L-idopyrnosyl-(2,6-O-disulfonato))-α(1→4)-L-idopyrnoside (HSA-9).**

Compound **30** was dissolved in water, and $Pd(OH)_2/C$ was added. The mixture was stirred under a hydrogen ($H_2$) atmosphere for 48 hours. Afterward, the reaction mixture was filtered, concentrated, and eluted through a Bond Elute C18 column using water as the eluent. The combined water fractions were pooled and lyophilized, yielding Compound **HSA-9** in 80% yield. $^1$H NMR (600 MHz, Deuterium oxide) *δ*: 5.16 (dd, *J* = 15.3, 3.8 Hz, 3H), 4.72 (dt, *J* = 9.7, 3.3 Hz, 1H), 4.54 (dtd, *J* = 11.3, 8.0, 6.9, 4.0 Hz, 1H), 4.45-4.23 (m, 15H), 4.07-3.66 (m, 10H), 3.31-3.16 (m, 2H). $^{13}$C NMR (151 MHz, Deuterium oxide) δ 100.27, 100.06, 98.56, 76.16, 75.87, 74.75, 73.06, 72.70, 72.57, 71.98, 69.62, 68.10, 67.89, 67.79, 67.64, 67.40, 67.36, 67.03, 66.72, 66.23, 66.12, 65.38, 64.23, 39.16. HRMS m/z calculated for $C_{22}H_{34}NO_{38}S_7^{-7}$, 163.4120; found 163.4124.

## 18. Supplementary figures

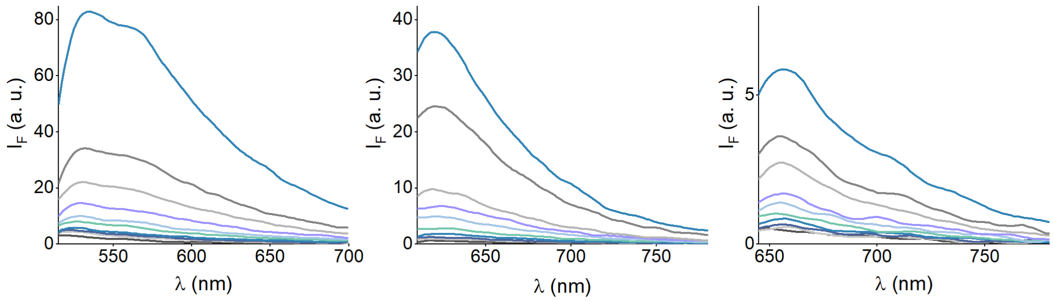


**Figure S1.** Fluorescence spectra of probe **1** (left), **2** (middle), and **3** (right) in PBS–glycerol mixtures (0–90% v/v glycerol, 10% intervals) at 25 °C.

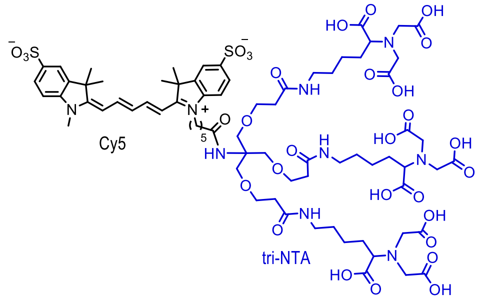


**Figure S2.** Chemical structure of the 'always on' probe **5**.

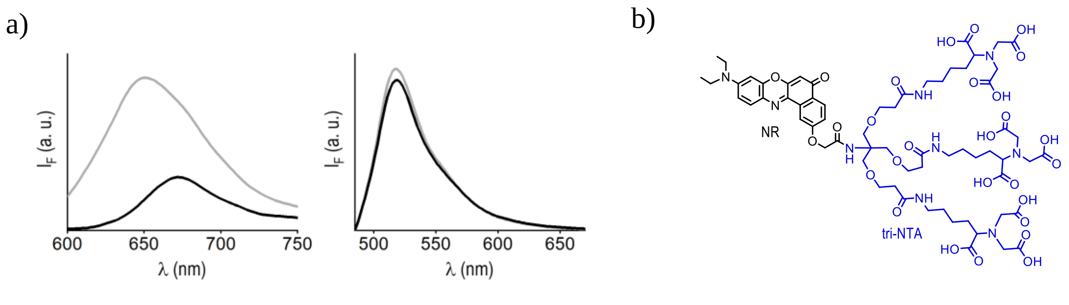


**Figure S3.** a) Fluorescence spectra of 100 nM Nile Red (NR)-appended probe (tri-NTA-NR conjugate) (left) and probe **4** (right) in the absence (black line) and presence (grey line) of His-tagged bacteria and $Ni^{2+}$. b) Chemical structure of the NR-appended probe.

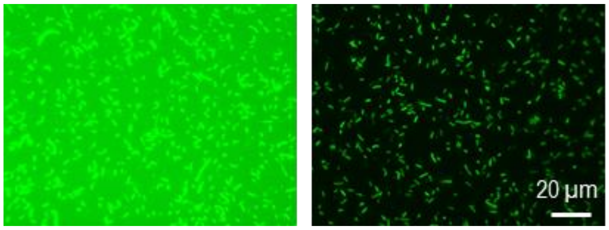


**Figure S4.** Fluorescence image of His-bacteria treated with 'always on' probe (500 nM) in the presence of $Ni^{2+}$ before (left) and after (right) washing.

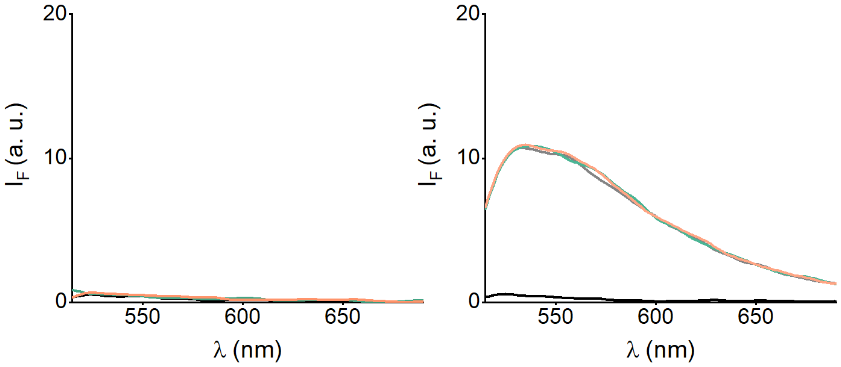


**Figure S5.** Left: Fluorescence spectra of probe **1** (300 nM) in the absence (black line) and in the presence of heparin at 10 µM (green line) and 100 µM (orange line). Right: Fluorescence spectra of probe **1** (300 nM) in the absence (black line), in the presence of BSA (150 µM, grey line), and in the presence of BSA with heparin at 10 µM (green line) or 100 µM (orange line).

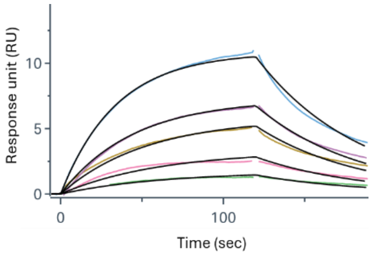


**Figure S6**: SPR sensorgrams (colored lines) and fitted binding curves (black lines) for the interactions between RBD and HSA-8 at concentrations of 0.15, 0.3, 1.25, 2.5, and 5 µM. Data were fitted using a kinetic 1:1 binding model.

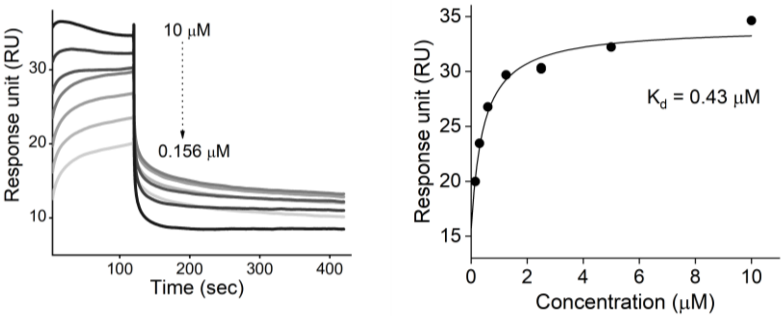


**Figure S7.** SPR sensorgrams and the corresponding binding curve for determining the binding constant of heparin to RBD. Data were fitted using a steady-state affinity model.

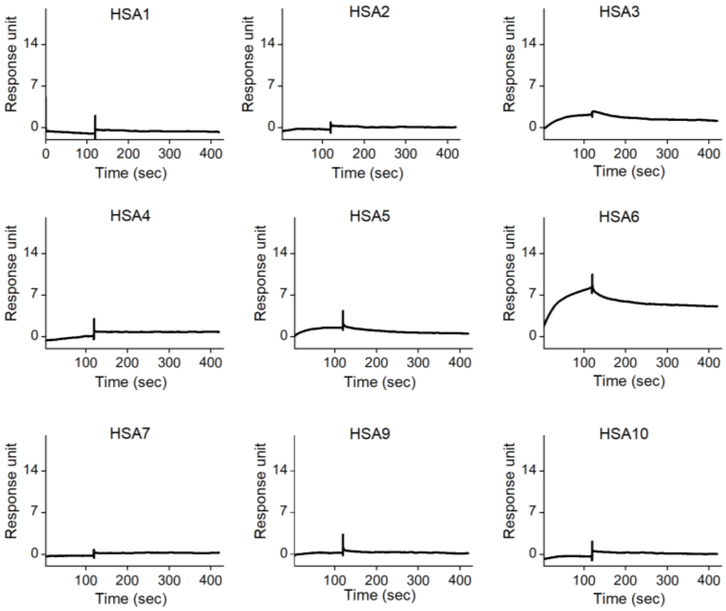


**Figure S8.** SPR data showing the binding response of different HSAs at a concentration of 10 μM.

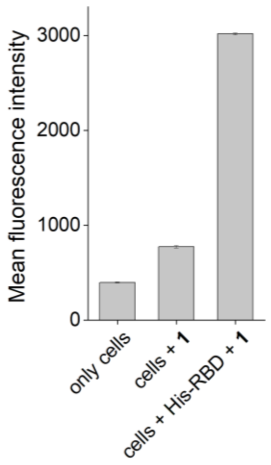


**Figure S9.** FACS analysis of mean fluorescence intensity (MFI) of cells before and after incubation with probe **1**, as well as with both His-RBD and probe **1**. Statistical significance ($p < 0.001$) was determined using F-test.

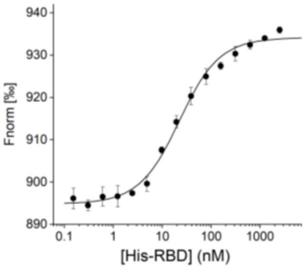


**Figure S10.** MST binding curves obtained by titrating increasing concentrations of His-RBD against probe **5** (5 nM) in the presence of $Ni^{2+}$.

## 19. NMR spectra of synthesized compounds

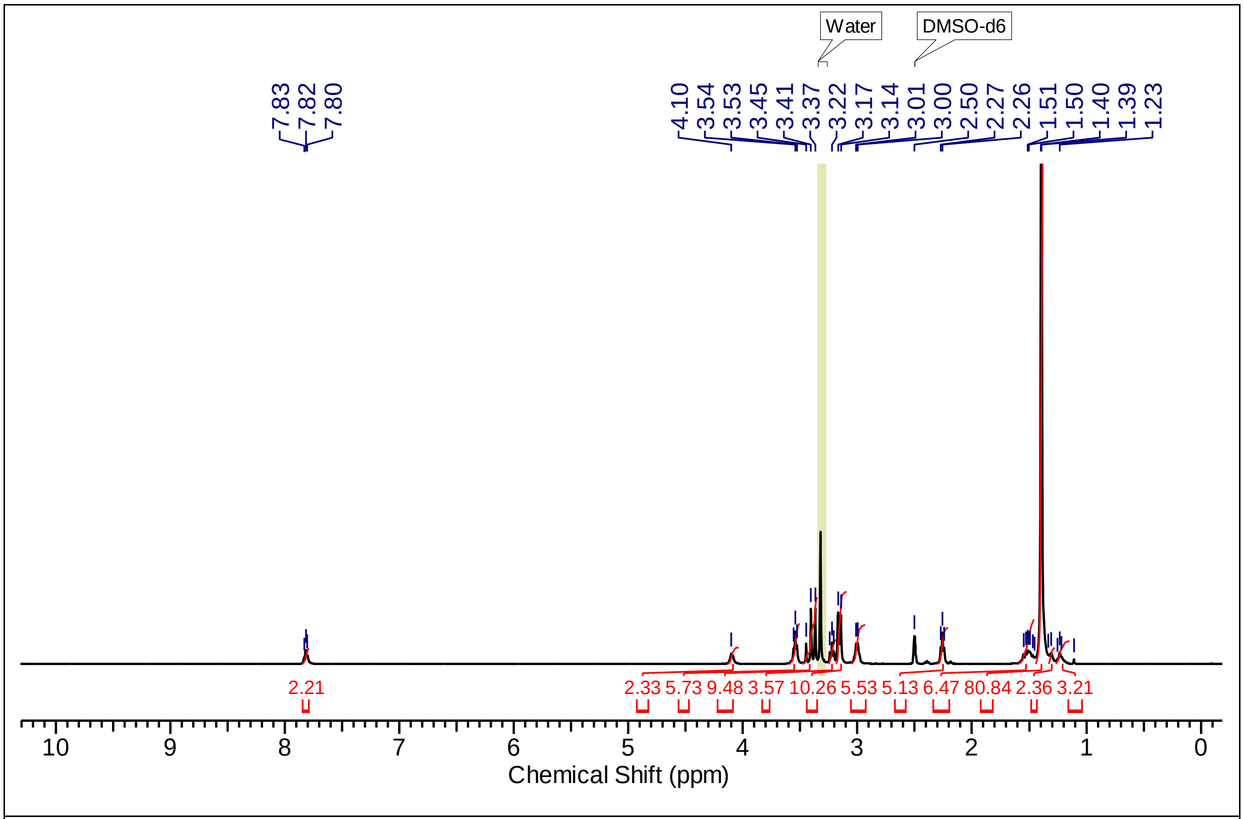


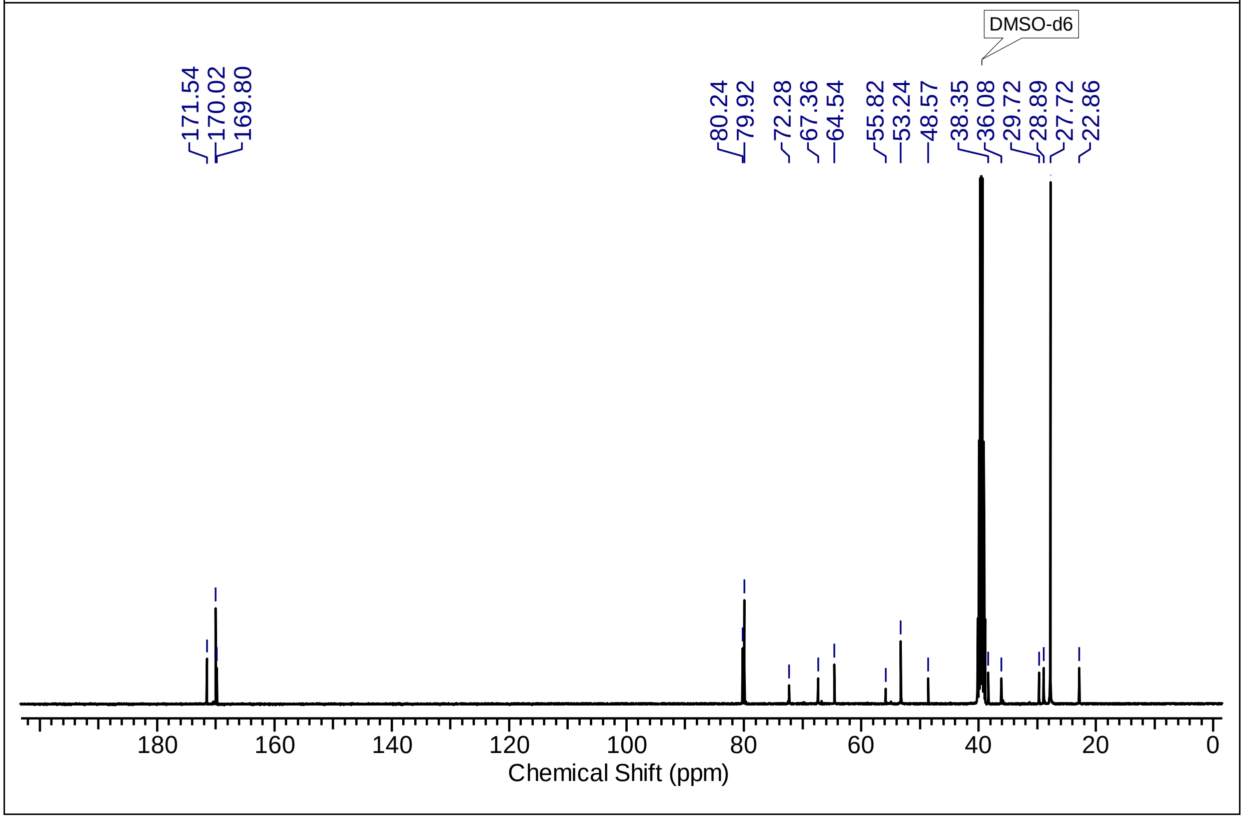

$^{1}$H and $^{13}$C NMR of compound **CP-1**

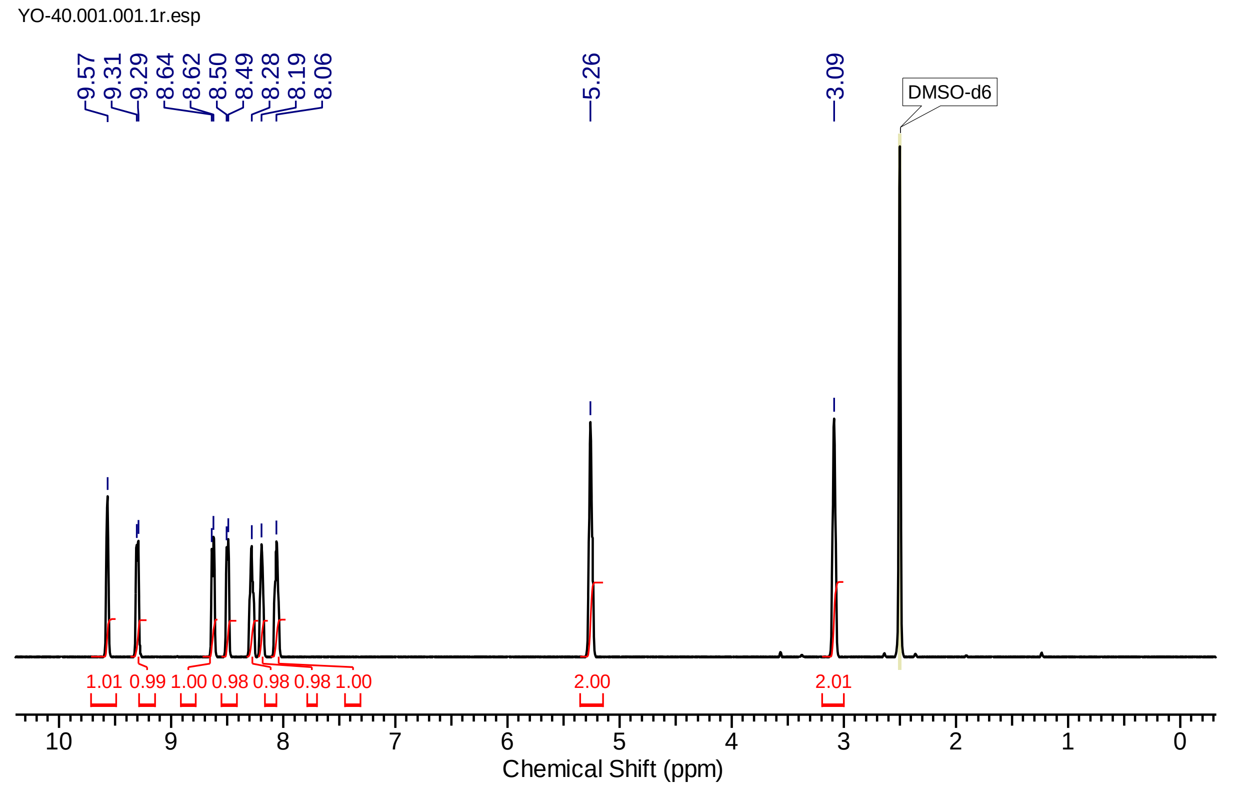


$^{1}$H NMR of compound **C1**

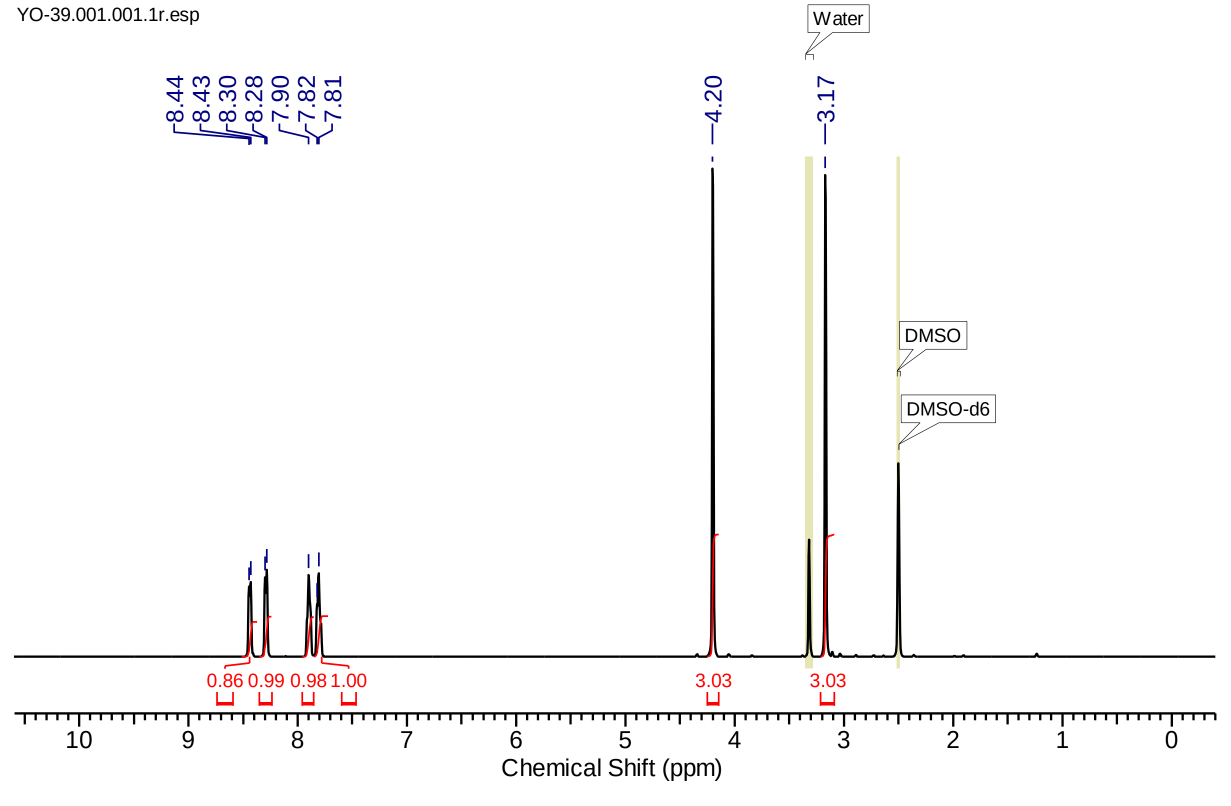


$^{1}$H NMR of compound **C2**

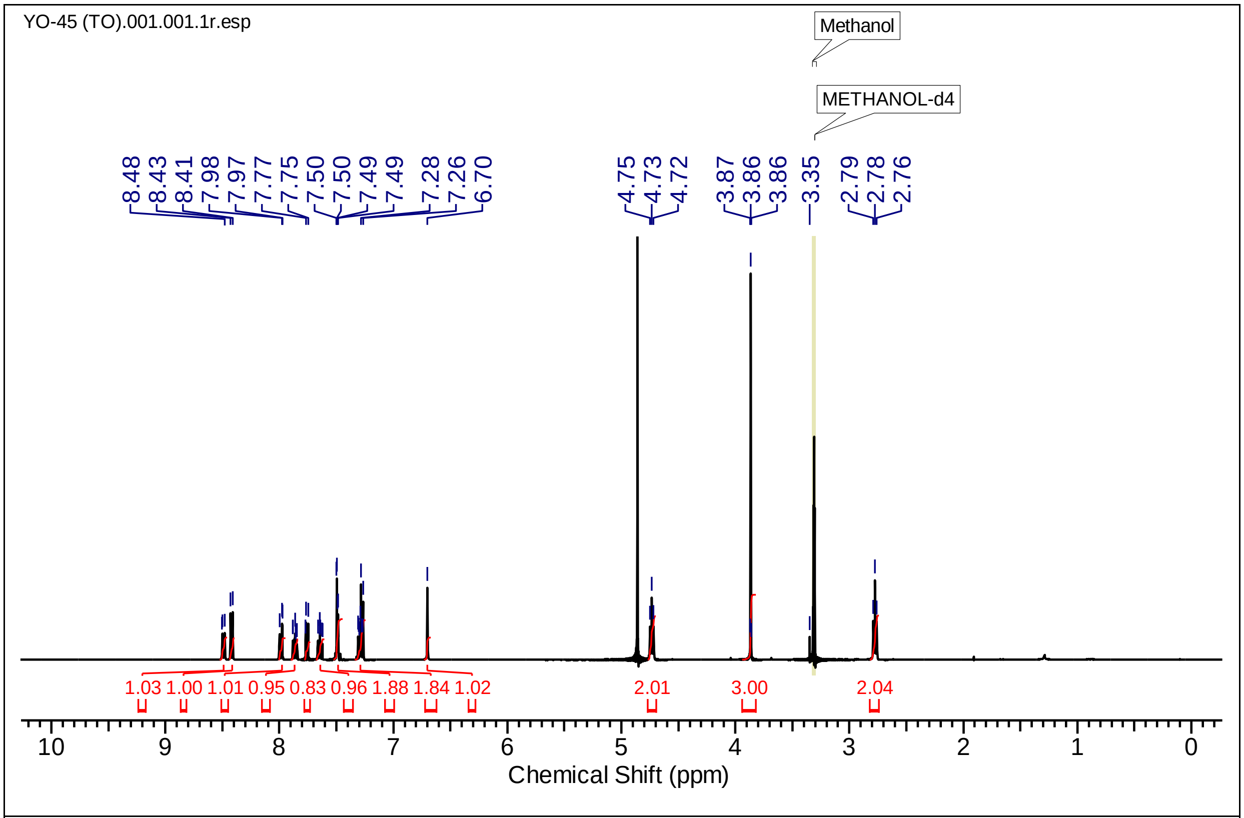


$^{1}$H NMR of compound **C3**

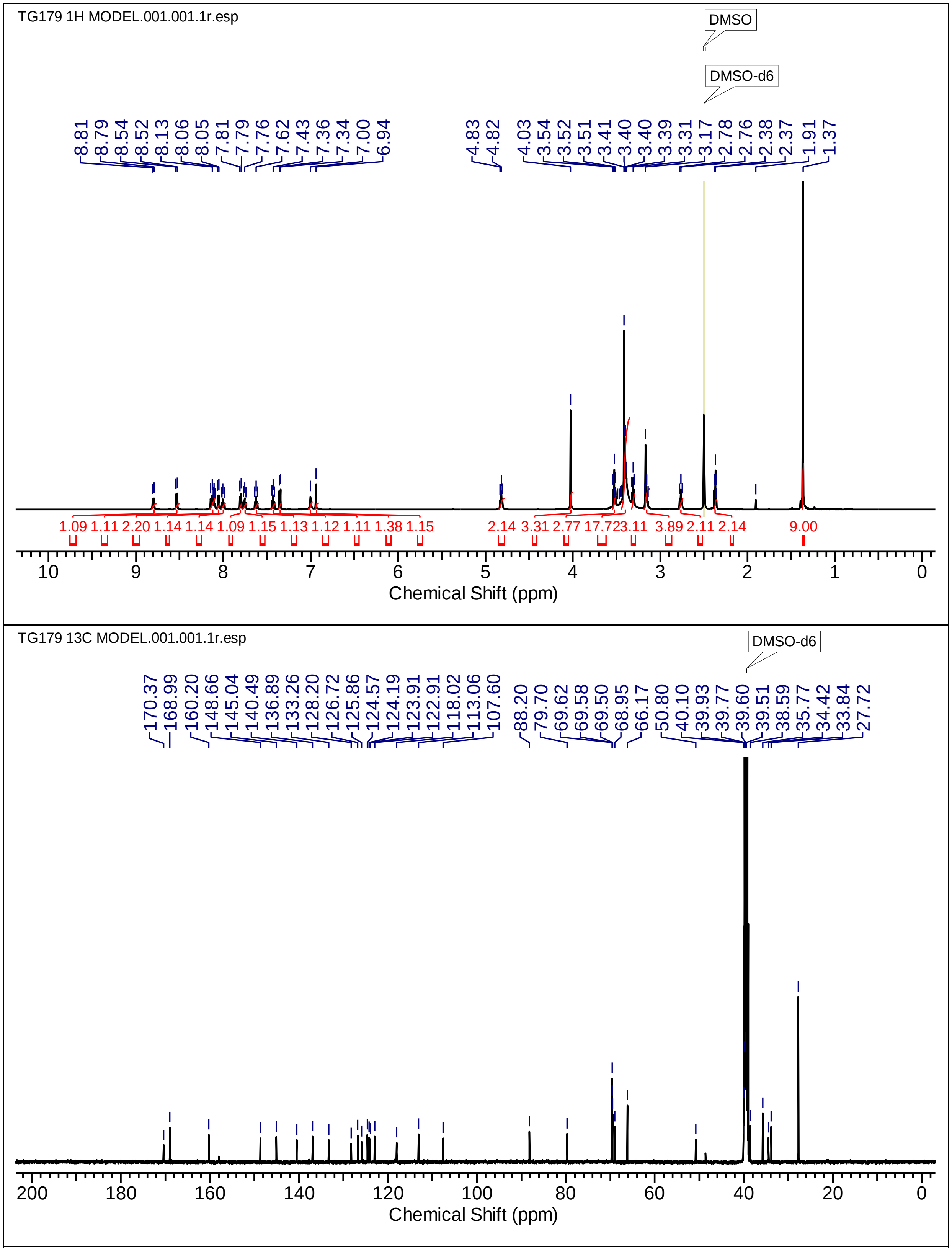


$^{1}H$ and $^{13}C$ NMR of compound **C4**

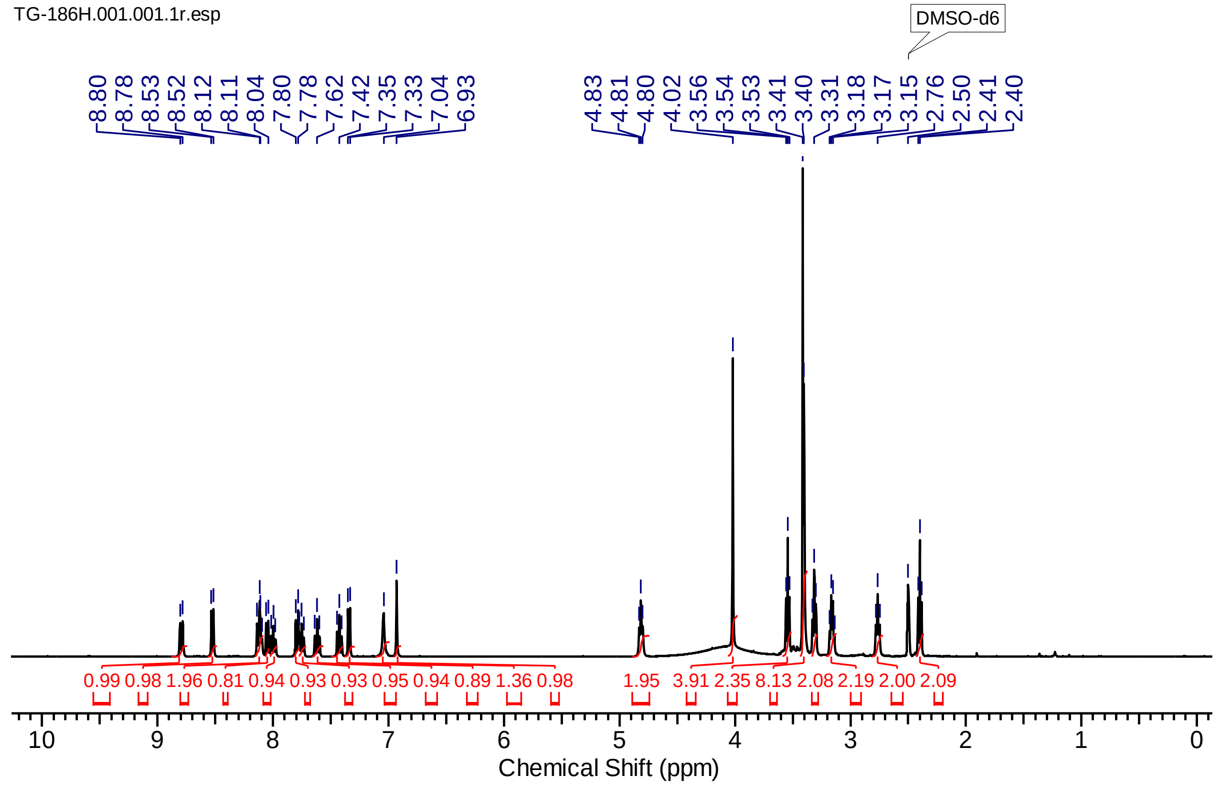


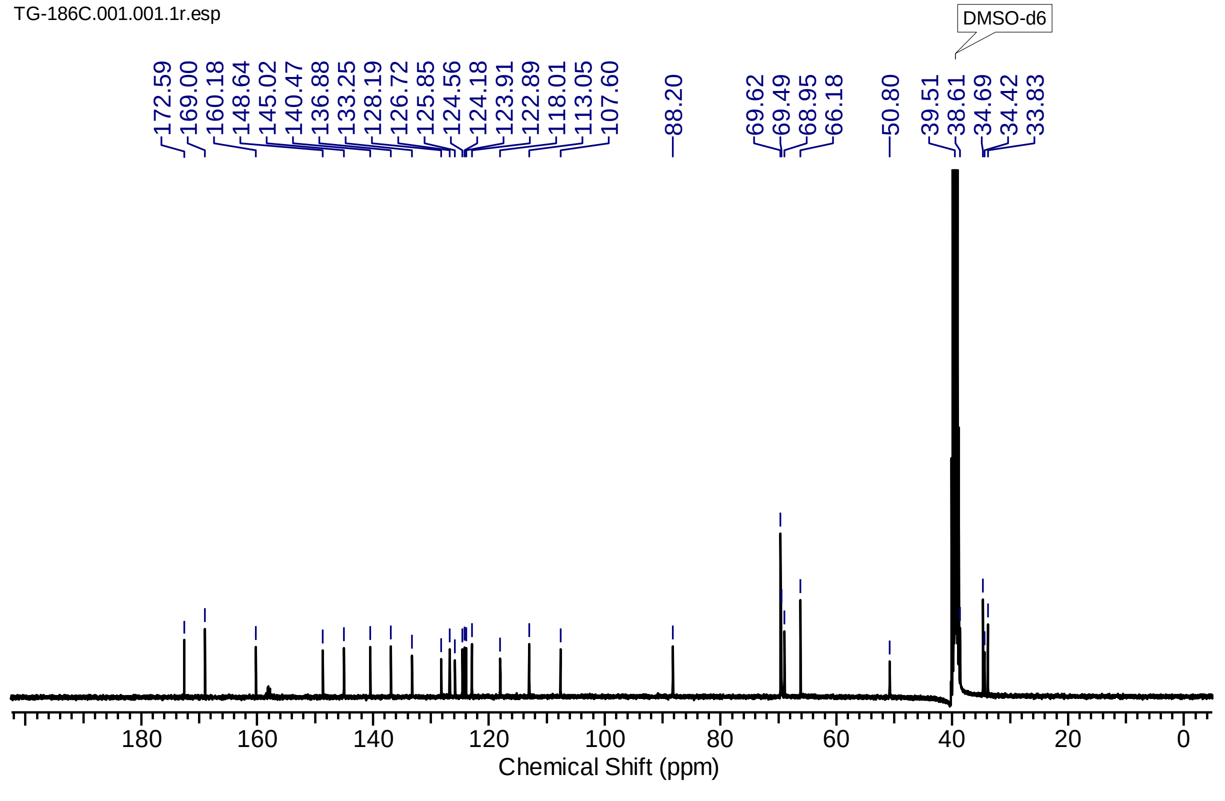


$^{1}$H and $^{13}$C NMR of compound **C5**

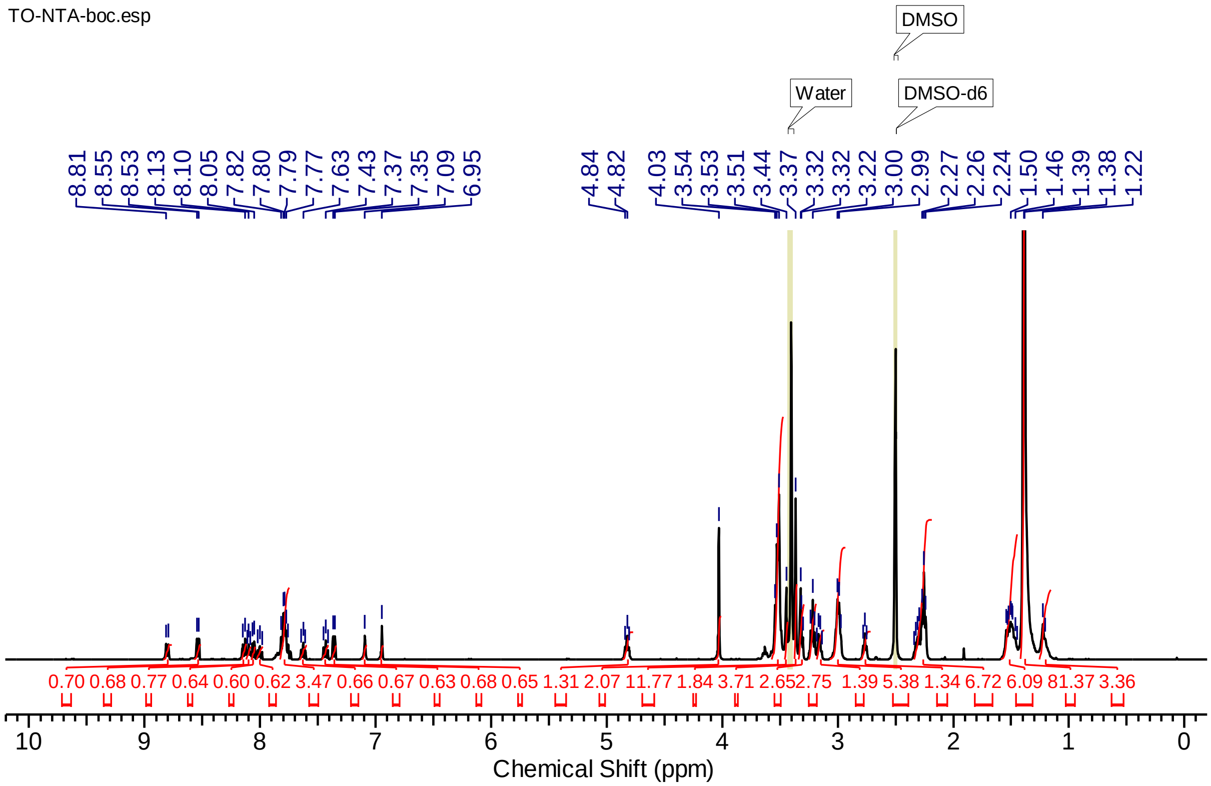


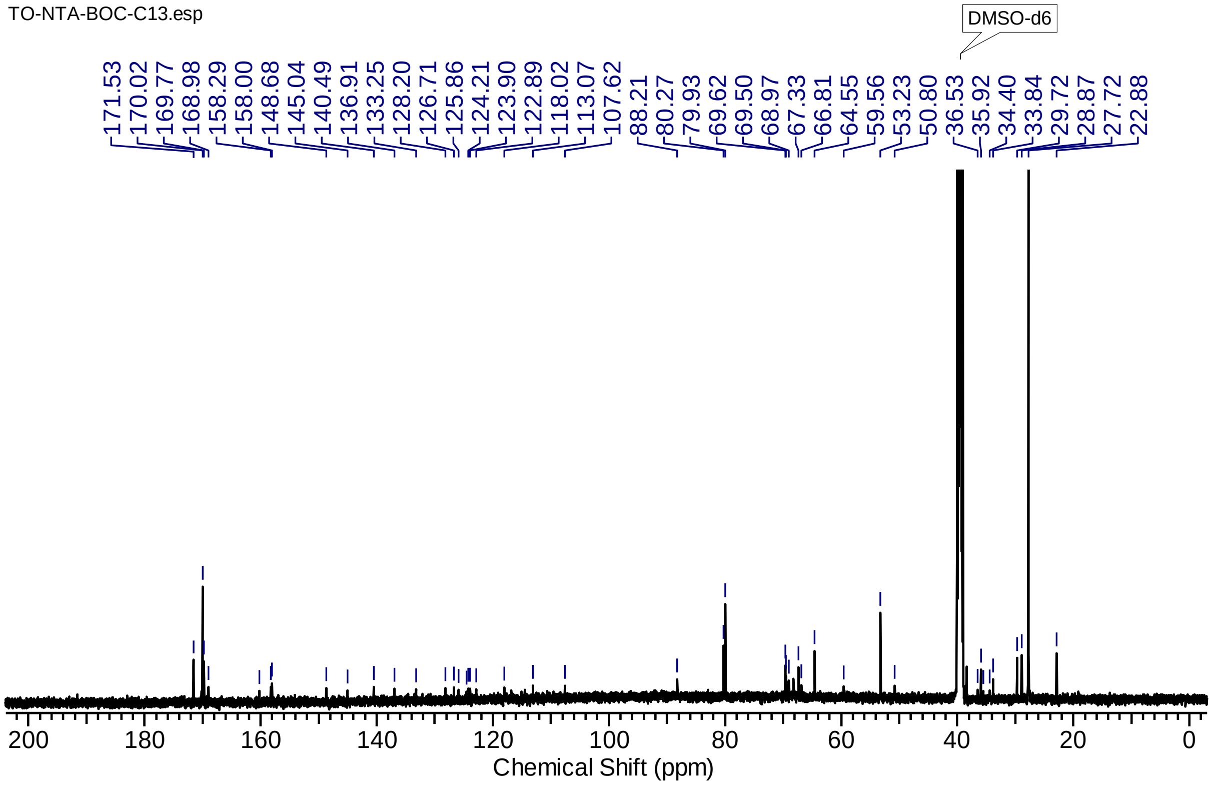


$^{1}$H and $^{13}$CNMR of compound **C6**

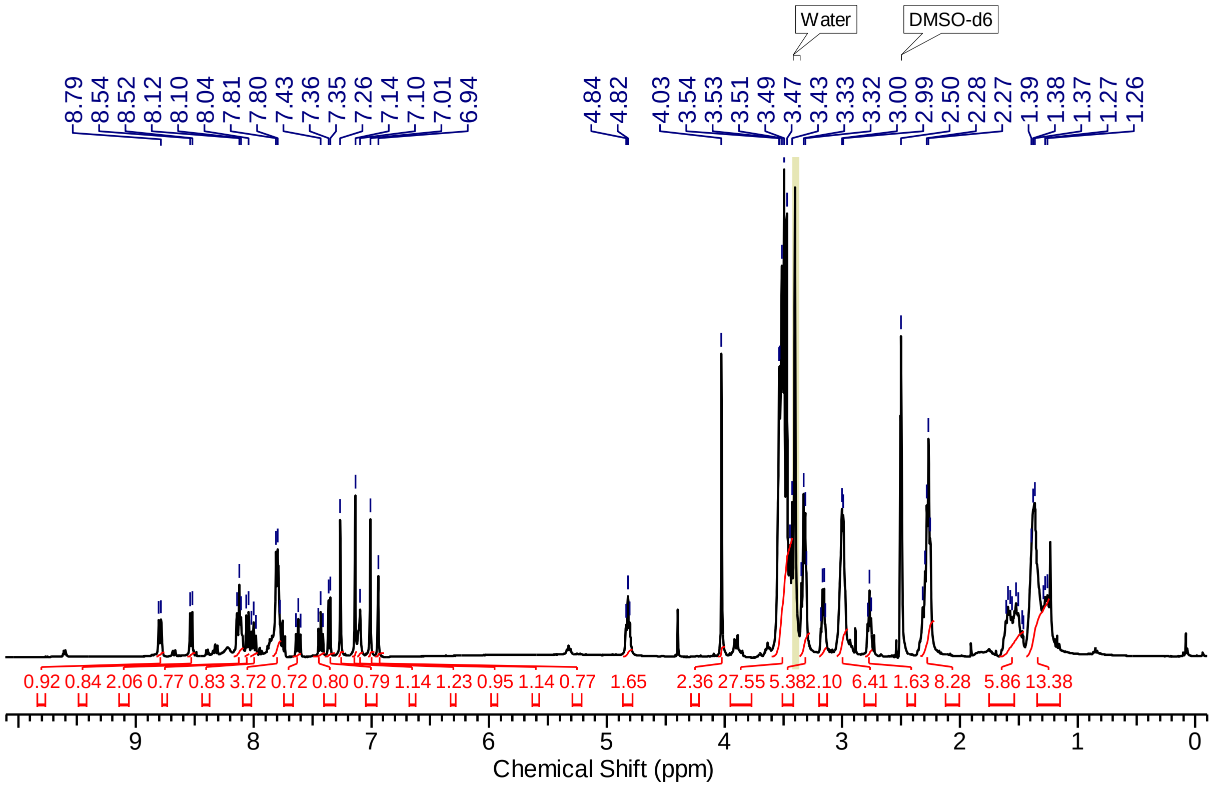


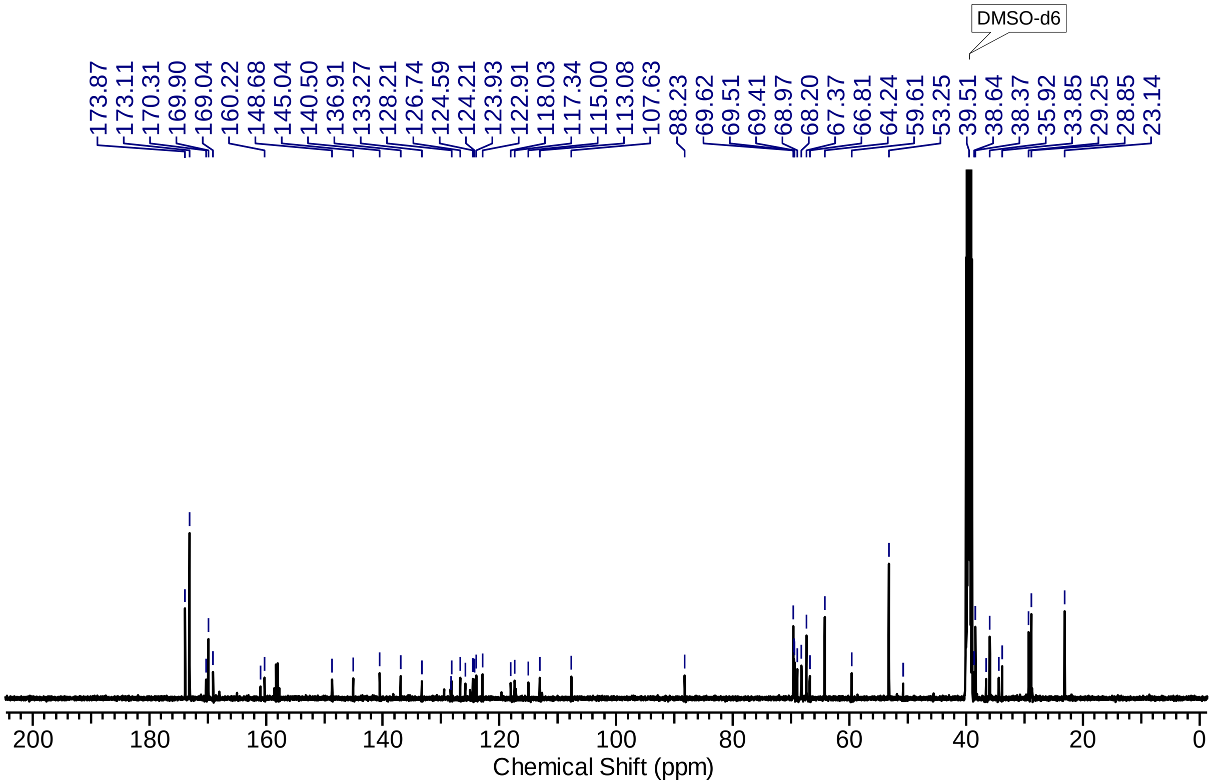


$^{1}$H and $^{13}$C NMR of compound **1**

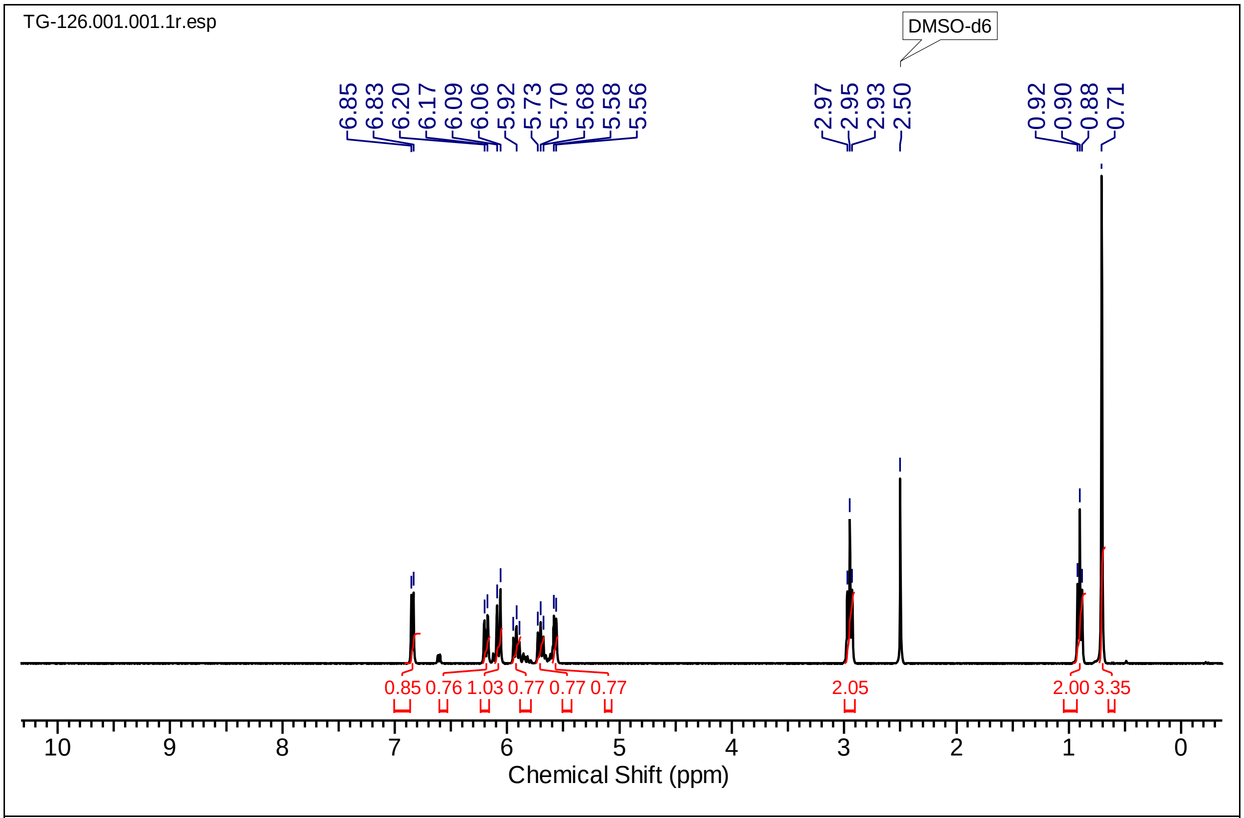


$^{1}$H and $^{13}$CNMR of compound **C7**

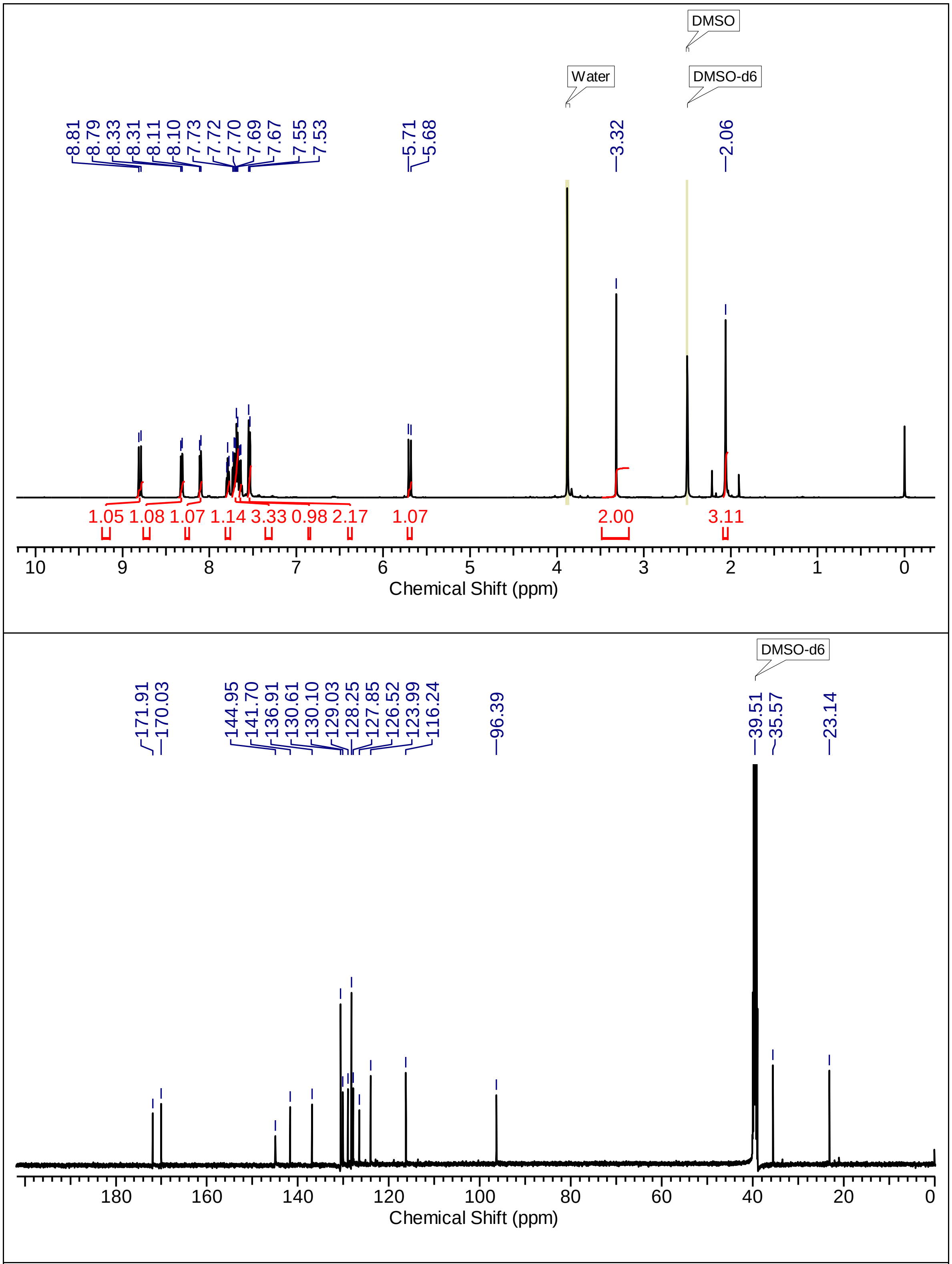


$^{1}$H and $^{13}$C NMR of compound **CP-7**

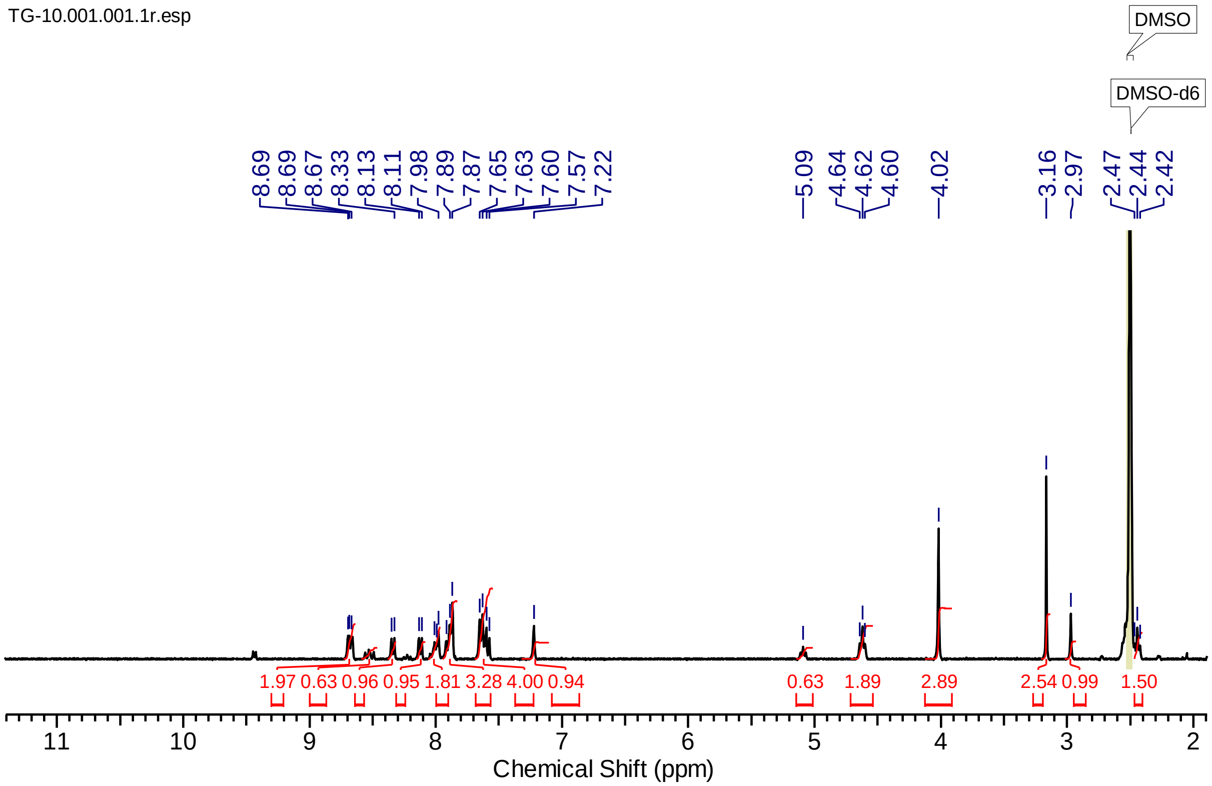


$^{1}$H NMR of compound **C8**

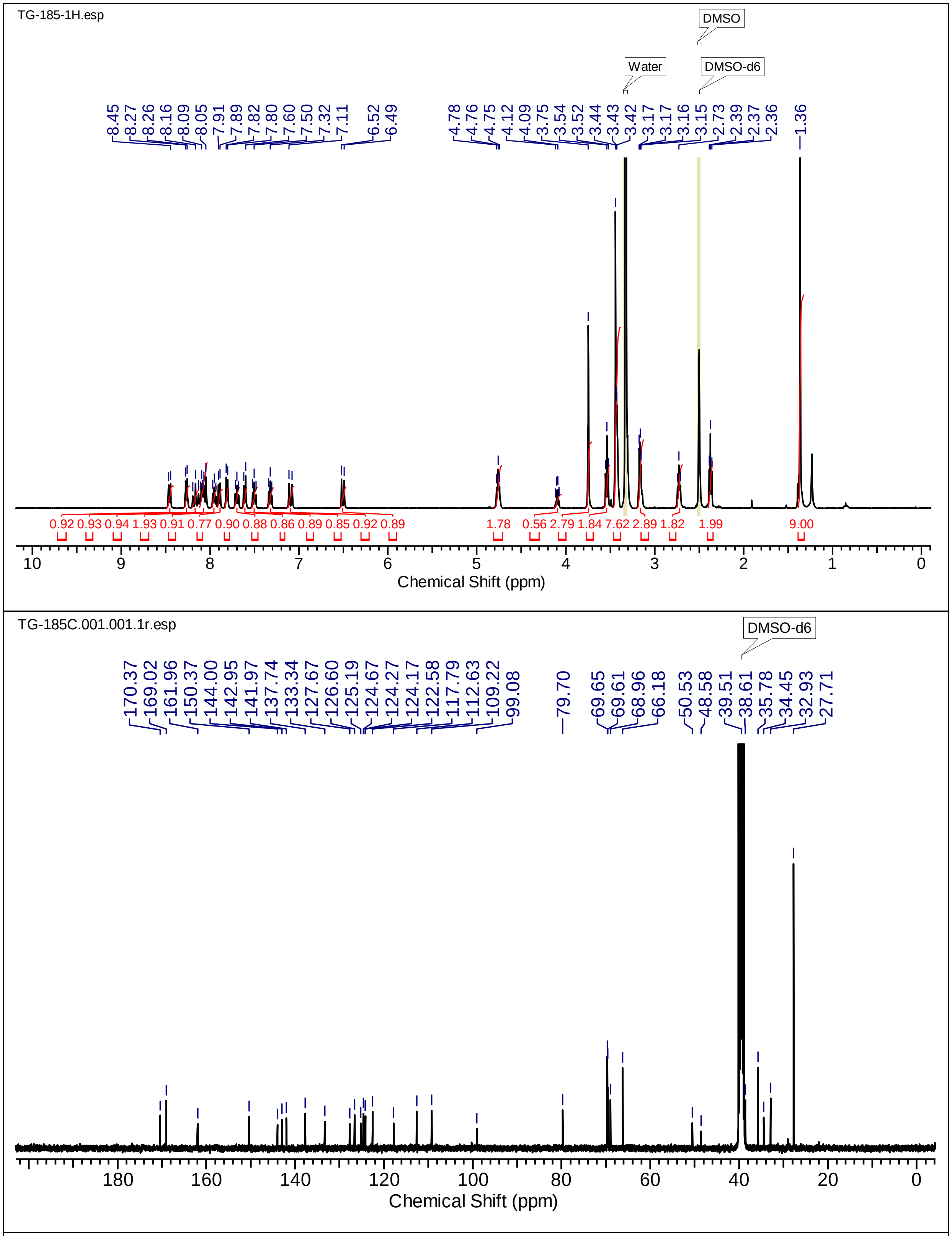


$^{1}$H NMR of compound **C9**

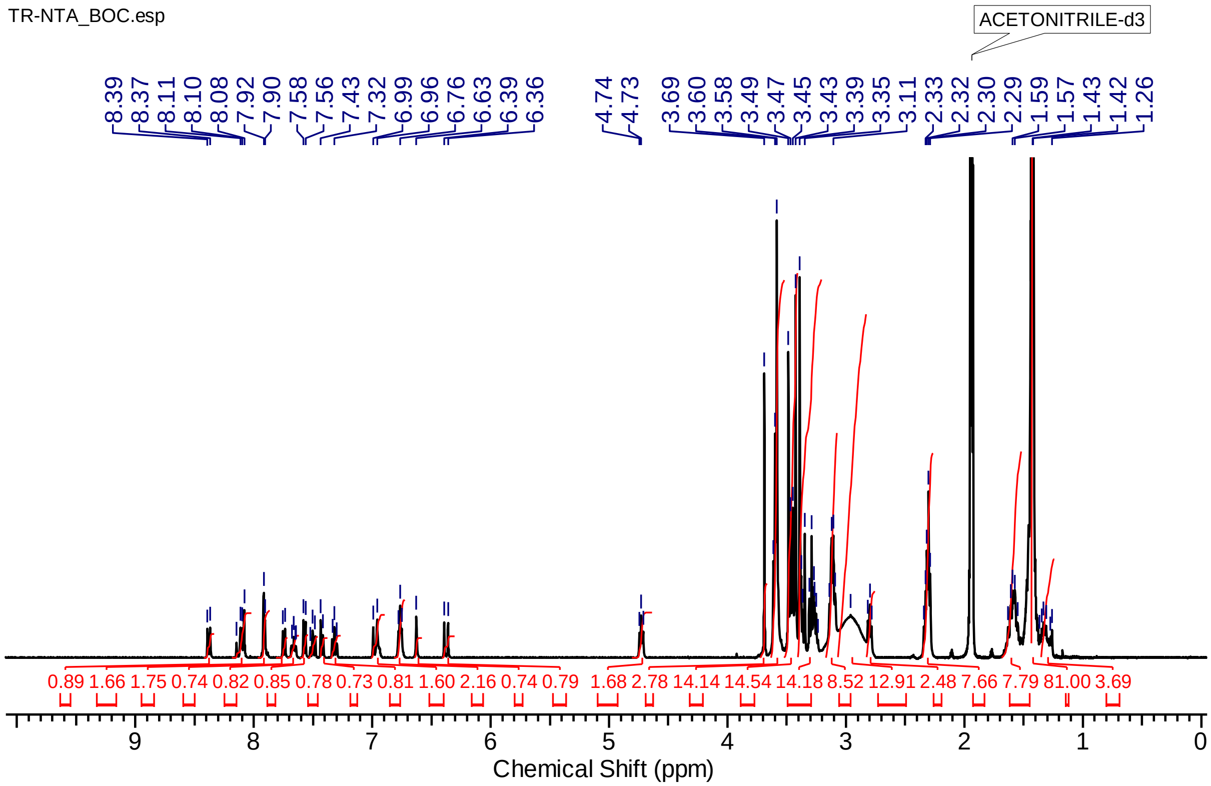


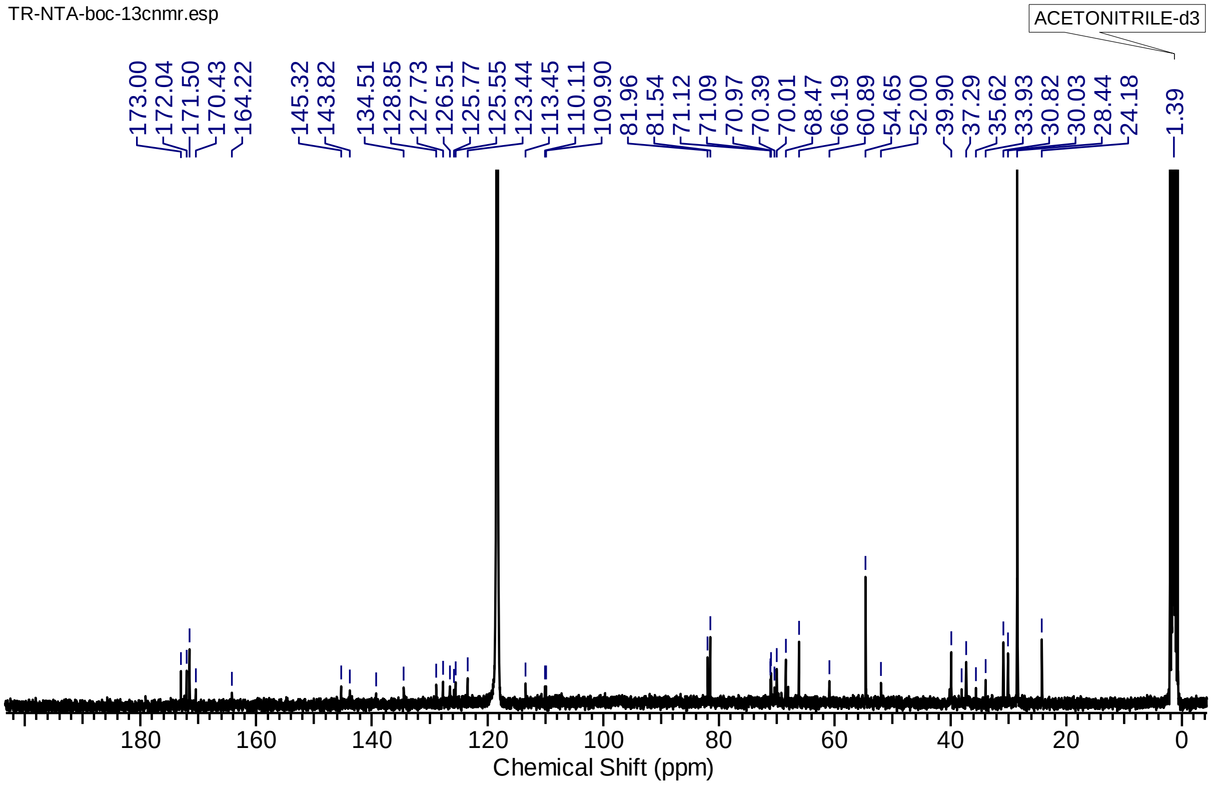


$^{1}$H and $^{13}$CNMR of compound **C10**

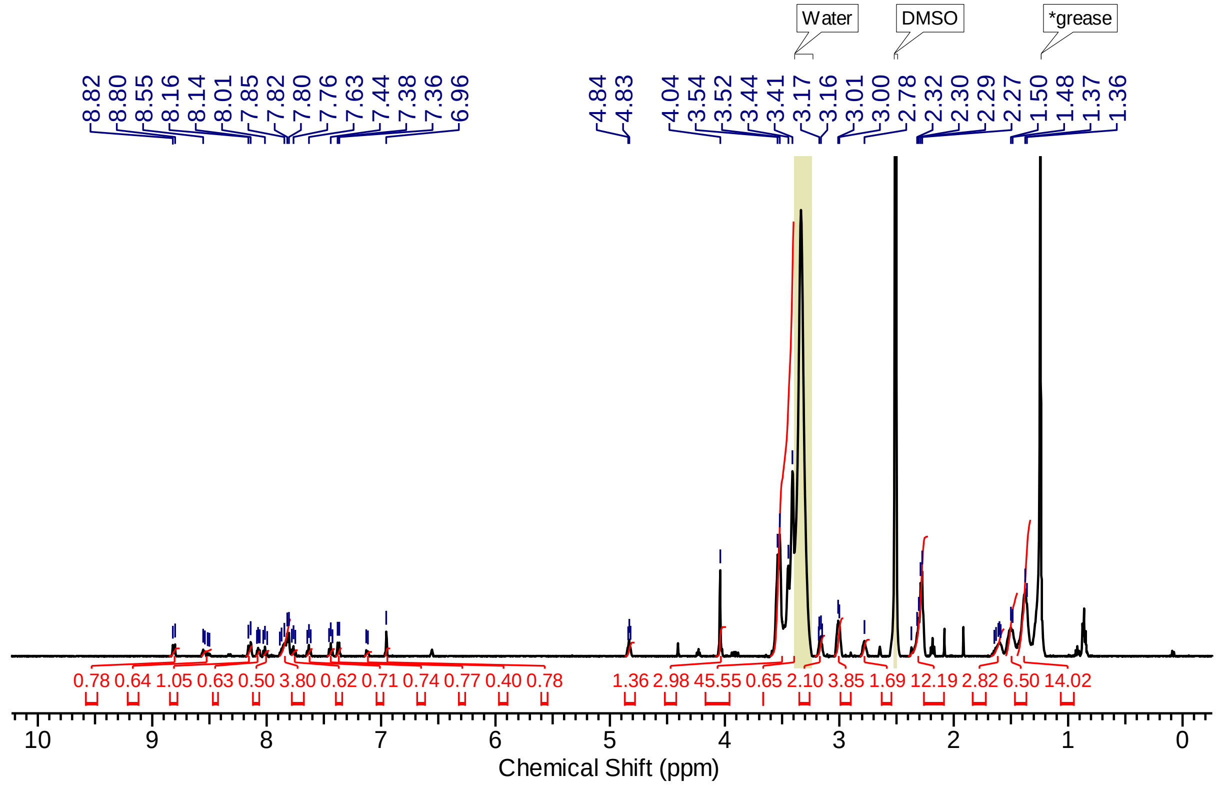


$^{1}H$ and $^{13}C$NMR of compound **2**

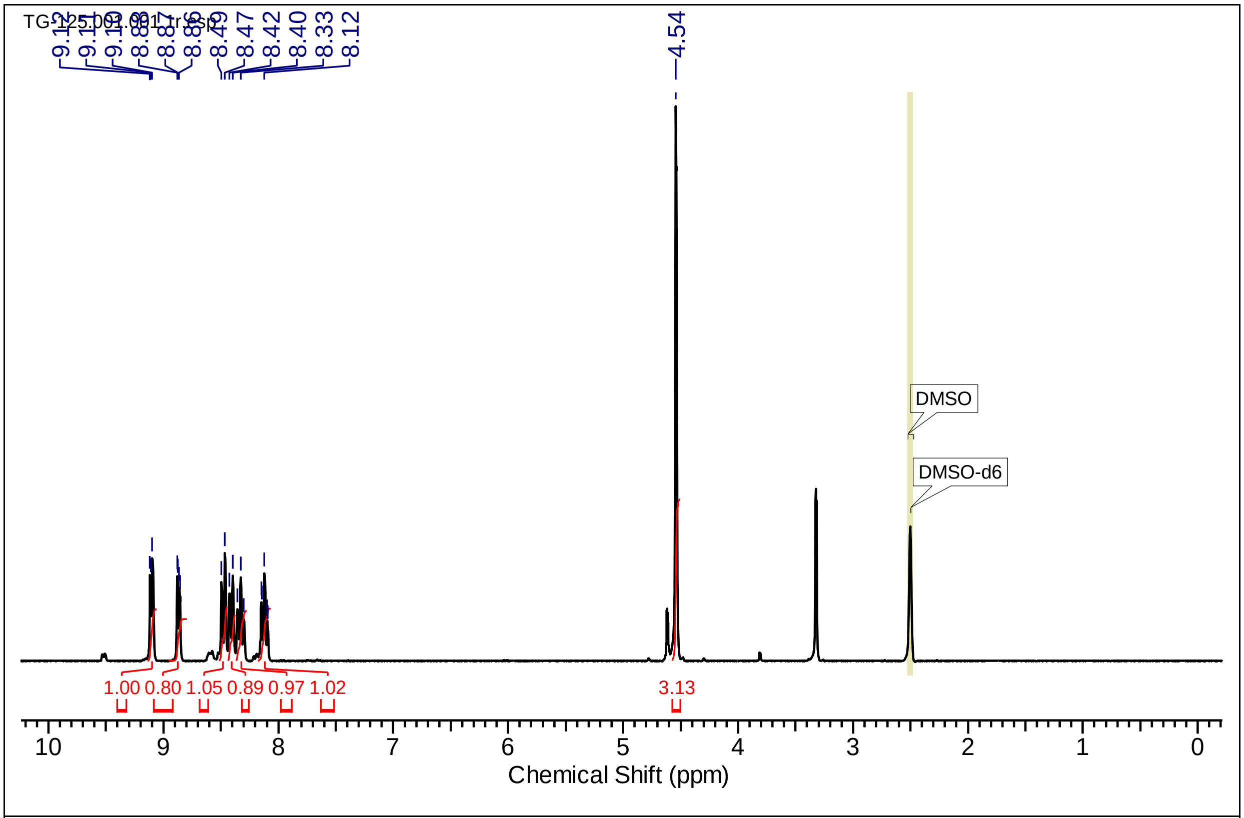


$^{1}$H NMR of compound **C11**

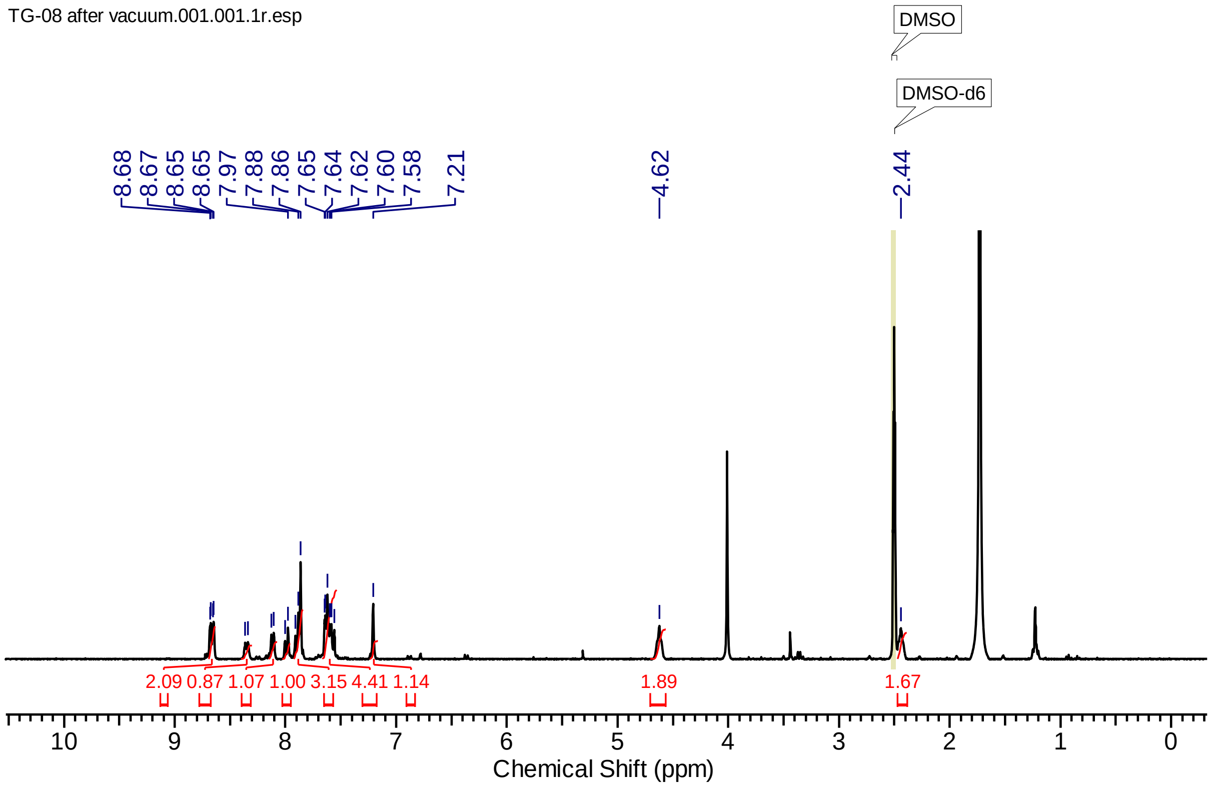


$^{1}$H NMR of compound **C12**

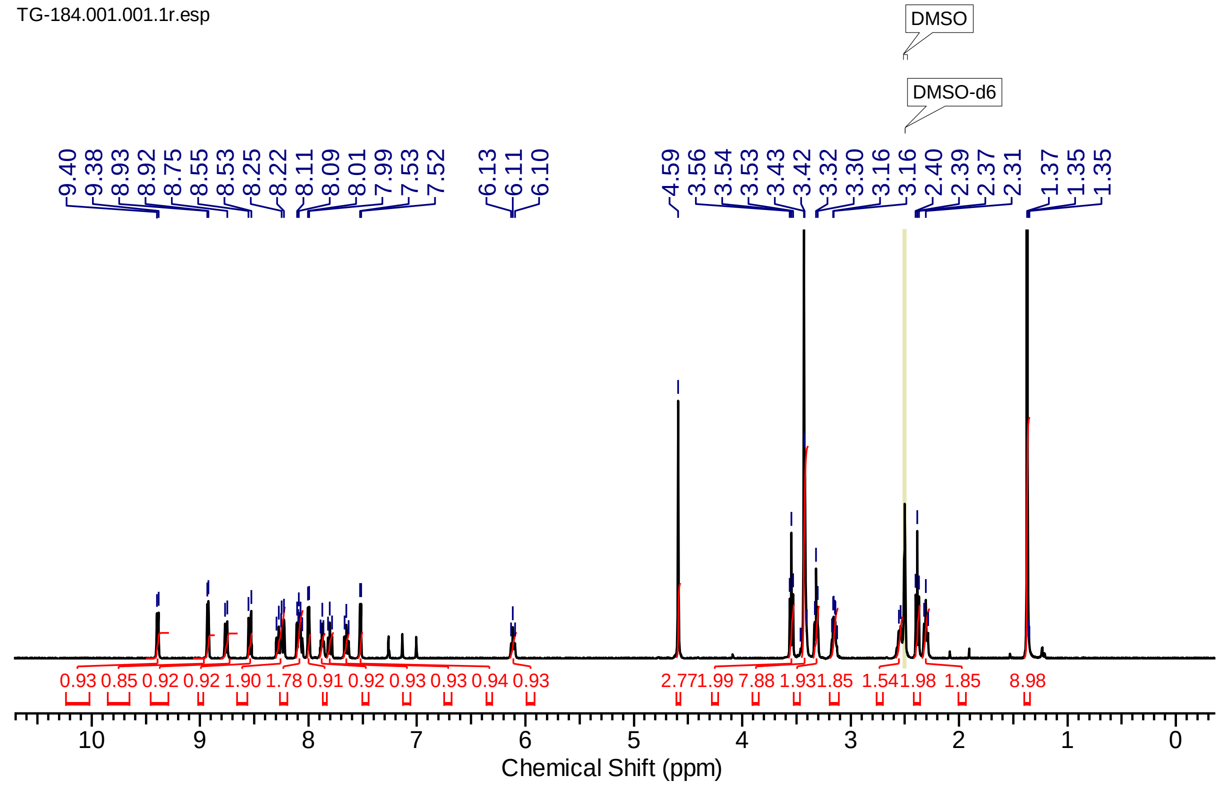


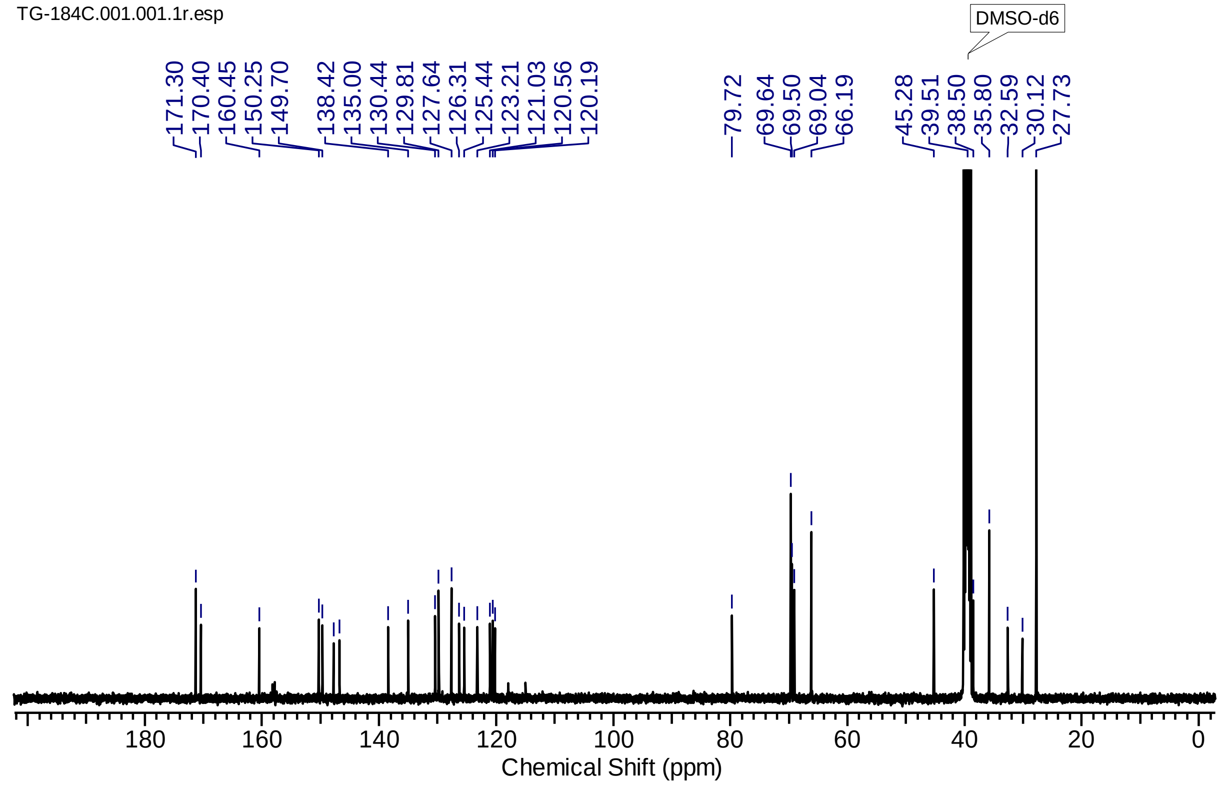


$^1$H and $^{13}$CNMR of compound **C13**

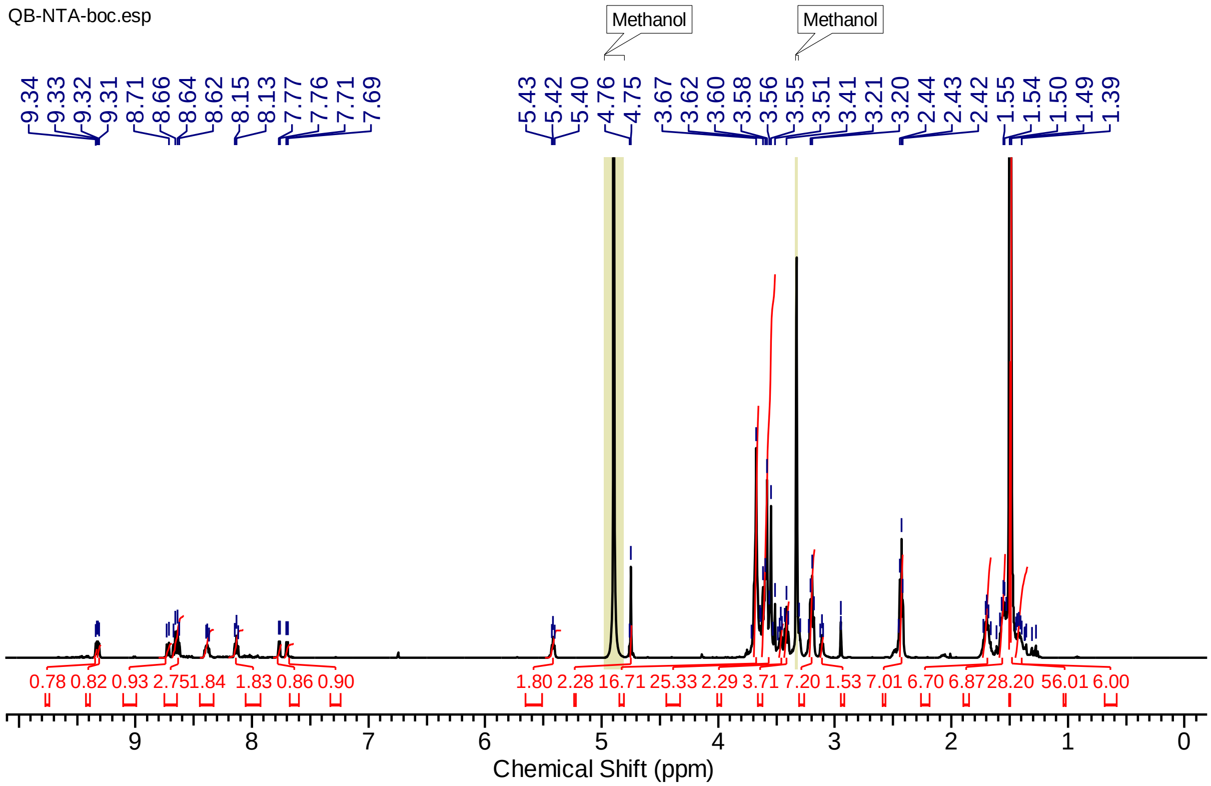


$^{1}$H NMR of compound **C14**

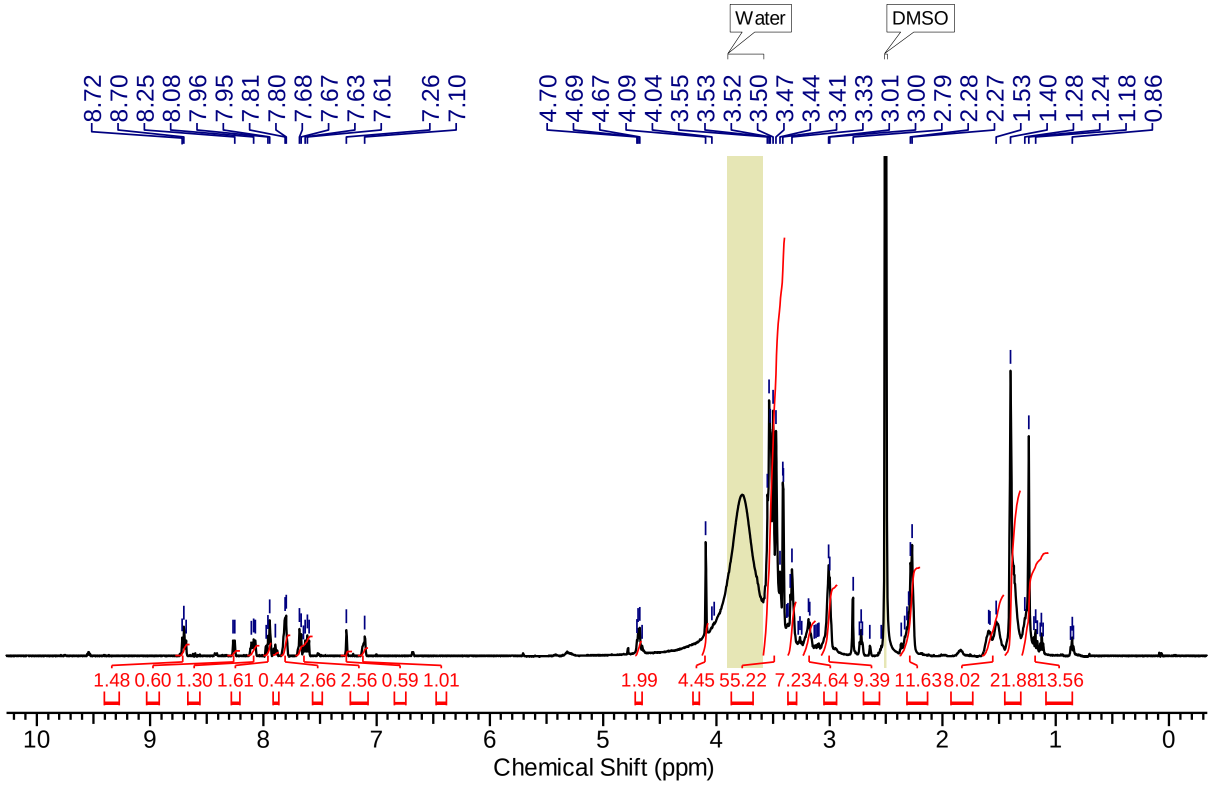


$^{1}H$ NMR of compound **3**

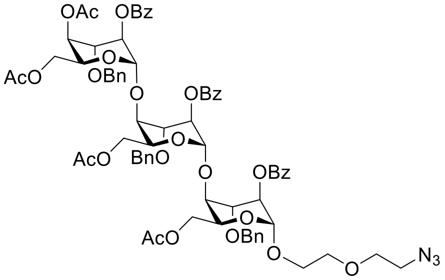

OAc
OBz
AcO
O
OBn
O
OBz
AcO
BnO
O
O
OBz
AcO
O
OBn
O
O
$N_3$


**28**

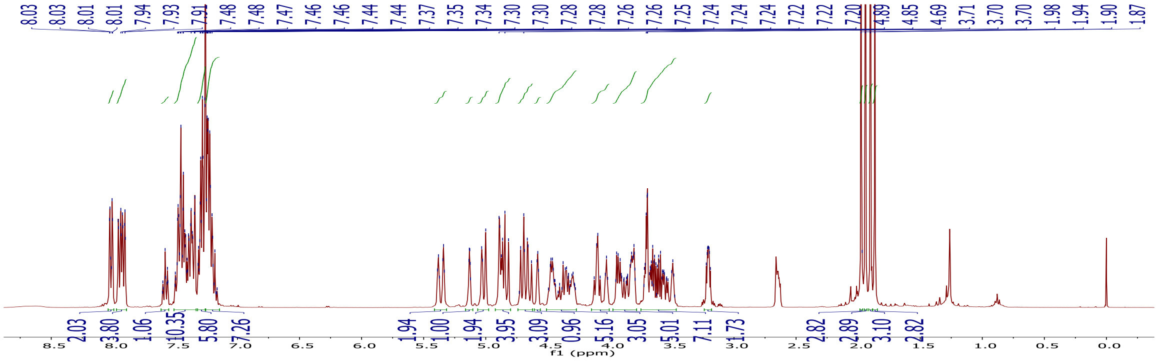

8.03 8.03 8.01 8.01 7.94 7.93 7.91 7.48 7.48 7.47 7.46 7.46 7.44 7.44 7.37 7.35 7.34 7.30 7.30 7.28 7.28 7.26 7.26 7.25 7.24 7.24 7.24 7.22 7.22 7.20 4.89 4.85 4.69 3.71 3.70 3.70 1.98 1.94 1.90 1.87
2.03 3.80 1.06 10.35 5.80 7.26 1.94 1.00 1.94 3.95 3.09 0.96 5.16 3.05 5.01 7.11 1.73 2.82 2.89 3.10 2.82
8.5 8.0 7.5 7.0 6.5 6.0 5.5 5.0 4.5 4.0 3.5 3.0 2.5 2.0 1.5 1.0 0.5 0.0
f1 (ppm)


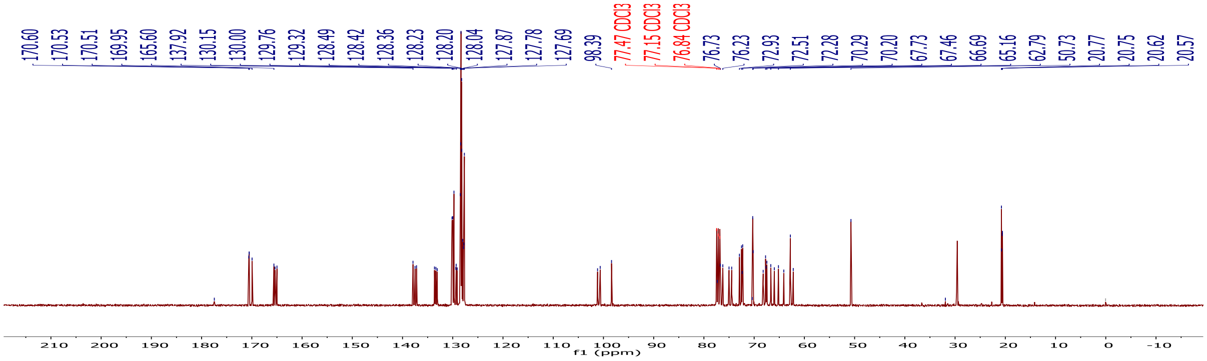

170.60 170.53 170.51 169.95 165.60 137.92 130.15 130.00 129.76 129.32 128.49 128.42 128.36 128.23 128.20 128.04 127.87 127.78 127.69 98.39
77.47 CDCl3
77.15 CDCl3
76.84 CDCl3
76.73 76.23 72.93 72.51 72.28 70.29 70.20 67.73 67.46 66.69 65.16 62.79 50.73 20.77 20.75 20.62 20.57
210 200 190 180 170 160 150 140 130 120 110 100 90 80 70 60 50 40 30 20 10 0 -10
f1 (ppm)


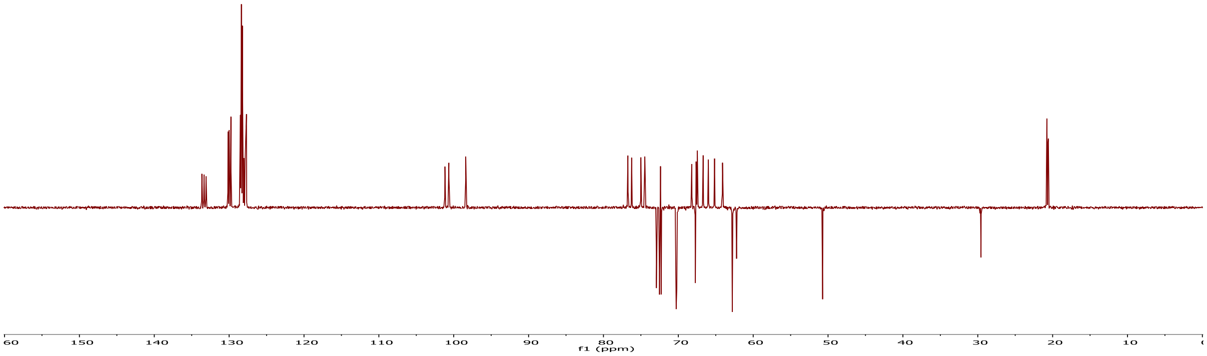

150 140 130 120 110 100 90 80 70 60 50 40 30 20 10
f1 (ppm)

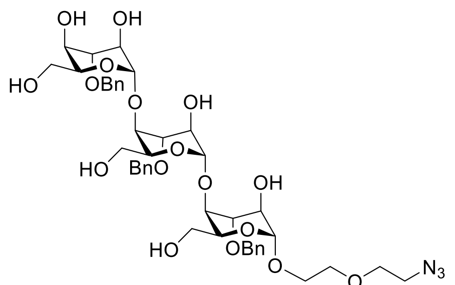
OH
OH
HO
O
OBn
O
OH
HO
BnO
O
O
OH
HO
O
OBn
O
O
N3

**29**

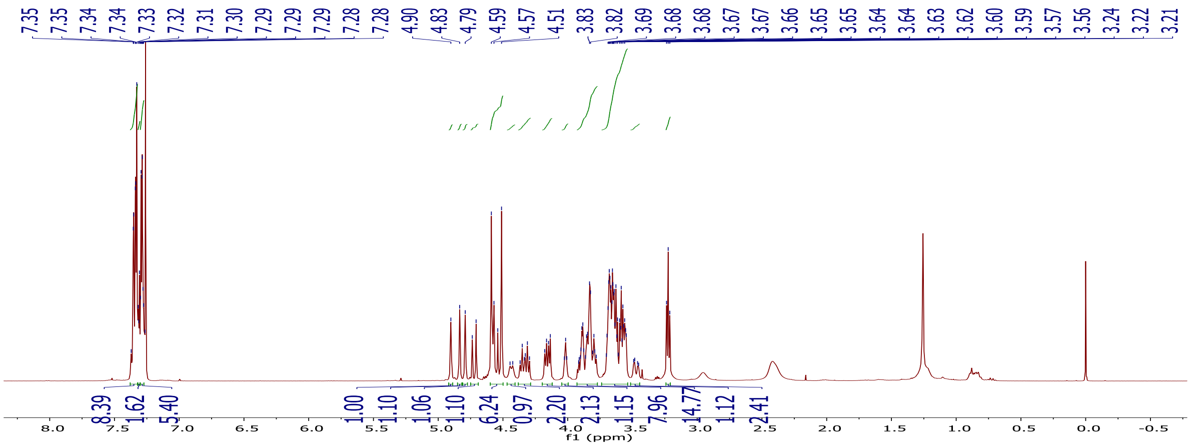
7.35 7.35 7.34 7.34 7.33 7.32 7.31 7.30 7.29 7.29 7.29 7.28 7.28 4.90 4.83 4.79 4.59 4.57 4.51 3.83 3.82 3.69 3.68 3.68 3.67 3.67 3.66 3.65 3.65 3.64 3.64 3.63 3.62 3.60 3.59 3.57 3.56 3.24 3.22 3.21
8.39 1.62 5.40 1.00 1.10 1.06 1.10 6.24 0.97 2.20 2.13 1.15 7.96 14.77 1.12 2.41
8.0 7.5 7.0 6.5 6.0 5.5 5.0 4.5 4.0 3.5 3.0 2.5 2.0 1.5 1.0 0.5 0.0 -0.5
f1 (ppm)

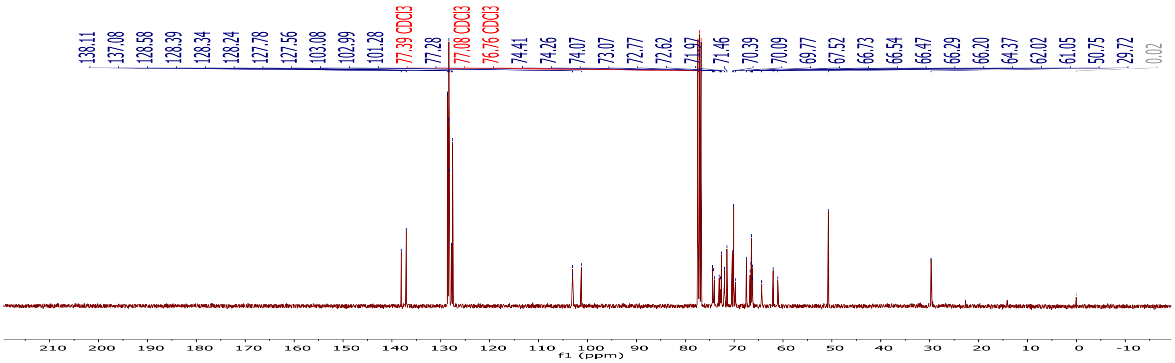
138.11 137.08 128.58 128.39 128.34 128.24 127.78 127.56 103.08 102.99 101.28 77.39 CDCl3 77.28 77.08 CDCl3 76.76 CDCl3 74.41 74.26 74.07 73.07 72.77 72.62 71.97 71.46 70.39 70.09 69.77 67.52 66.73 66.54 66.47 66.29 66.20 64.37 62.02 61.05 50.75 29.72 0.02
210 200 190 180 170 160 150 140 130 120 110 100 90 80 70 60 50 40 30 20 10 0 -10
f1 (ppm)

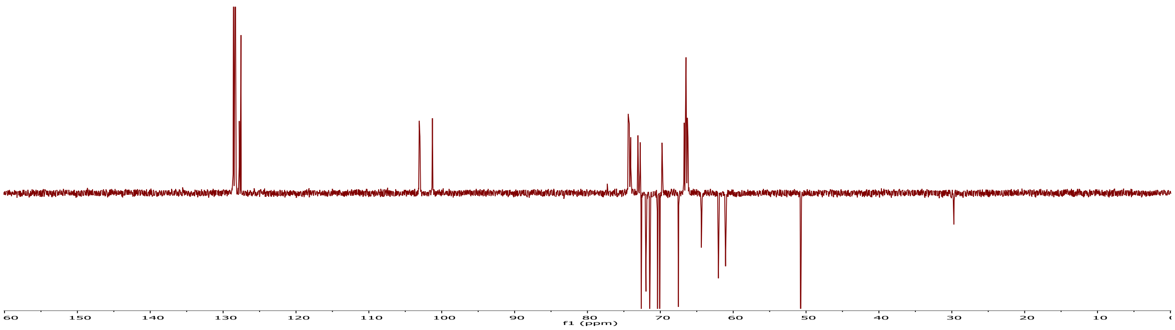
60 150 140 130 120 110 100 90 80 70 60 50 40 30 20 10
f1 (ppm)

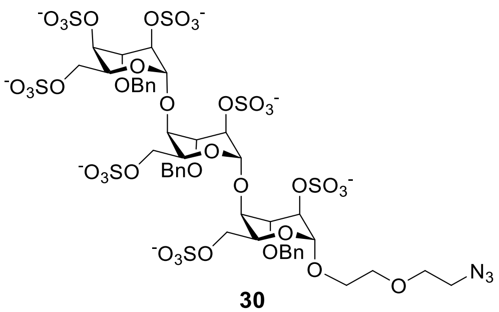

$^{-}O_3SO$
$OSO_3^{-}$
$^{-}O_3SO$
O
OBn
O
$OSO_3^{-}$
$^{-}O_3SO$
BnO
O
O
$OSO_3^{-}$
$^{-}O_3SO$
O
OBn
O
O
$N_3$


**30**

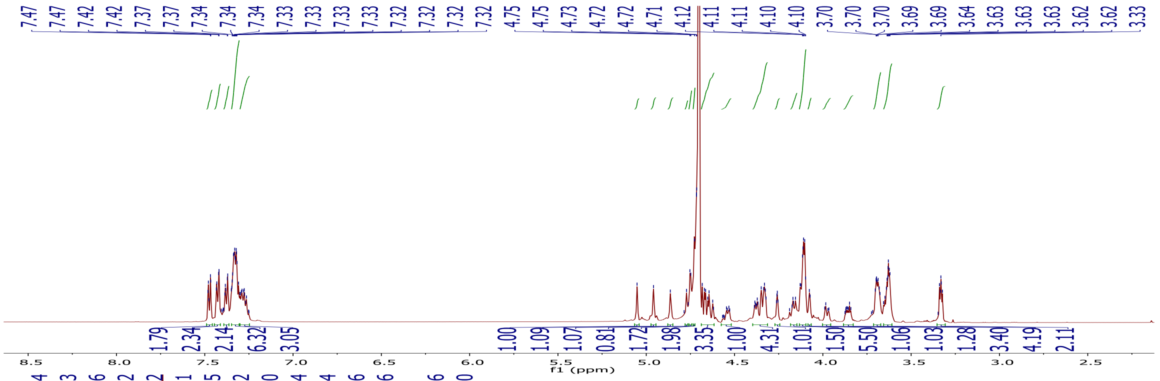

7.47 7.47 7.42 7.42 7.37 7.37 7.34 7.34 7.34 7.33 7.33 7.33 7.33 7.32 7.32 7.32 7.32
4.75 4.75 4.73 4.72 4.72 4.71 4.12 4.11 4.11 4.10 4.10
3.70 3.70 3.70 3.69 3.69 3.64 3.63 3.63 3.63 3.63 3.62 3.62 3.33
1.79 2.34 2.14 6.32 3.05
1.00 1.09 1.07 0.81 1.72 1.98 3.35 1.00 4.31 1.01 1.50 5.50 1.06 1.03 1.28 3.40 4.19 2.11
f1 (ppm)


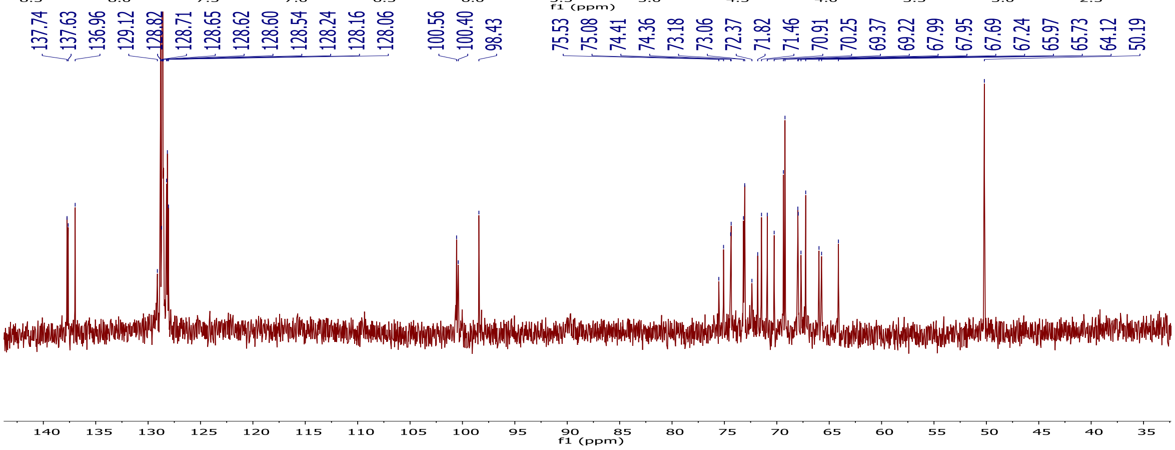

137.74 137.63 136.96 129.12 128.82 128.71 128.65 128.62 128.60 128.54 128.24 128.16 128.06
100.56 100.40 98.43
75.53 75.08 74.41 74.36 73.18 73.06 72.37 71.82 71.46 70.91 70.25 69.37 69.22 67.99 67.95 67.69 67.24 65.97 65.73 64.12 50.19
f1 (ppm)


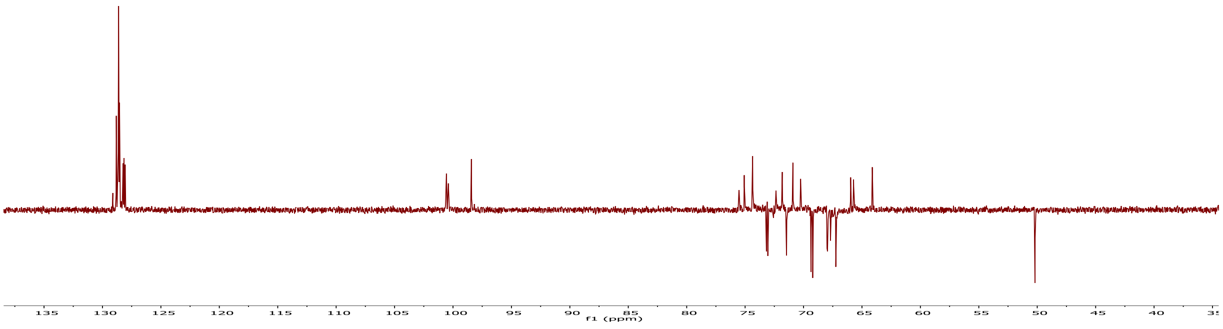

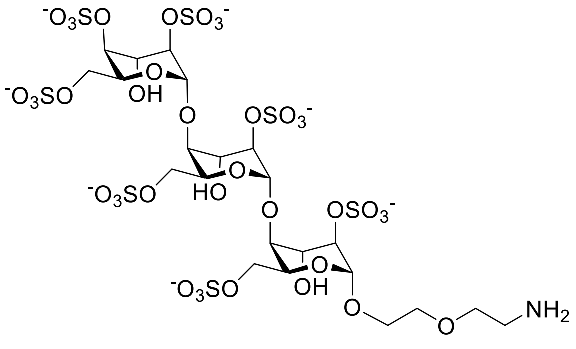

**HSA-9**

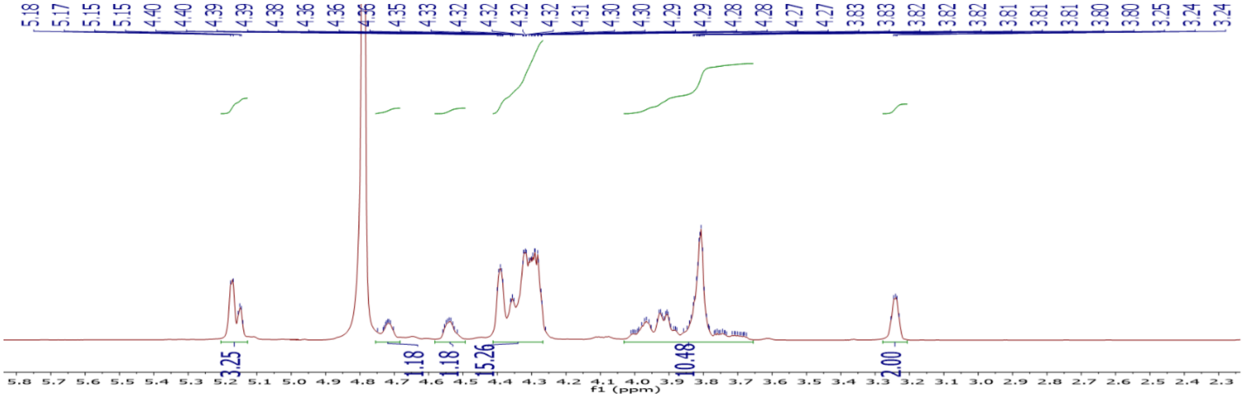
5.18 5.17 5.15 5.15 4.40 4.40 4.39 4.39 4.38 4.36 4.36 4.36 4.35 4.33 4.32 4.32 4.32 4.32 4.32 4.31 4.30 4.30 4.29 4.29 4.28 4.28 4.27 4.27 3.83 3.83 3.82 3.82 3.82 3.81 3.81 3.81 3.80 3.80 3.25 3.24 3.24
3.25
1.18
1.18
15.26
10.48
2.00
f1 (ppm)

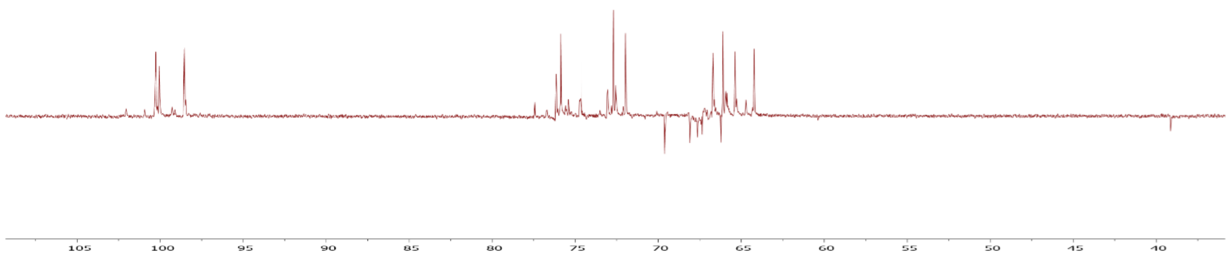

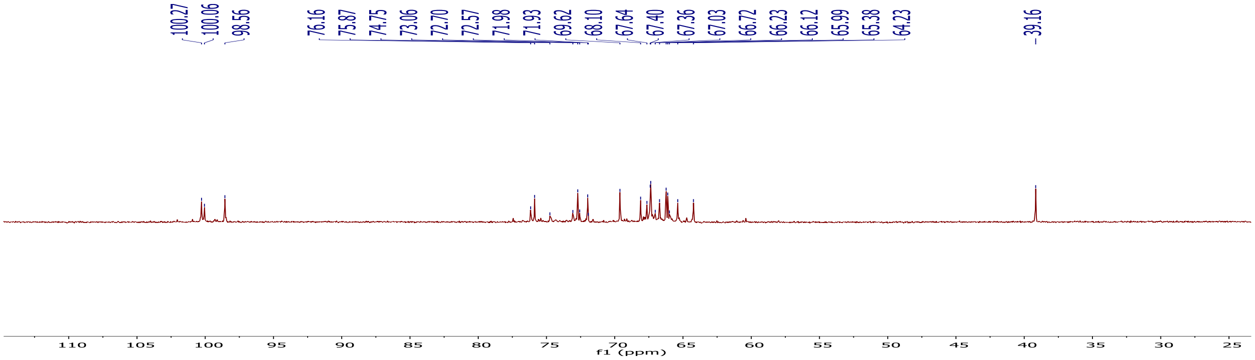
100.27 100.06 98.56
76.16 75.87 74.75 73.06 72.70 72.57 71.98 71.93 69.62 68.10 67.64 67.40 67.36 67.03 66.72 66.23 66.12 65.99 65.38 64.23
39.16
f1 (ppm)

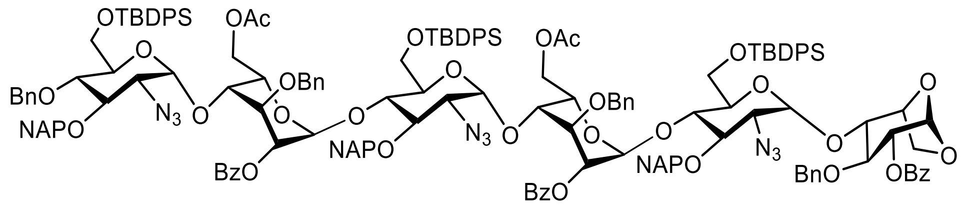
OTBDPS
OAc
OTBDPS
OAc
OTBDPS
BnO
NAPO
N3
OBn
BzO
NAPO
N3
OBn
BzO
NAPO
N3
BnO
OBz
O

**16**

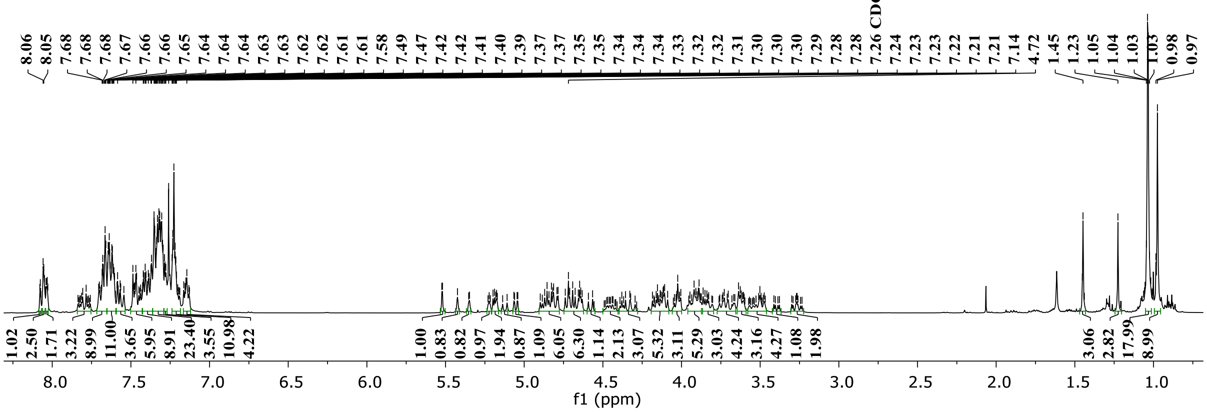
8.06 8.05 7.68 7.68 7.68 7.67 7.66 7.66 7.65 7.64 7.64 7.64 7.63 7.63 7.62 7.62 7.61 7.61 7.58 7.49 7.47 7.42 7.42 7.41 7.40 7.39 7.37 7.37 7.35 7.35 7.34 7.34 7.33 7.32 7.32 7.31 7.30 7.30 7.30 7.29 7.28 7.28 7.26 CDCl3 7.24 7.24 7.23 7.23 7.22 7.21 7.21 7.14 4.72 1.45 1.23 1.05 1.04 1.03 1.03 0.98 0.97
1.02 2.50 1.71 3.22 8.99 11.00 3.65 5.95 8.91 23.40 3.55 10.98 4.22 1.00 0.83 0.82 0.97 1.94 0.87 1.09 6.05 6.30 1.14 2.13 3.07 5.32 3.11 5.29 3.03 4.24 3.16 4.27 1.08 1.98 3.06 2.82 17.99 8.99
8.0 7.5 7.0 6.5 6.0 5.5 5.0 4.5 4.0 3.5 3.0 2.5 2.0 1.5 1.0
f1 (ppm)

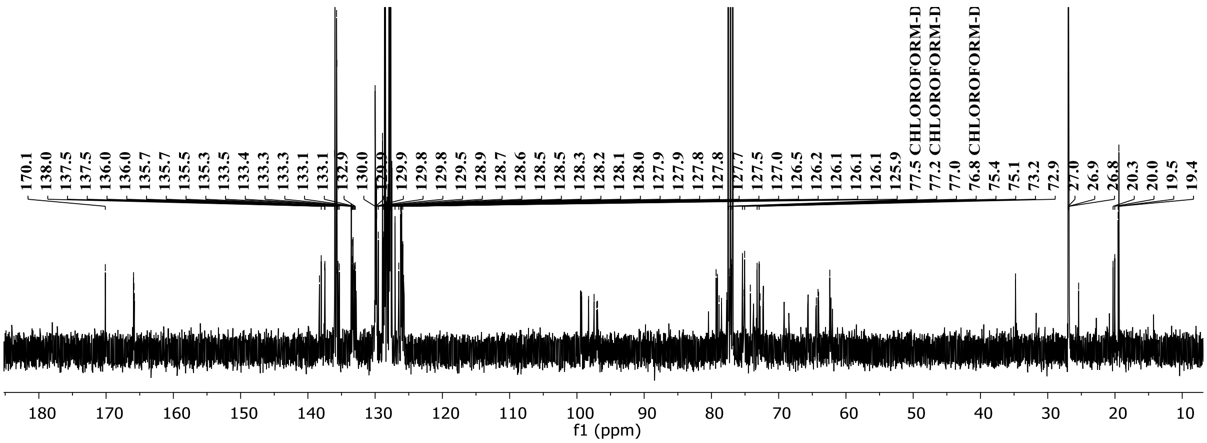
170.1 138.0 137.5 137.5 136.0 136.0 135.7 135.7 135.5 135.3 133.5 133.4 133.3 133.3 133.1 133.1 132.9 130.0 129.9 129.9 129.8 129.8 129.5 128.9 128.7 128.6 128.5 128.5 128.3 128.2 128.1 128.0 127.9 127.9 127.8 127.8 127.7 127.5 127.0 126.5 126.2 126.1 126.1 126.1 125.9 77.5 CHLOROFORM-D 77.2 CHLOROFORM-D 77.0 76.8 CHLOROFORM-D 75.4 75.1 73.2 72.9 27.0 26.9 26.8 20.3 20.0 19.5 19.4
180 170 160 150 140 130 120 110 100 90 80 70 60 50 40 30 20 10
f1 (ppm)

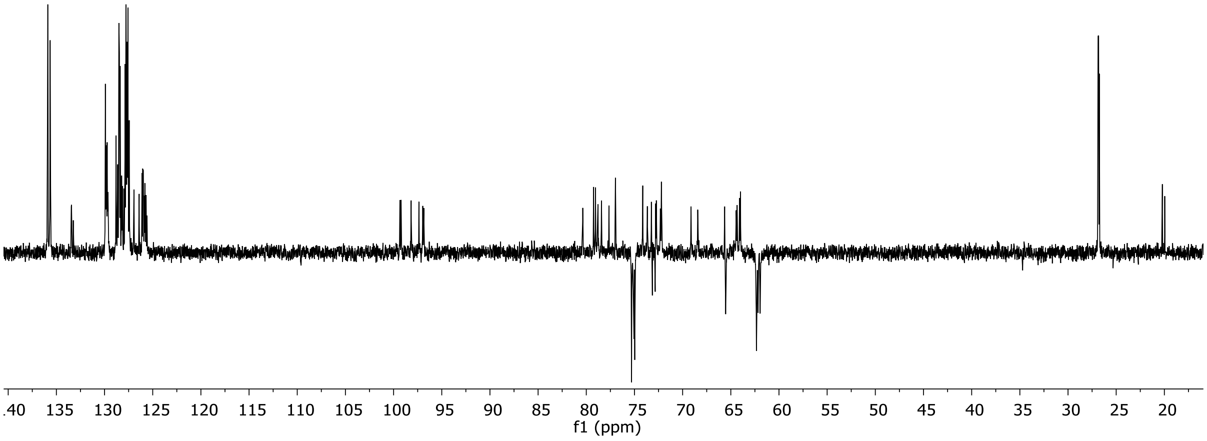
140 135 130 125 120 115 110 105 100 95 90 85 80 75 70 65 60 55 50 45 40 35 30 25 20
f1 (ppm)

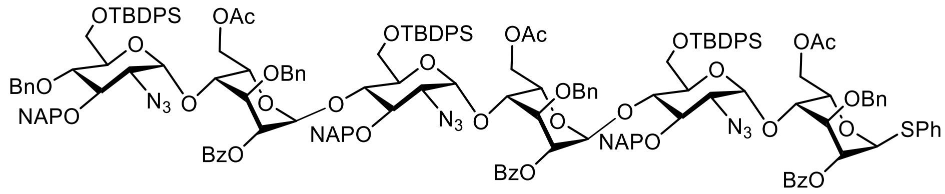
OTBDPS
OAc
BnO
NAPO
$N_3$
O
OBn
BzO
SPh

**17**

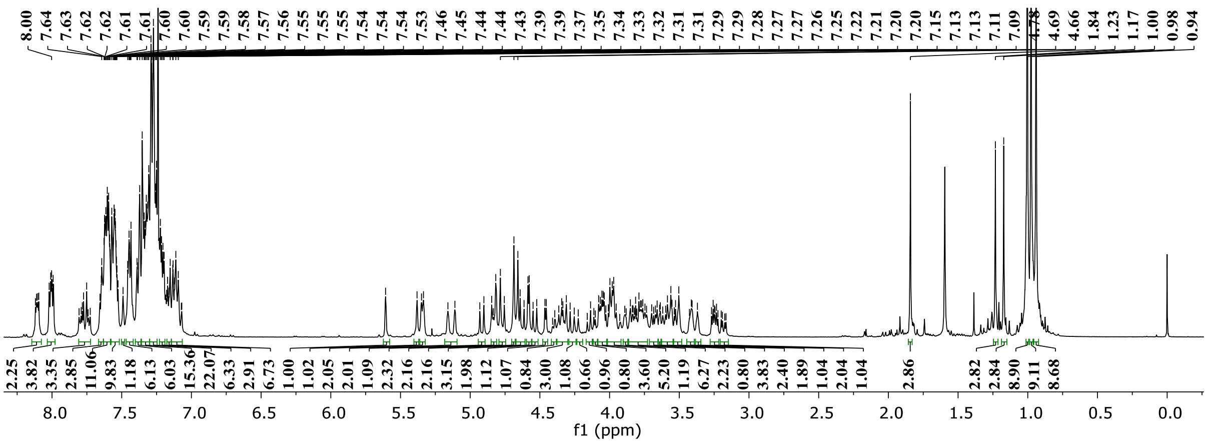
8.00
7.64
7.63
7.62
7.61
7.61
7.60
7.60
7.59
7.59
7.58
7.57
7.56
7.55
7.55
7.55
7.54
7.54
7.54
7.53
7.46
7.45
7.44
7.44
7.44
7.43
7.39
7.39
7.37
7.35
7.34
7.33
7.32
7.31
7.31
7.29
7.29
7.28
7.27
7.27
7.26
7.25
7.22
7.21
7.20
7.20
7.15
7.13
7.13
7.11
7.09
4.69
4.66
1.84
1.23
1.17
1.00
0.98
0.94
2.25
3.82
3.35
2.85
11.06
9.83
1.18
6.13
6.03
15.36
22.07
6.33
2.91
6.73
1.00
1.02
2.05
2.01
1.09
2.32
2.16
2.16
3.15
1.98
1.12
1.07
0.84
3.00
1.08
0.66
0.96
0.80
3.60
5.20
1.19
6.27
2.23
0.80
3.83
2.40
1.89
1.04
2.04
1.04
2.86
2.82
2.84
8.90
9.11
8.68
8.0 7.5 7.0 6.5 6.0 5.5 5.0 4.5 4.0 3.5 3.0 2.5 2.0 1.5 1.0 0.5 0.0
f1 (ppm)

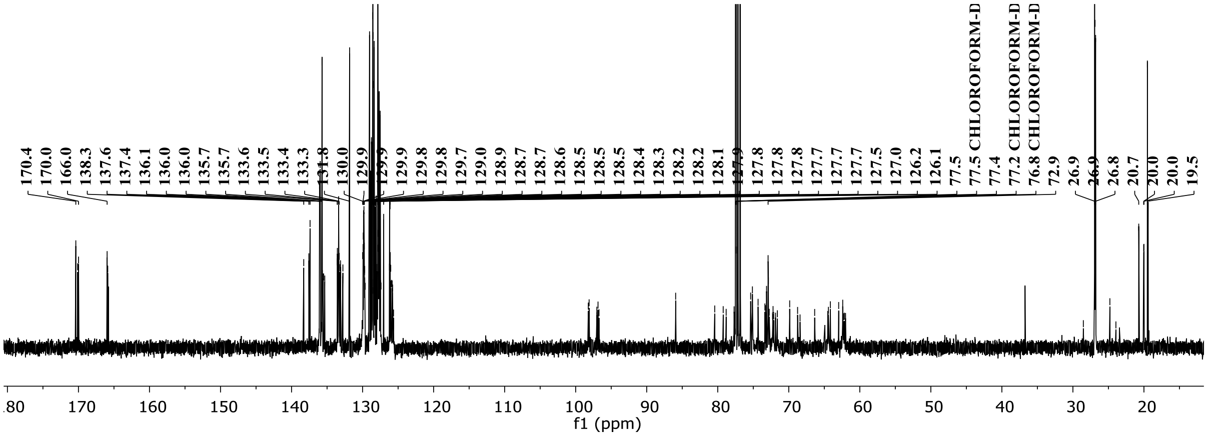
170.4
170.0
166.0
138.3
137.6
137.4
136.1
136.0
135.7
135.7
133.6
133.5
133.4
133.3
131.8
130.0
129.9
129.9
129.8
129.8
129.7
129.0
128.9
128.7
128.7
128.6
128.5
128.5
128.5
128.4
128.3
128.2
128.2
128.1
127.8
127.8
127.7
127.7
127.7
127.5
127.0
126.2
126.1
77.5
77.5 CHLOROFORM-D
77.4
77.2 CHLOROFORM-D
76.8 CHLOROFORM-D
72.9
26.9
26.9
26.8
20.7
20.0
20.0
19.5
.80 170 160 150 140 130 120 110 100 90 80 70 60 50 40 30 20
f1 (ppm)

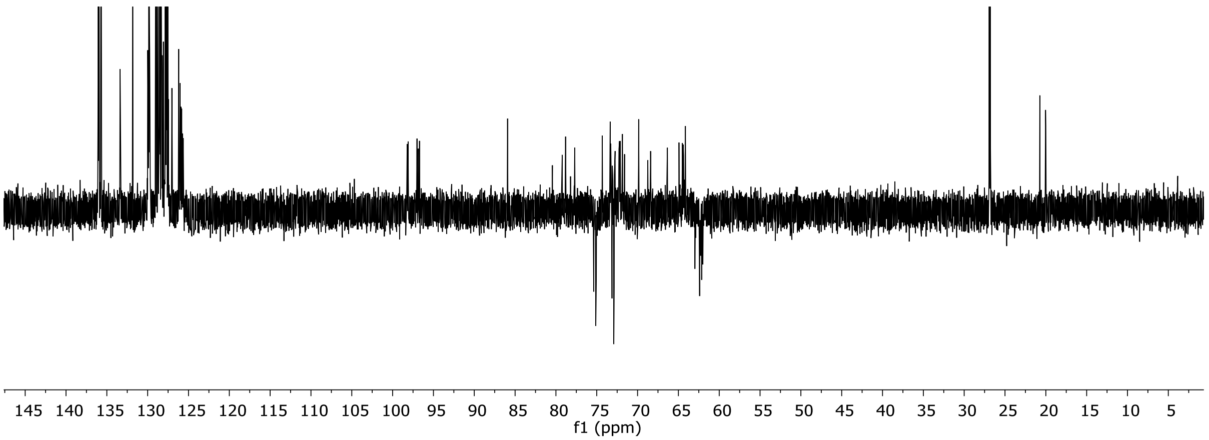
145 140 135 130 125 120 115 110 105 100 95 90 85 80 75 70 65 60 55 50 45 40 35 30 25 20 15 10 5
f1 (ppm)

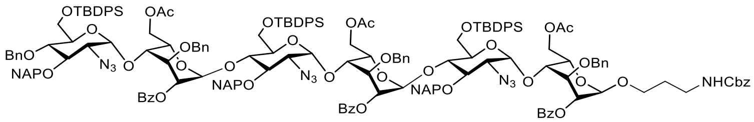
OTBDPS
OAc
BnO
NAPO
N3
OBn
BzO
NAPO
NHCbz


**18**

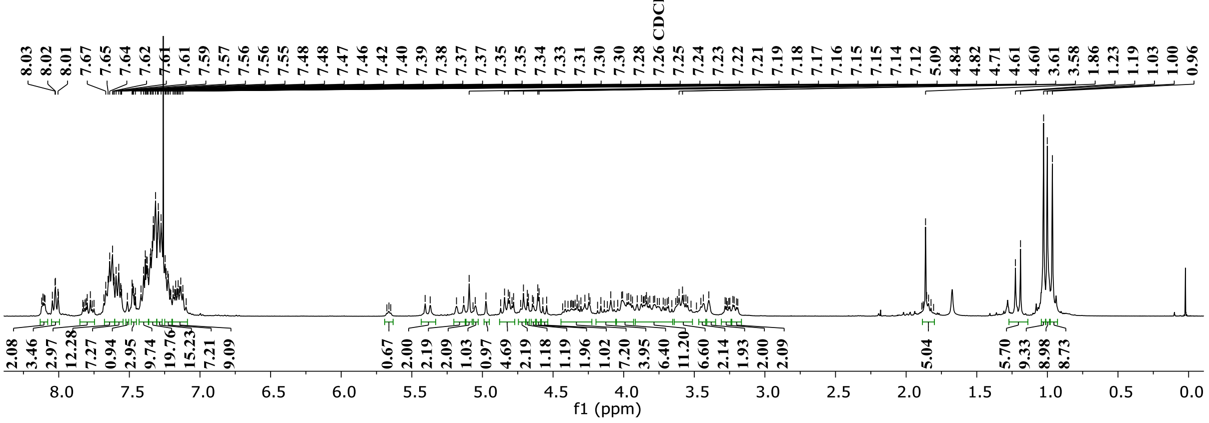
8.03 8.02 8.01 7.67 7.65 7.64 7.62 7.61 7.61 7.59 7.57 7.56 7.55 7.48 7.48 7.47 7.46 7.42 7.40 7.39 7.38 7.37 7.37 7.35 7.35 7.34 7.33 7.31 7.30 7.30 7.28 7.26 CDCl3 7.25 7.24 7.23 7.22 7.21 7.19 7.18 7.17 7.16 7.15 7.15 7.14 7.12 5.09 4.84 4.82 4.71 4.61 4.60 3.61 3.58 1.86 1.23 1.19 1.03 1.00 0.96
2.08 3.46 2.97 12.28 7.27 0.94 2.95 9.74 19.76 15.23 7.21 9.09 0.67 2.00 2.19 2.09 1.03 0.97 4.69 2.19 1.18 1.19 1.96 1.02 7.20 3.95 6.40 11.20 6.60 2.14 1.93 2.00 2.09 5.04 5.70 9.33 8.98 8.73
8.0 7.5 7.0 6.5 6.0 5.5 5.0 4.5 4.0 3.5 3.0 2.5 2.0 1.5 1.0 0.5 0.0
f1 (ppm)

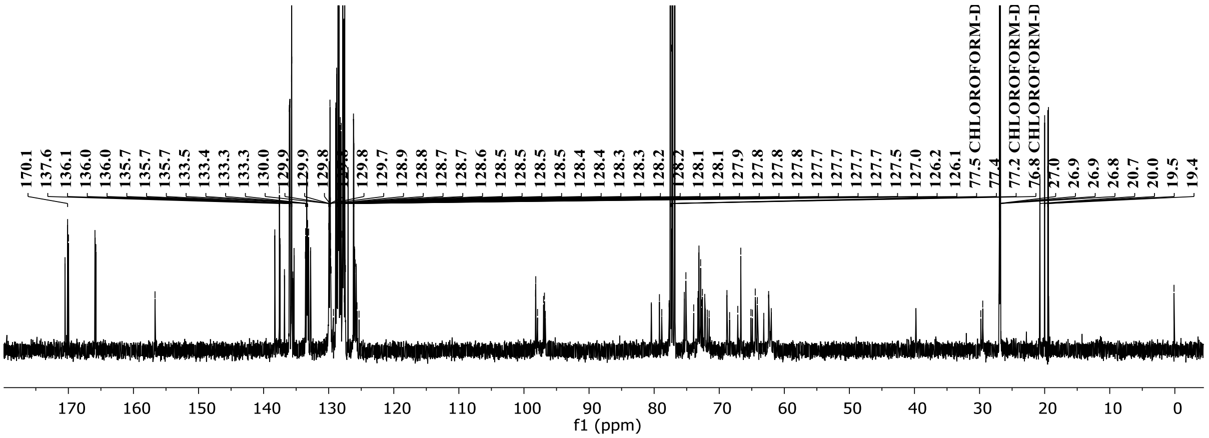
170.1 137.6 136.1 136.0 136.0 135.7 135.7 135.7 133.5 133.4 133.3 133.3 130.0 129.9 129.9 129.8 129.8 129.7 128.9 128.8 128.7 128.7 128.6 128.5 128.5 128.5 128.5 128.4 128.4 128.3 128.3 128.2 128.2 128.1 128.1 127.9 127.8 127.8 127.7 127.7 127.7 127.7 127.5 127.0 126.2 126.1 77.5 CHLOROFORM-D 77.4 77.2 CHLOROFORM-D 76.8 CHLOROFORM-D 27.0 26.9 26.9 26.8 20.7 20.0 19.5 19.4
170 160 150 140 130 120 110 100 90 80 70 60 50 40 30 20 10 0
f1 (ppm)

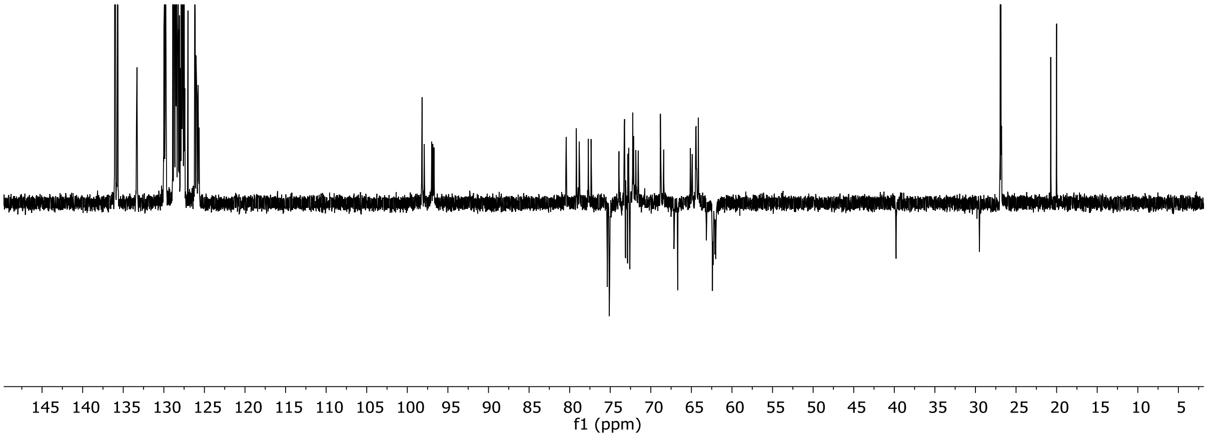
145 140 135 130 125 120 115 110 105 100 95 90 85 80 75 70 65 60 55 50 45 40 35 30 25 20 15 10 5
f1 (ppm)

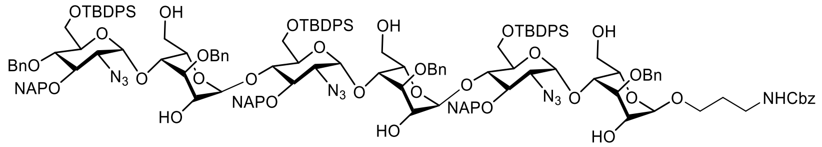
OTBDPS
OH
BnO
NAPO
N3
OBn
HO
NAPO
NHCbz

**19**

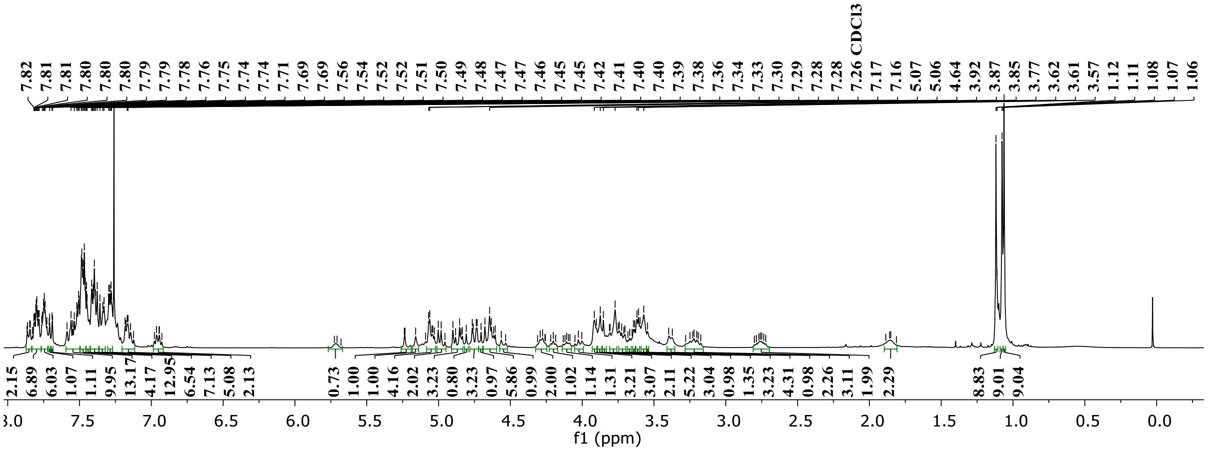
CDCl3
f1 (ppm)

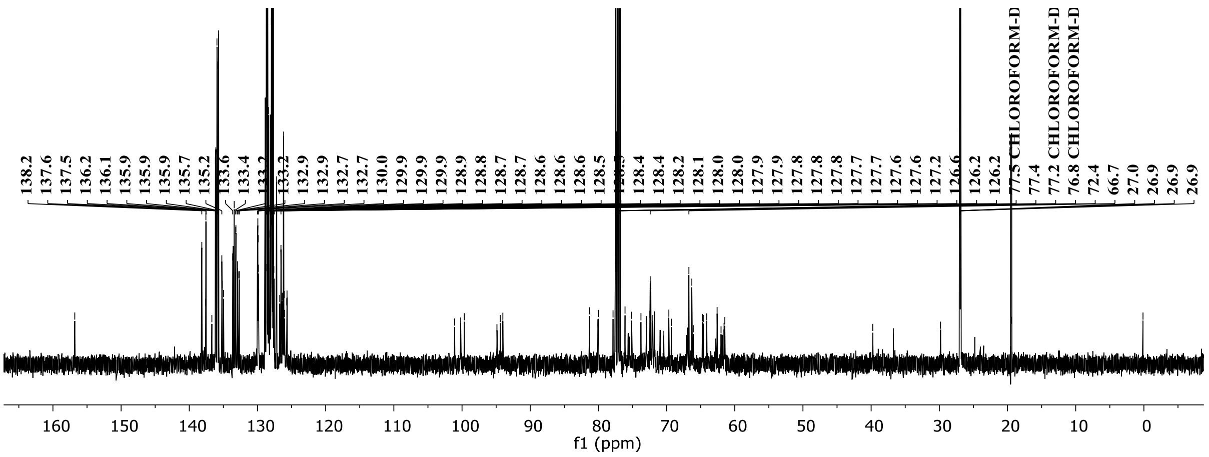
CHLOROFORM-D
f1 (ppm)

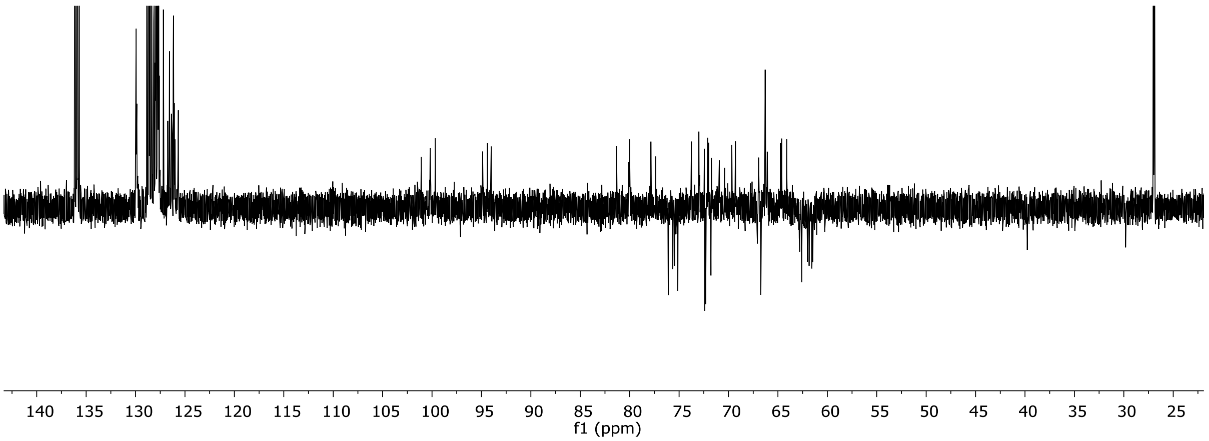
f1 (ppm)

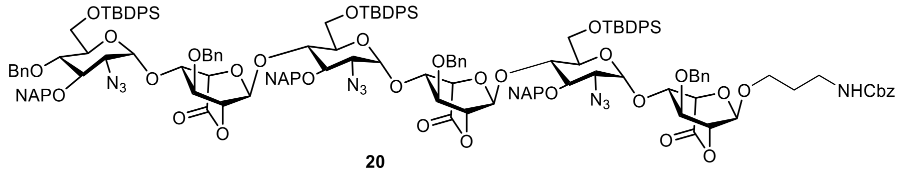
OTBDPS
BnO
NAPO
N3
OBn
O
NAPO
NHCbz

**20**

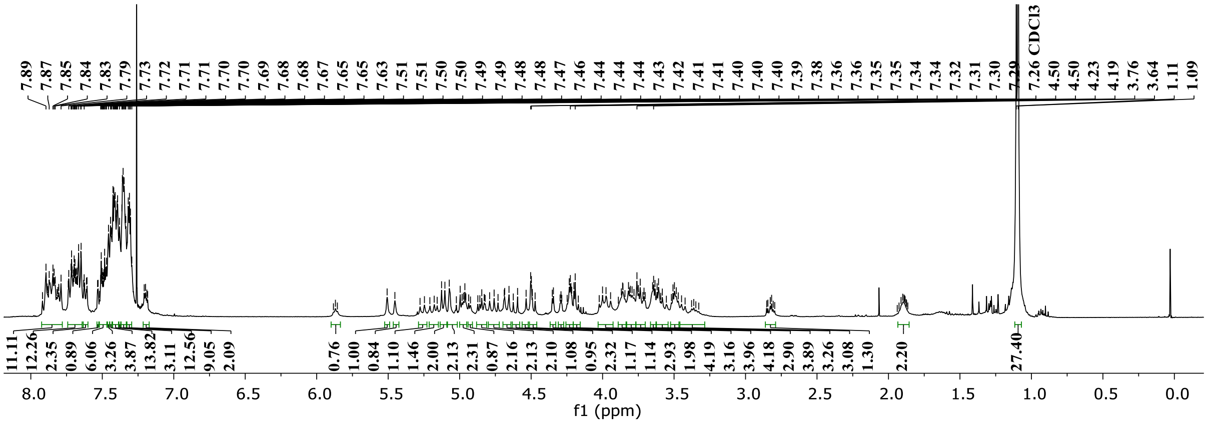
7.89
7.87
7.85
7.84
7.83
7.79
7.73
7.72
7.71
7.71
7.70
7.70
7.69
7.68
7.68
7.67
7.65
7.65
7.63
7.51
7.51
7.50
7.50
7.49
7.49
7.48
7.48
7.47
7.46
7.44
7.44
7.44
7.43
7.42
7.41
7.41
7.40
7.40
7.40
7.39
7.38
7.36
7.36
7.35
7.35
7.34
7.34
7.32
7.31
7.30
7.29
7.26 CDCl3
4.50
4.50
4.23
4.19
3.76
3.64
1.11
1.09
11.11
12.26
2.35
0.89
6.06
3.26
3.87
13.82
3.11
12.56
9.05
2.09
0.76
1.00
0.84
1.10
1.46
2.00
2.13
2.31
0.87
2.16
2.13
2.10
1.08
0.95
2.32
1.17
1.14
2.93
1.98
4.19
3.16
3.96
4.18
2.90
3.89
3.26
3.08
1.30
2.20
27.40
8.0 7.5 7.0 6.5 6.0 5.5 5.0 4.5 4.0 3.5 3.0 2.5 2.0 1.5 1.0 0.5 0.0
f1 (ppm)

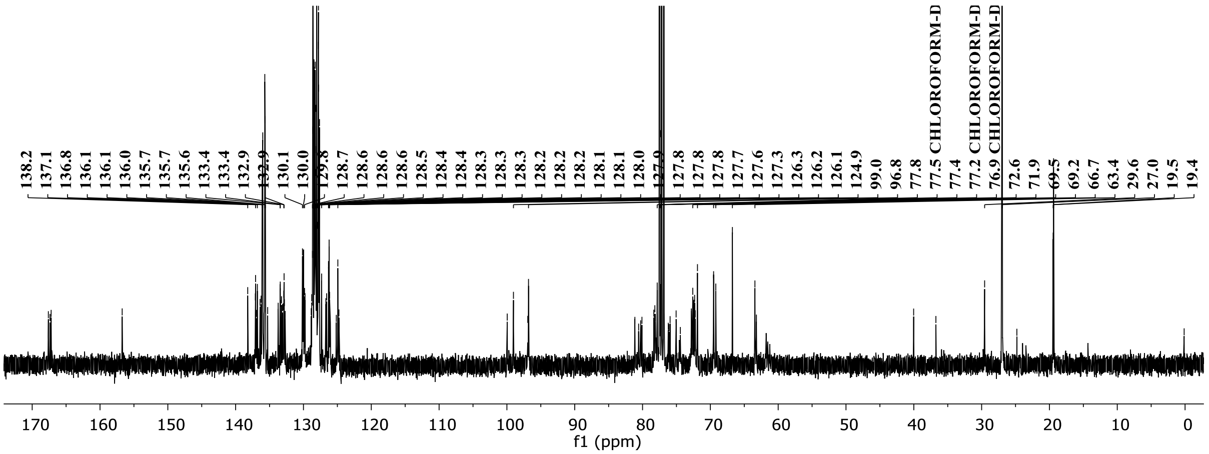
138.2
137.1
136.8
136.1
136.1
136.0
135.7
135.7
135.6
133.4
133.4
132.9
132.9
130.1
130.0
129.8
128.7
128.6
128.6
128.5
128.4
128.4
128.3
128.3
128.3
128.2
128.2
128.1
128.0
127.9
127.8
127.8
127.7
127.6
127.3
126.3
126.2
126.1
124.9
99.0
96.8
77.8
77.5 CHLOROFORM-D
77.4
77.2 CHLOROFORM-D
76.9 CHLOROFORM-D
72.6
71.9
69.5
69.2
66.7
63.4
29.6
27.0
19.5
19.4
170 160 150 140 130 120 110 100 90 80 70 60 50 40 30 20 10 0
f1 (ppm)

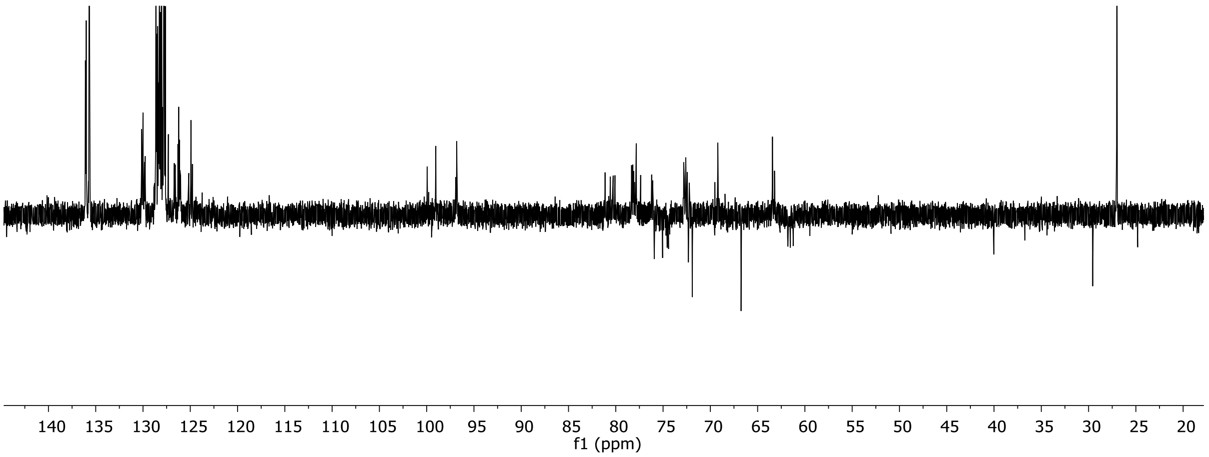
140 135 130 125 120 115 110 105 100 95 90 85 80 75 70 65 60 55 50 45 40 35 30 25 20
f1 (ppm)

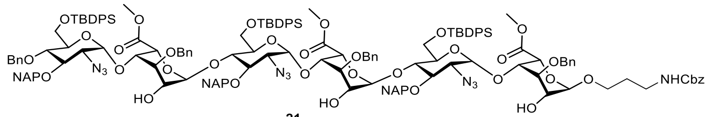
OTBDPS
BnO
NAPO
N3
OBn
HO
NHCbz

**21**

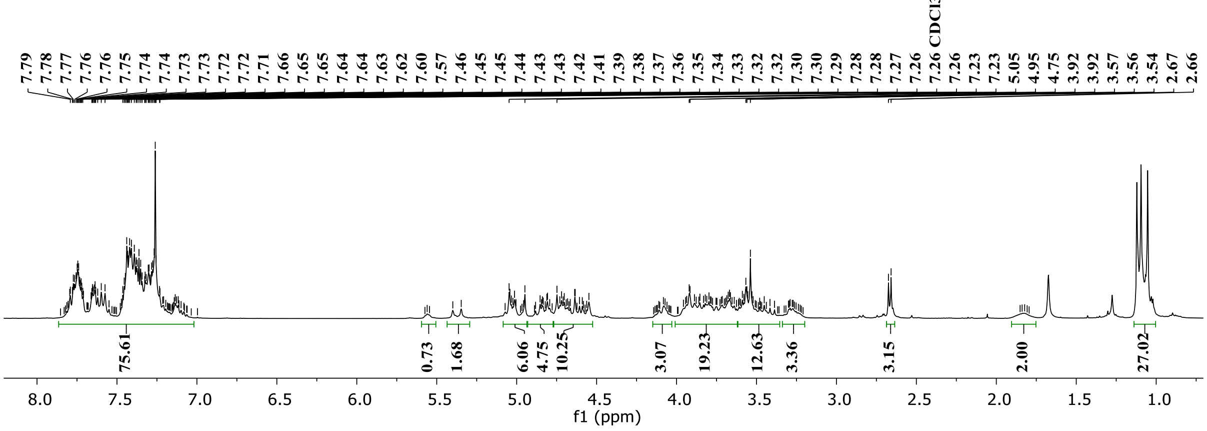
7.79 7.78 7.77 7.76 7.76 7.75 7.74 7.74 7.73 7.73 7.72 7.72 7.71 7.66 7.65 7.65 7.64 7.64 7.63 7.62 7.60 7.57 7.46 7.45 7.45 7.44 7.43 7.43 7.42 7.41 7.39 7.38 7.37 7.36 7.35 7.34 7.33 7.32 7.32 7.30 7.30 7.29 7.28 7.28 7.27 7.26 7.26 7.26 7.23 7.23 5.05 4.95 4.75 3.92 3.92 3.57 3.56 3.54 2.67 2.66
CDCl3
75.61
0.73
1.68
6.06
4.75
10.25
3.07
19.23
12.63
3.36
3.15
2.00
27.02
8.0 7.5 7.0 6.5 6.0 5.5 5.0 4.5 4.0 3.5 3.0 2.5 2.0 1.5 1.0
f1 (ppm)

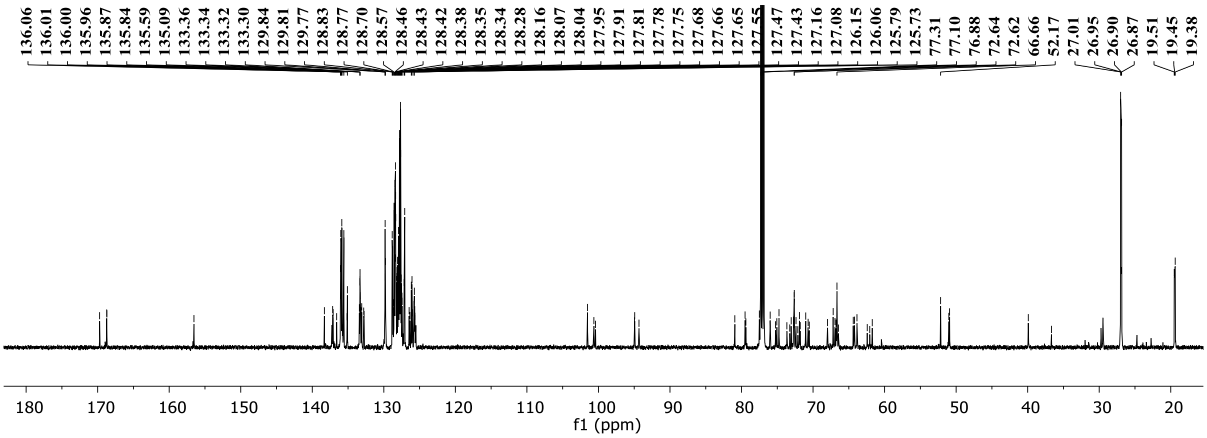
136.06 136.01 136.00 135.96 135.87 135.84 135.59 135.09 133.36 133.34 133.32 133.30 129.84 129.81 129.77 128.83 128.77 128.70 128.57 128.46 128.43 128.42 128.38 128.35 128.34 128.28 128.16 128.07 128.04 127.95 127.91 127.81 127.78 127.75 127.68 127.66 127.65 127.55 127.47 127.43 127.16 127.08 126.15 126.06 125.79 125.73 77.31 77.10 76.88 72.64 72.62 66.66 52.17 27.01 26.95 26.90 26.87 19.51 19.45 19.38
180 170 160 150 140 130 120 110 100 90 80 70 60 50 40 30 20
f1 (ppm)

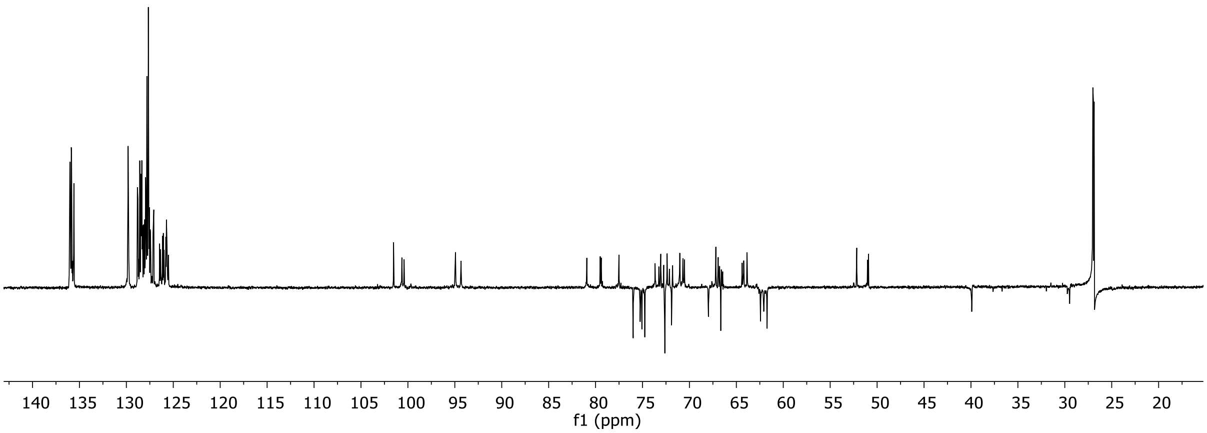
140 135 130 125 120 115 110 105 100 95 90 85 80 75 70 65 60 55 50 45 40 35 30 25 20
f1 (ppm)

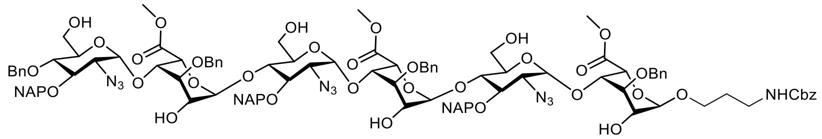
OH
O
BnO
NAPO
$N_3$
OBn
HO
NHCbz

**22**

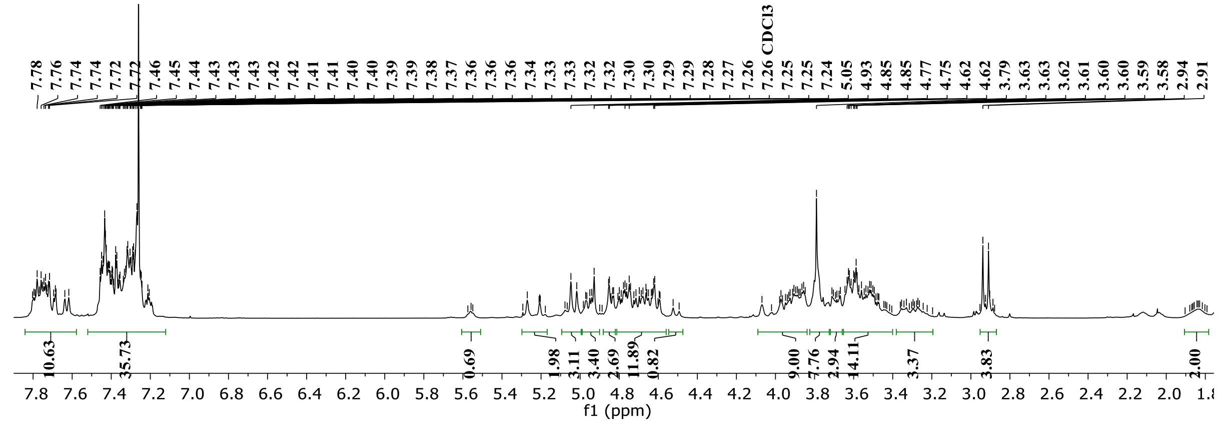
7.78 7.76 7.74 7.74 7.72 7.72 7.46 7.45 7.44 7.43 7.43 7.43 7.42 7.42 7.41 7.41 7.40 7.39 7.39 7.38 7.37 7.36 7.36 7.36 7.34 7.33 7.33 7.32 7.32 7.30 7.30 7.29 7.29 7.28 7.27 7.26 7.26 CDCl3 7.25 7.25 7.24 5.05 4.93 4.85 4.85 4.77 4.75 4.62 4.62 3.79 3.63 3.63 3.62 3.61 3.60 3.60 3.59 3.58 2.94 2.91
10.63 35.73 0.69 1.98 3.11 3.40 2.69 11.89 0.82 9.00 7.76 2.94 14.11 3.37 3.83 2.00
7.8 7.6 7.4 7.2 7.0 6.8 6.6 6.4 6.2 6.0 5.8 5.6 5.4 5.2 5.0 4.8 4.6 4.4 4.2 4.0 3.8 3.6 3.4 3.2 3.0 2.8 2.6 2.4 2.2 2.0
f1 (ppm)

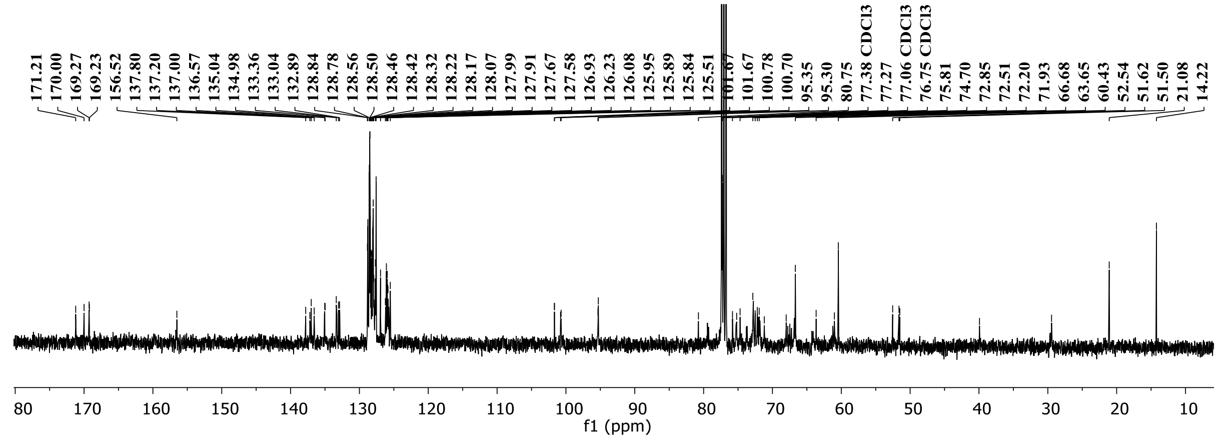
171.21 170.00 169.27 169.23 156.52 137.80 137.20 137.00 136.57 135.04 134.98 133.36 133.04 132.89 128.84 128.78 128.56 128.50 128.46 128.42 128.32 128.22 128.17 128.07 127.99 127.91 127.67 127.58 126.93 126.23 126.08 125.95 125.89 125.84 125.51 101.67 100.78 100.70 95.35 95.30 80.75 77.38 CDCl3 77.27 77.06 CDCl3 76.75 CDCl3 75.81 74.70 72.85 72.51 72.20 71.93 66.68 63.65 60.43 52.54 51.62 51.50 21.08 14.22
170 160 150 140 130 120 110 100 90 80 70 60 50 40 30 20 10
f1 (ppm)

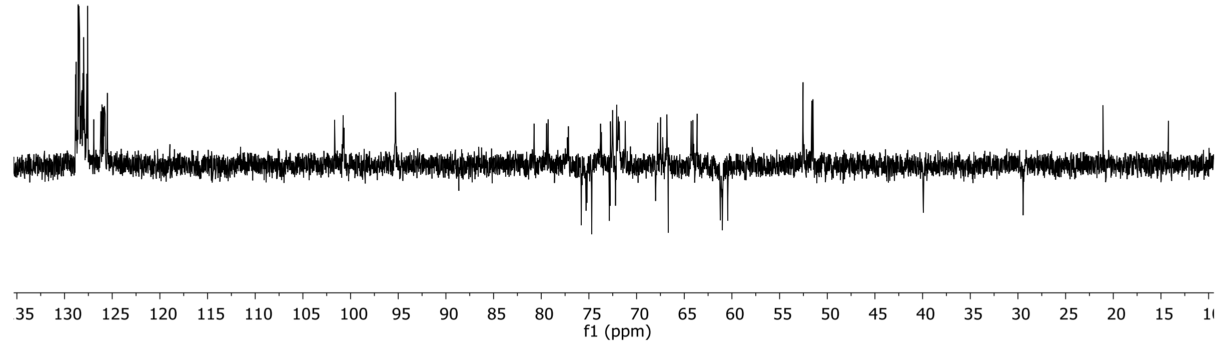
130 125 120 115 110 105 100 95 90 85 80 75 70 65 60 55 50 45 40 35 30 25 20 15
f1 (ppm)

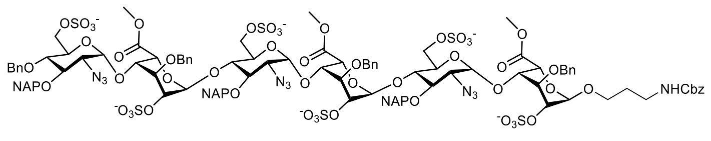
OSO3-
BnO
NAPO
N3
OBn
-O3SO
NHCbz

**23**

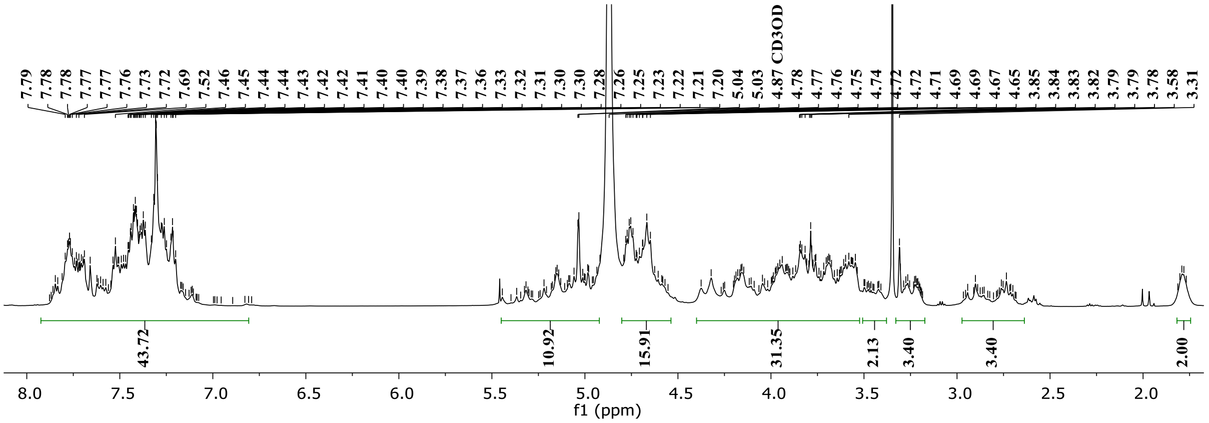
7.79 7.78 7.78 7.77 7.77 7.76 7.73 7.72 7.69 7.52 7.46 7.45 7.44 7.44 7.43 7.42 7.42 7.41 7.40 7.40 7.39 7.38 7.37 7.36 7.33 7.32 7.31 7.30 7.30 7.28 7.26 7.25 7.23 7.22 7.21 7.20 5.04 5.03 4.87 CD3OD 4.78 4.77 4.76 4.75 4.74 4.72 4.72 4.71 4.69 4.69 4.67 4.65 3.85 3.84 3.83 3.82 3.79 3.78 3.58 3.31
43.72
10.92
15.91
31.35
2.13
3.40
3.40
2.00
8.0 7.5 7.0 6.5 6.0 5.5 5.0 4.5 4.0 3.5 3.0 2.5 2.0
f1 (ppm)

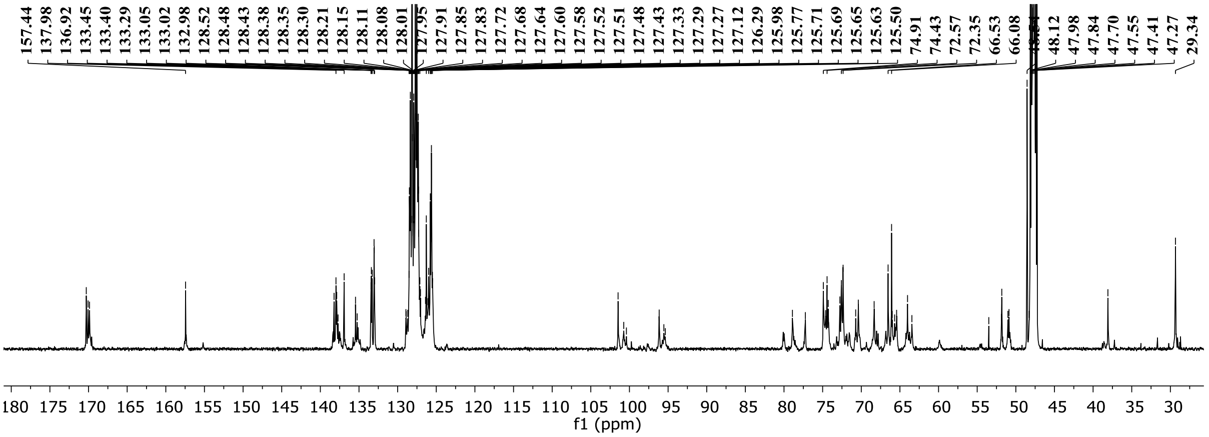
157.44 137.98 136.92 133.45 133.40 133.29 133.05 133.02 132.98 128.52 128.48 128.43 128.38 128.35 128.30 128.21 128.15 128.11 128.08 128.01 127.95 127.91 127.85 127.83 127.72 127.68 127.64 127.60 127.58 127.52 127.51 127.48 127.43 127.33 127.29 127.27 127.12 126.29 125.98 125.77 125.71 125.69 125.65 125.63 125.50 74.91 74.43 72.57 72.35 66.53 66.08 48.12 47.98 47.84 47.70 47.55 47.41 47.27 29.34
180 175 170 165 160 155 150 145 140 135 130 125 120 115 110 105 100 95 90 85 80 75 70 65 60 55 50 45 40 35 30
f1 (ppm)

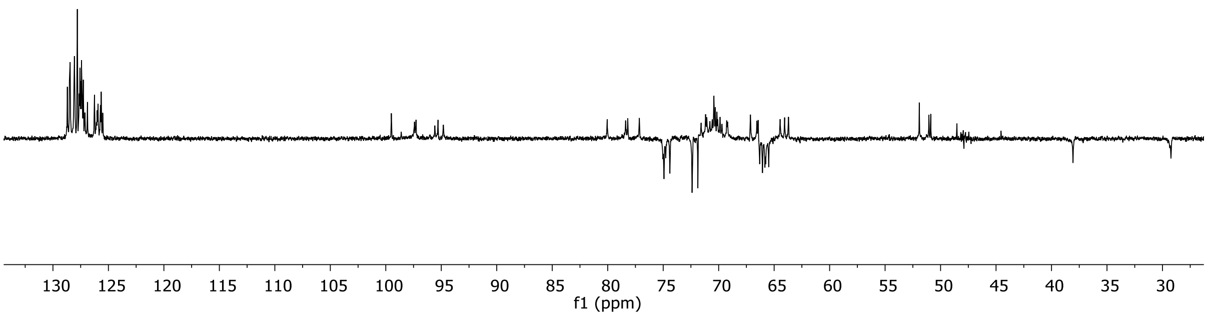
130 125 120 115 110 105 100 95 90 85 80 75 70 65 60 55 50 45 40 35 30
f1 (ppm)

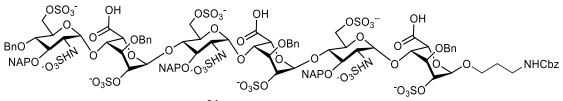
OSO3-
OH
OBn
BnO
NAPO
-O3SHN
-O3SO
NHCbz

**24**

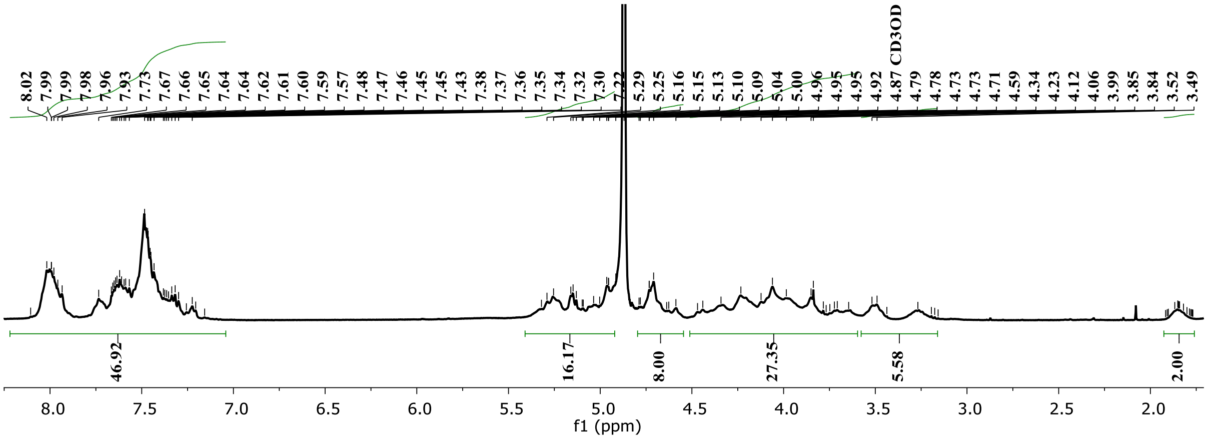
8.02 7.99 7.99 7.98 7.96 7.93 7.73 7.67 7.66 7.65 7.64 7.64 7.62 7.61 7.60 7.59 7.57 7.48 7.47 7.46 7.45 7.45 7.43 7.38 7.37 7.36 7.35 7.34 7.32 7.30 7.22 5.29 5.25 5.16 5.15 5.13 5.10 5.09 5.04 5.00 4.96 4.95 4.95 4.92 4.87 4.79 4.78 4.73 4.73 4.71 4.59 4.34 4.23 4.12 4.06 3.99 3.85 3.84 3.52 3.49
CD3OD
46.92
16.17
8.00
27.35
5.58
2.00
f1 (ppm)

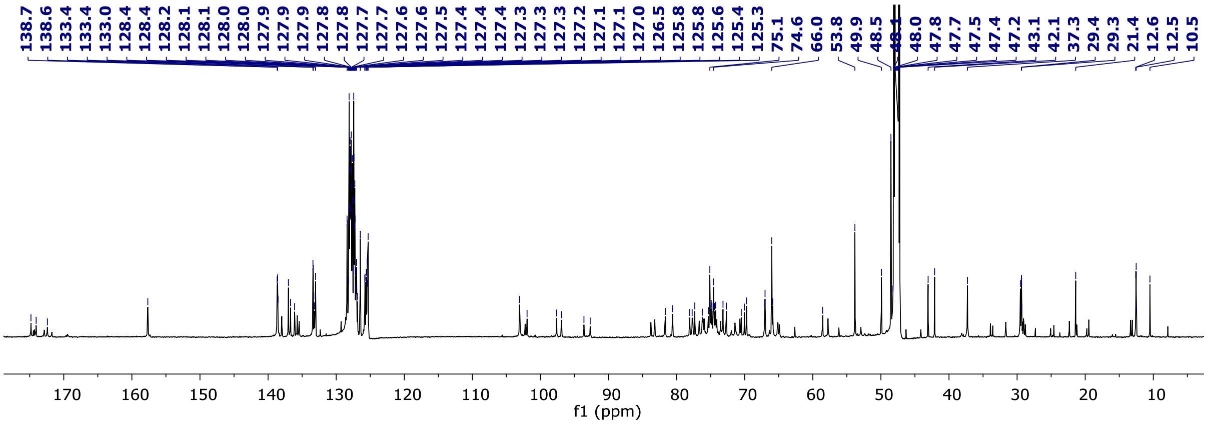
f1 (ppm)

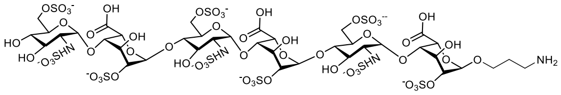

**HSA-6**

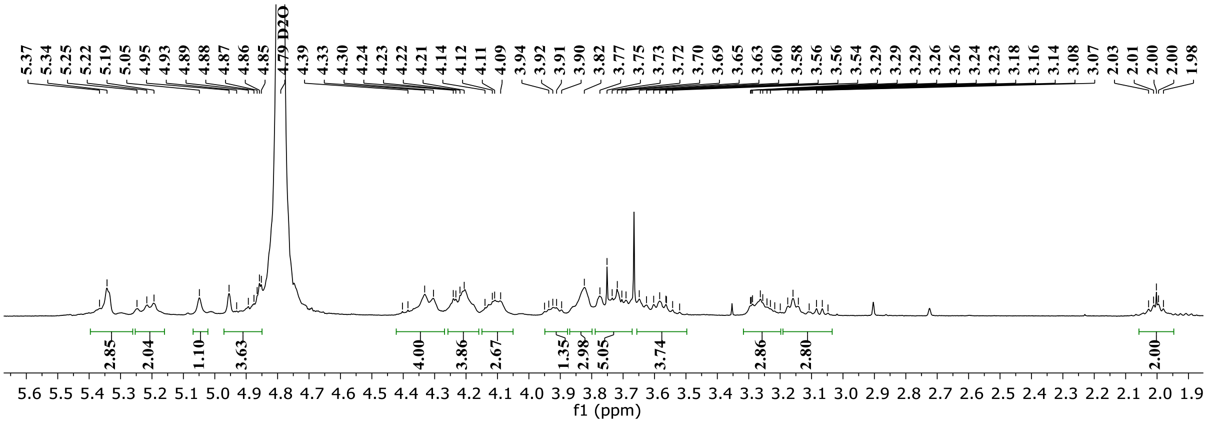
5.37 5.34 5.25 5.22 5.19 5.05 4.95 4.93 4.89 4.88 4.87 4.86 4.85 4.79 D2O 4.39 4.33 4.30 4.24 4.23 4.22 4.21 4.14 4.12 4.11 4.09 3.94 3.92 3.91 3.90 3.82 3.77 3.75 3.73 3.72 3.70 3.69 3.65 3.63 3.60 3.58 3.56 3.56 3.54 3.29 3.29 3.29 3.26 3.26 3.24 3.23 3.18 3.16 3.14 3.08 3.07 2.03 2.01 2.00 2.00 1.98
2.85 2.04 1.10 3.63 4.00 3.86 2.67 1.35 2.98 5.05 3.74 2.86 2.80 2.00
5.6 5.5 5.4 5.3 5.2 5.1 5.0 4.9 4.8 4.7 4.6 4.5 4.4 4.3 4.2 4.1 4.0 3.9 3.8 3.7 3.6 3.5 3.4 3.3 3.2 3.1 3.0 2.9 2.8 2.7 2.6 2.5 2.4 2.3 2.2 2.1 2.0 1.9
f1 (ppm)

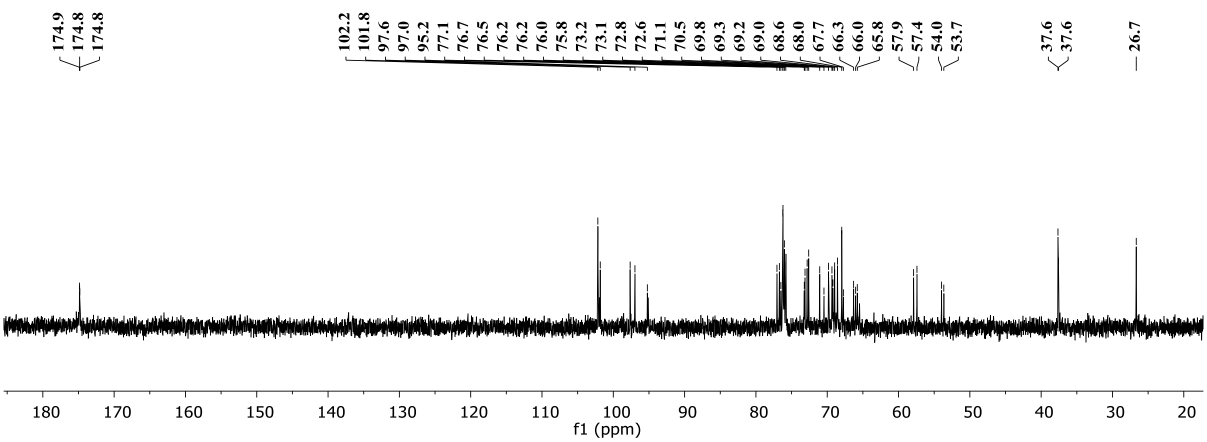
174.9 174.8 174.8
102.2 101.8 97.6 97.0 95.2 77.1 76.7 76.5 76.2 76.2 76.0 75.8 73.2 73.1 72.8 72.6 71.1 70.5 69.8 69.3 69.2 69.0 68.6 68.0 67.7 66.3 66.0 65.8 57.9 57.4 54.0 53.7 37.6 37.6 26.7
180 170 160 150 140 130 120 110 100 90 80 70 60 50 40 30 20
f1 (ppm)

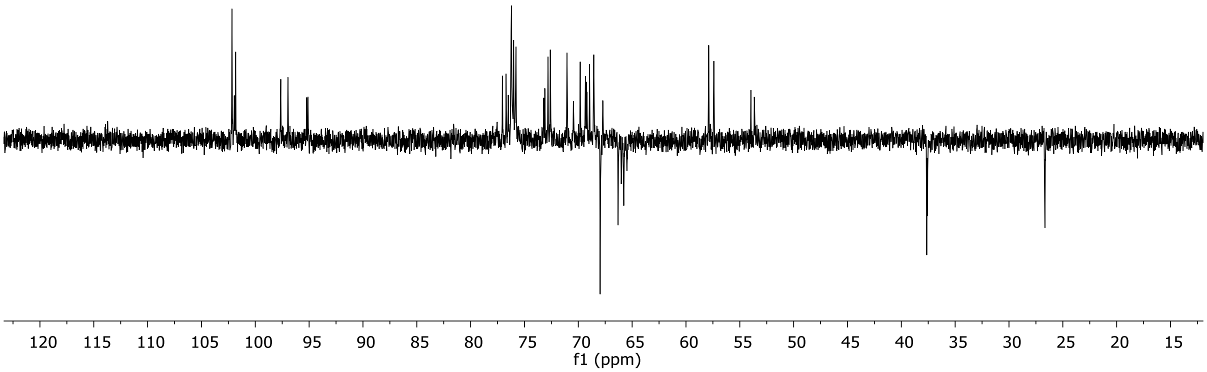
120 115 110 105 100 95 90 85 80 75 70 65 60 55 50 45 40 35 30 25 20 15
f1 (ppm)

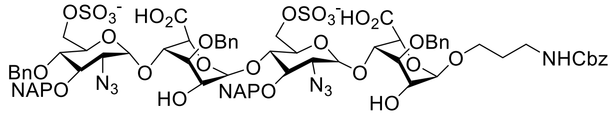

OSO3-
HO2C
OBn
BnO
NAPO
N3
HO
OSO3-
HO2C
OBn
NAPO
N3
HO
NHCbz


**12**

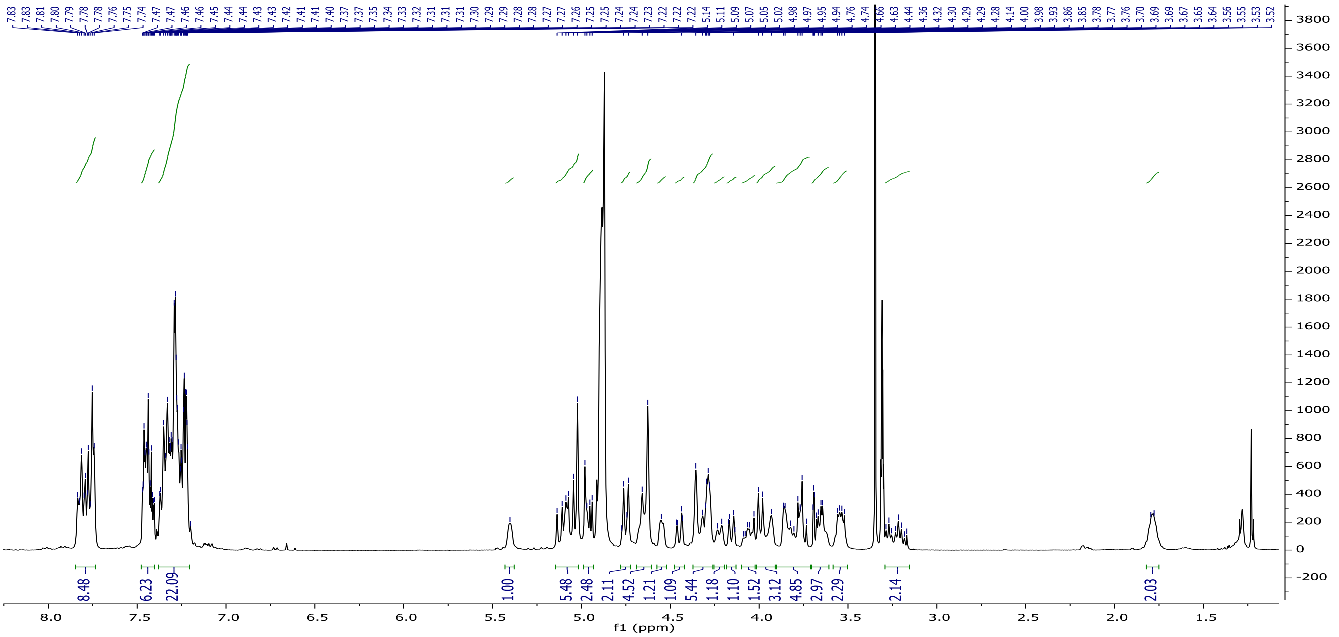

f1 (ppm)


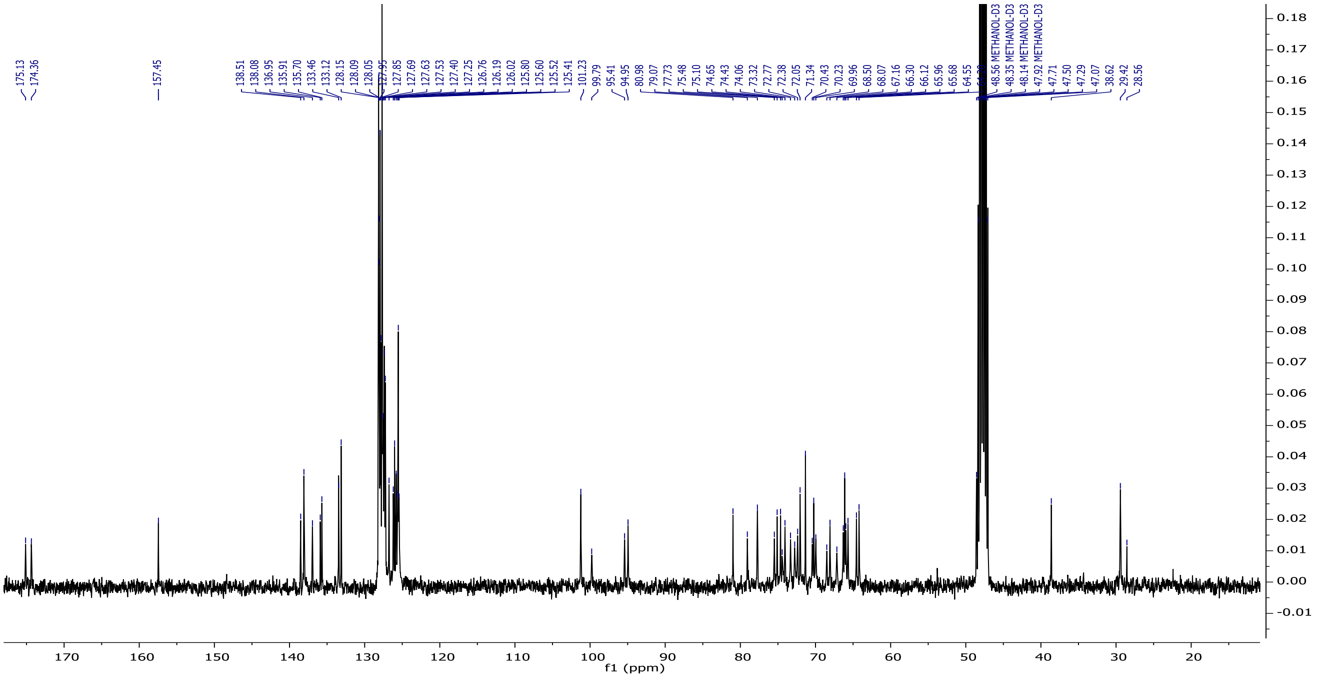

f1 (ppm)

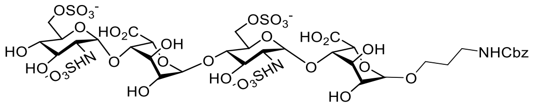
OSO3-
HO2C
OH
HO
HO
-O3SHN
O
HO
NHCbz

**13**

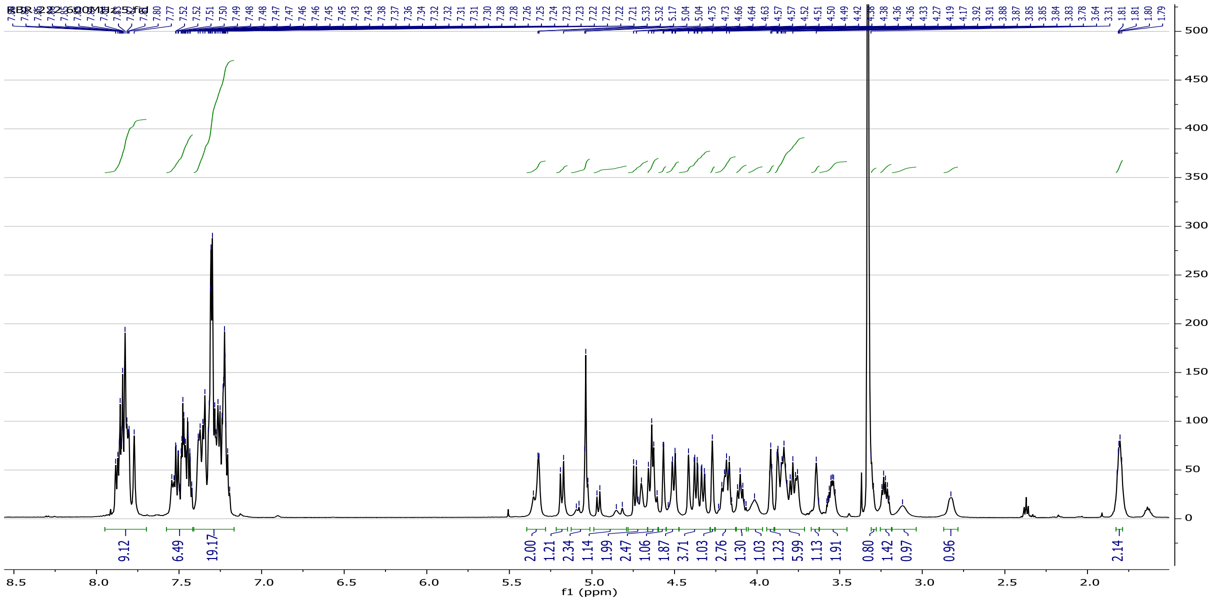
f1 (ppm)

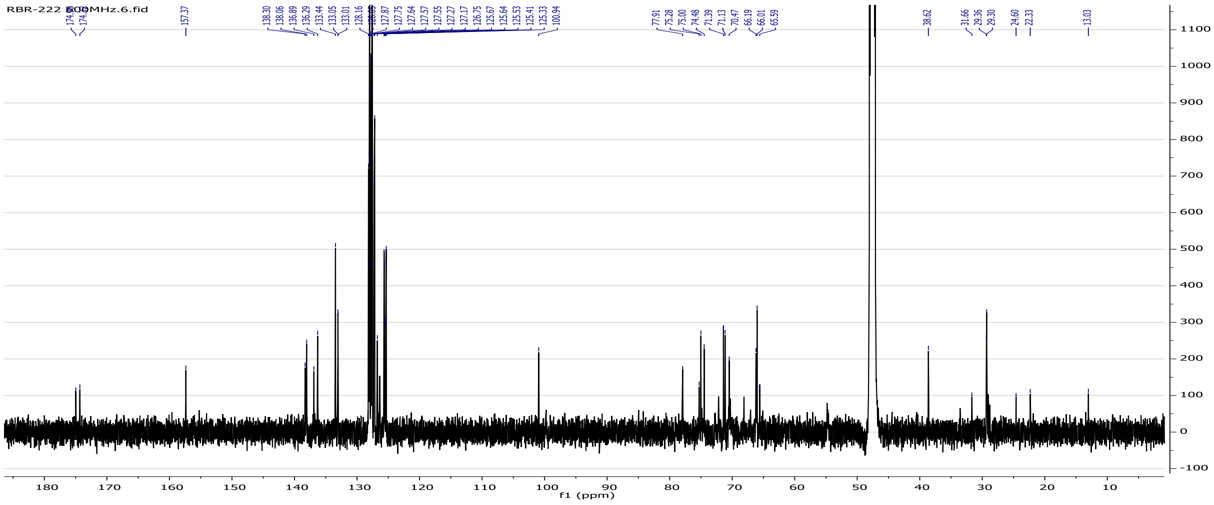
RBR-222 600MHz.6.fid
f1 (ppm)

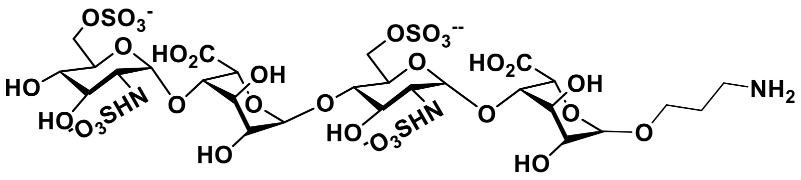
OSO3-
HO2C
OH
HO
HO
-O3SHN
O
HO
OSO3-
HO2C
OH
HO
-O3SHN
O
HO
NH2

**HSA-5**

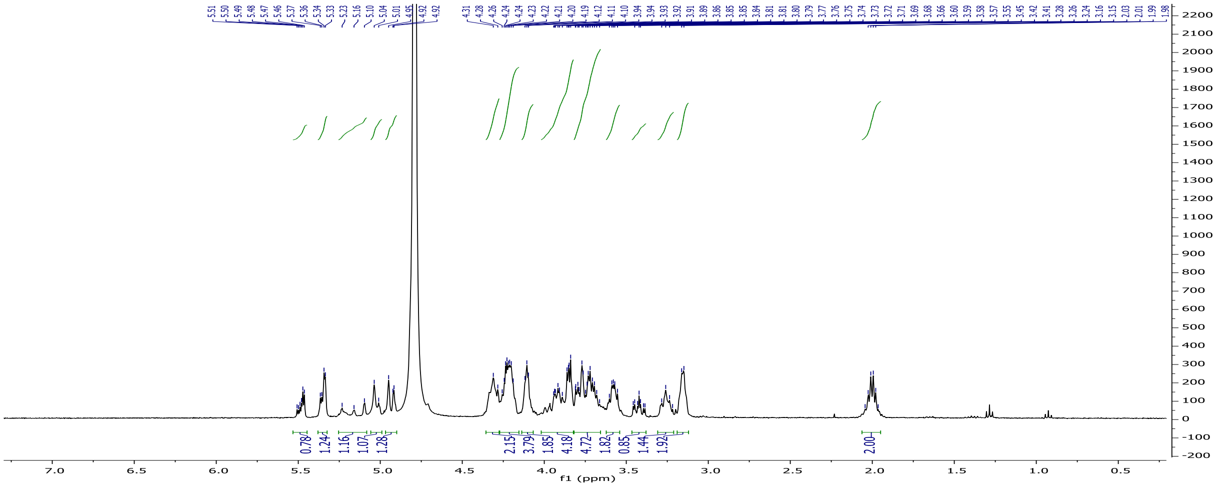
f1 (ppm)

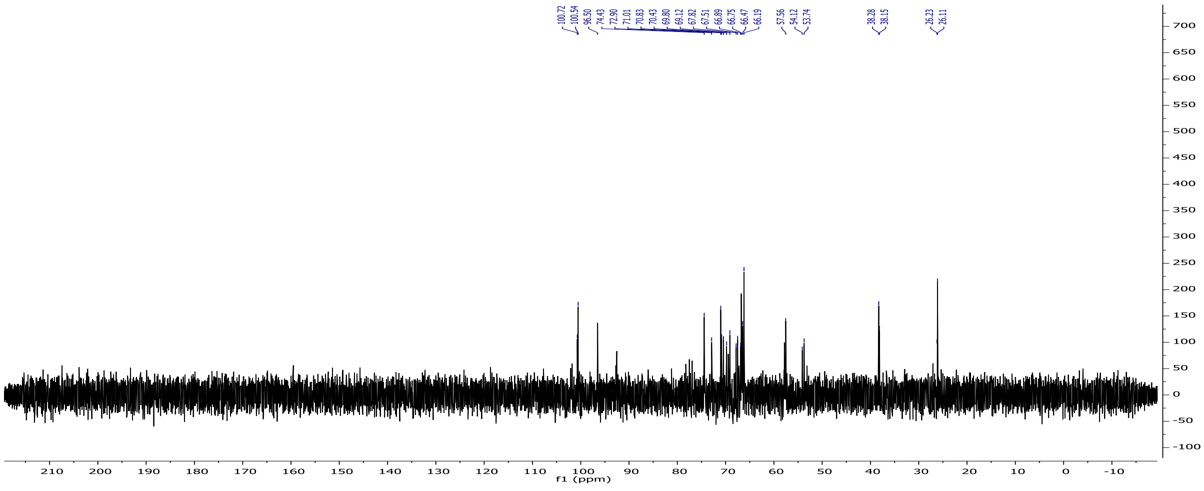
f1 (ppm)

## 20. References


1. Avinoam, O., Fridman, K., Valansi, C., Abutbul, I., Zeev-Ben-Mordehai, T., Maurer, U.E., Sapir, A., Danino, D., Grünewald, K., White, J.M. et al. Conserved Eukaryotic Fusogens Can Fuse Viral Envelopes to Cells. *Science* **332**, 589-592 (2011).
2. Shang, J., Ye, G., Shi, K., Wan, Y., Luo, C., Aihara, H., Geng, Q., Auerbach, A. & Li, F. Structural basis of receptor recognition by SARS-CoV-2. *Nature* **581**, 221-224 (2020).
3. Lahav-Mankovski, N., Prasad, P.K., Oppenheimer-Low, N., Raviv, G., Dadosh, T., Unger, T., Salame, T.M., Motiei, L. & Margulies, D. Decorating bacteria with self-assembled synthetic receptors. *Nat. Commun.* **11**, 1299 (2020).
4. Hatai, J., Prasad, P.K., Lahav-Mankovski, N., Oppenheimer-Low, N., Unger, T., Sirkis, Y.F., Dadosh, T., Motiei, L. & Margulies, D. Assessing changes in the expression levels of cell surface proteins with a turn-on fluorescent molecular probe. *Chem. Commun.* **57**, 1875-1878 (2021).
5. Khan, S., Partuk, E.O., Chiaravalli, J., Kozer, N., Shurrush, K.A., Elbaz-Alon, Y., Scher, N., Giraud, E., Tran-Rajau, J., Agou, F. et al. High-throughput screening identifies broad-spectrum Coronavirus entry inhibitors. *iScience* **27**, 110019 (2024).
6. Whitt, M.A. Generation of VSV pseudotypes using recombinant ΔG-VSV for studies on virus entry, identification of entry inhibitors, and immune responses to vaccines. *J. Virol. Methods* **169**, 365-374 (2010).
7. Stringer, C., Wang, T., Michaelos, M. & Pachitariu, M. Cellpose: a generalist algorithm for cellular segmentation. *Nat. Methods* **18**, 100-106 (2021).
8. Jain, P., Shanthamurthy, C.D., Leviatan Ben-Arye, S., Woods, R.J., Kikkeri, R. & Padler-Karavani, V. Discovery of rare sulfated N-unsubstituted glucosamine based heparan sulfate analogs selectively activating chemokines. *Chem. Sci.* **12**, 3674-3681 (2021).
9. Vishweshwara, S.S., Bhoge, P.R., Anand, S., Raigawali, R., Chandra, A., Saladi, S.V. & Kikkeri, R. Immunogenic Sulfated l-Idose Homo Oligosaccharides Elicit Neutralizing Antibody against Native Heparan Sulfate with Biomarker and Therapeutic Possibilities. *J. Med. Chem.* **67**, 18465-18477 (2024).
10. Chhabra, M., Shanthamurthy, C.D., Kumar, N.V., Mardhekar, S., Vishweshwara, S.S., Wimmer, N., Modhiran, N., Watterson, D., Amarilla, A.A., Cha, J.S. et al. Amphiphilic Heparinoids as Potent Antiviral Agents against SARS-CoV-2. *J. Med. Chem.* **67**, 11885-11916 (2024).
11. Jain, P., Shanthamurthy, C.D., Chaudhary, P.M. & Kikkeri, R. Rational designing of glyco-nanovehicles to target cellular heterogeneity. *Chem. Sci.* **12**, 4021-4027 (2021).
